# Where Are the Irish in Research on Ethnic Health Inequalities in Britain? A State‐Of‐The‐Art Literature Review

**DOI:** 10.1111/1467-9566.13874

**Published:** 2025-01-02

**Authors:** Rosalind Willis

**Affiliations:** ^1^ Department of Gerontology University of Southampton Southampton UK

**Keywords:** Britain, critical, health, Irish, migration, review

## Abstract

Decisions about ethnic groups studied in health research shape recommendations. If a group is not included in research, its ability to call for policy change is limited. Despite health inequalities for the Irish in Britain in the 20th century, recent research on health is likely to combine the White Irish with White British, whereas Irish people of colour are not mentioned at all. This paper aims to understand why the interest in this group has declined. A state‐of‐the‐art literature review of 140 papers on Irish health in Britain from 2001 to 2023 was conducted. Findings show the Irish are still disadvantaged in mortality, mental health and suicide, with important socioeconomic status and gender disparities. The shifting way the Irish are constructed over time is critically examined, paying attention to historical depictions and postcolonial identities. Sociological theories of migration are challenged by the Irish because this migrant group does not conform to theoretical assumptions. The Irish may have dropped from the agenda because of incorrect assumptions about assimilation and the relationship between Ireland and the UK. Given that the health outcomes of the Irish in Britain differ from those of the White British population, they should be recognised in health research as a distinct group.

## 1 Introduction

This article discusses the Irish in Britain: people born on the island of Ireland or their descendants who live in Great Britain. This includes (i) people who ethnically identify as ‘White Irish’ (*n* = 507,473, 0.9% of the population of England & Wales; *n* = 56,877, 1.05% in Scotland), (ii) ‘White Gypsy or Irish Traveller’ (*n* = 67,757, 0.1% in England & Wales; *n* = 3343, 0.06% in Scotland) (ONS 2022; ScotPHO 2024), (iii) Irish people of colour or mixed heritage, who are hidden by the Census ethnicity categories or (iv) descendants of Irish people. The article therefore conceptualises ethnicity as an identity reflecting historical and individual experiences, rather than an unproblematic reflection of the discredited biological concept of ‘race’ (Nazroo, Bhui, and Rhodes 2020). The White Irish population in England and Wales has the oldest age structure (ONS 2023), due to the ageing of immigrant cohorts and low fertility (Tilki et al. 2009) but also due to some second‐generation Irish not identifying as ethnically White Irish (Lievesley 2010; Willis 2017).

Long‐established evidence reveals health disparities between the Irish in Britain and the general population. For example, the Irish had higher mortality rates (Raftery, Jones, and Rosato 1990), suicide rates (Balarajan 1995), were more likely to have psychiatric admissions (Cochrane and Bal 1989) or to misuse alcohol (Evandrou 2000). Many of these issues cross generations (Harding and Balarajan 1996).

A group with an ethnic minority status is better able to have its specific needs met (Nagel 1994). The Greater London Council (1984) called for the recognition of the Irish as an ethnic minority group, whereas the Commission for Racial Equality (M. J. Hickman and Walter 1997) recommended including an Irish category in official forms to allow better data collection. Turning to health research, Aspinall argued that the single White category in the 1991 Census was problematic because of heterogeneity and advocated for recognising the Irish (Aspinall 1998). Furthermore, regarding the usefulness of the 2001 Census for monitoring health inequalities, he said that the new White Irish category was welcome but that the complexities of Irish identity (e.g., Northern Irish) would not be captured (Aspinall 2000). Nonetheless, the 2001 Census marked a change in official data collection that would have implications for subsequent health research. However, there seems to have been a decline over time in including the Irish in Britain in studies of ethnic health inequalities.

More recently, much ethnicity research obscures the White Irish distinctiveness, while Irish people of colour are not considered. For example, the Health Survey for England 2004 included the Irish in their ethnic minority boost sample, but Understanding Society 2009 did not. In the foreword to the King's Fund ‘Ethnic health inequalities and the NHS’ Robertson et al. (2021: 4) report: ‘*We have seen Black and minority ethnic groups continue to lag behind their White counterparts in terms of living long and healthy lives*’. In a later King's Fund (Raleigh 2023) report on the health of ethnic minorities only two of the seven conditions (mortality and obesity) had data for the White Irish. For other conditions, the White Irish were put into an ‘Other White’ or a ‘White’ group. The authors were limited by the original data sources, but this is indicative of the wider omission of the Irish from studies and from ethnic minority boost samples. Furthermore, some refer to ‘White British/Irish’ as if there is no difference between the two (Evans et al. 2018). For example, Jayaweera and Quigley (2010: 1004) using the millennium cohort study to examine the health of migrant mothers combined White British and White Irish participants into a single group arguing that ‘*We do not think that in general there are serious implications of combining mothers classifying themselves as White British and as White Irish … because we felt that they could be distinguished from other migrant ethnic group categories historically*, *and for reasons of small sample size among White Irish*’.

This trend is problematic because the opportunities to monitor whether the Irish experience health inequalities are reducing. Knowing this information is important for policy makers, health and social care services and third‐sector organisations to help them address the needs of Irish people.

## 2 Aims and Research Questions

This article examines literature on the health of the Irish in Britain since the introduction of the White Irish census category in 2001, to assess whether the Irish population in Britain still has a health disadvantage, and to understand reasons for the apparent decline in research interest.

The research questions are as follows:

- What do we know about the current state of health of Irish people in Britain?
  - Are the Irish still disadvantaged in terms of health?
- Has there been a decline in research on the health of the Irish in Britain over the last 20 years?
  - If so, why?

Sociological theories of migration and health (e.g., healthy migrant effect, assimilation) are challenged by the Irish because this migrant group does not conform to theoretical expectations. Alternative theories have been employed to explain patterns of health and illness, including individual factors such as ethnic identity, salutogenesis or social capital, and societal factors such as structural inequality, racism or postcolonialism. The literature on ethnicity and health shows an ambivalence towards recognising the Irish as a minority ethnic group in Britain, and arguments from literature on whiteness and Irishness are mobilised to refute this ambivalence. The shifting way the Irish are constructed as an ethnic group over time is critically examined, paying attention to historical depictions and postcolonial identities. This article contributes to the field of sociology of health and illness by critically synthesising and deconstructing two decades of literature on Irish health, presents arguments to explain the slow erosion of the Irish from the ethnicity and health discourse and draws attention to the detrimental implications of obscuring ethnic distinctiveness when examining health inequalities.

## 3 Methodology

### 3.1 State‐Of‐The‐Art Review

The state‐of‐the‐art literature review approach is suitable when the goals are to understand ‘where we are’ and ‘how we got here’ (Barry, Merkebu, and Varpio 2022). It is necessary to understand the philosophy of review approaches. Systematic reviews have a realist ontology, a positivist epistemology and aim to be objective, replicable and eliminate bias. Other types of reviews (such as the narrative review and the state‐of‐the‐art review) have a relativist orientation, recognise researcher subjectivity and take a critical approach (Barry, Merkebu, and Varpio 2022; Greenhalgh, Thorne, and Malterud 2018; Hammersley 2001). Positivists would view the state‐of‐the‐art review as less reliable and valid. However, as it has a different purpose to systematic reviews, the same criteria should not be applied. The state‐of‐the‐art review takes a rigorous approach to searching the literature, similar in meticulousness to the systematic review, but it is not necessary to log the search strategy on PROSPERO or to use PRISMA diagrams. The process includes reflexivity, recognising that it is not possible to fully eliminate researcher ‘bias.’ No quality check of the literature is required because the aim of the review is to examine how and why research has been conducted rather than find a definitive answer to a ‘what works?’ question (Barry, Merkebu, and Varpio 2022).

### 3.2 Literature Search Strategy

The strategy was co‐designed with a librarian experienced in literature reviews. Databases were searched between February and May 2023 by two people—the author and an assistant. The focus was the health of the Irish in Britain. Two sets of searches used the following key concepts: (i) (Irish AND Britain AND migration/minority status AND health status/conditions) and (ii) (Irish AND Britain AND migration/minority status AND health service use/satisfaction/attitudes). The time period (2001–2023) was selected because 2001 was when the White Irish ethnic group was first included in the Census. Three databases were selected based on their coverage of physical and mental health: Medline, PsycINFO and Web of Science. Medline was searched separately from Web of Science because the EBSCO interface allowed subject terms, which increased the comprehensiveness of the search. Please see the online appendix for more details about the searches.

**FIGURE 1:**
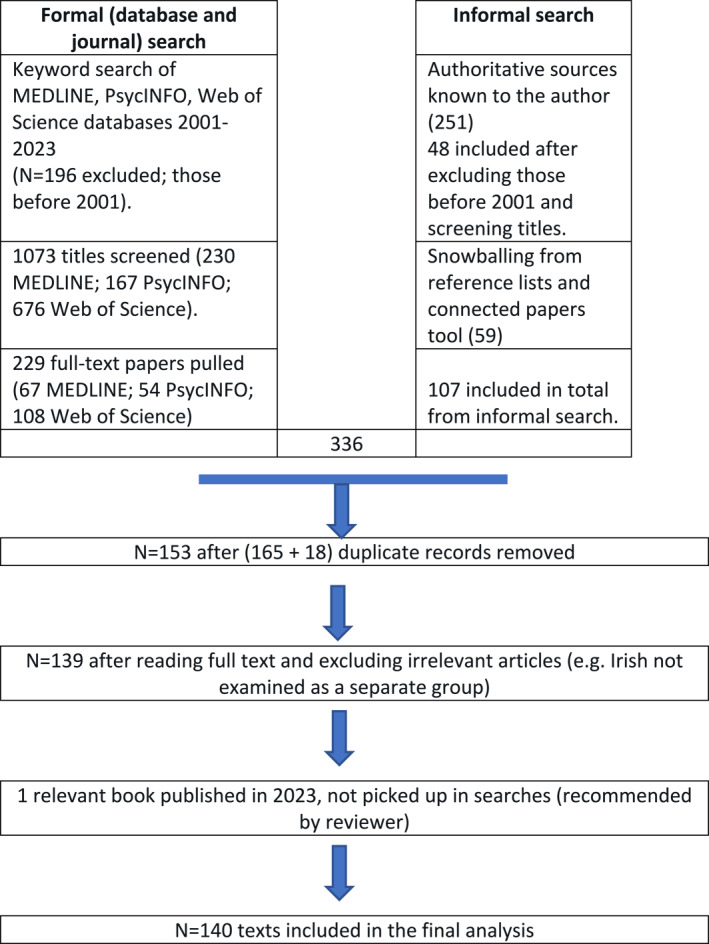
Flow diagram of the filtering process following Greenhalgh's (2023) ‘honest’ diagram.

The database searches were supplemented with (i) snowballing from reference lists, (ii) cross‐checking with literature already known to the author and (iii) using the Connected Papers tool to check for links between articles. The 336 results were saved in EndNote, the automatic duplicate search was performed and 165 duplicates were deleted. Further 18 duplicates were identified by manual checking. On reading the full‐text a further 14 references were eliminated (e.g., because they did not include the Irish). A relevant book was published in 2023 and added to the corpus after the search process was completed, following reviewer recommendation. The final number of references in the corpus was 140 (See the online appendix for list of the full references in Supporting Information S1). Please refer to Figure 1 and Table 1 for the numbers excluded at each stage.

**TABLE 1:** Number of hits and exclusions in each database.

	Search 1	Search 2	Sub‐total	Number published pre‐2001 (and excluded)	Final total
PsycINFO	207	29	236	69	167
MEDLINE	247	50	297	67	230
Web of science	691	45	736	60	676
			1269	196	1073

A table was created to extract information from the 140 sources with columns for objectives, location, sample, methodology, tools, results, theories and construction of the Irish (see the online appendix for an abbreviated version). The results section examines how the Irish were constructed in the literature, theories used to explain health inequalities and the extent to which the Irish are still disadvantaged. The discussion section explores why interest in research the Irish seems to have declined over the period 2001 to 2023.

### 3.3 Reflexivity

I am a White Irish person living in England since the 1990s, a first‐generation migrant with a family history of short‐term emigration to Britain. I am a part of the group under study. I am university‐educated and middle‐class, so I do not fit the profile of the mid‐20th century Irish emigrants, many of whom worked in construction or nursing. I am a closer fit to the Irish emigrants from the latter part of the 20th century. The timing of my migration meant I did not encounter the discrimination of my predecessors, related to racism or terrorism (M. Hickman 1995). However, I do commonly encounter denials that Irish culture is different from British culture, I have been rebuked for taking offence at Irish jokes because they are just meant as ‘banter’, and I have been told I cannot use the term ‘minority ethnic group’ for the ‘White Irish’ because of our whiteness. I am therefore prepared for resistance to my choice to research this particular ethnic group.

I started this literature review with the expectation that interest in the Irish in Britain had declined, based on my observation from literature on ethnic health inequalities that commonly combined White groups into one category. Part‐way through this work my assumptions were challenged by the volume of papers. However, at around the same time there was a funding call from the National Institute for Health and Care Research entitled 'Reducing health inequalities related to ethnicity by influencing the wider determinants of health’, which excluded the White Irish from consideration (NIHR 2023: 12): ‘*For the purpose of this call*, *ethnic minority groups are defined as groups comprising people who do not identify as White British or White Irish*’. Although the NIHR call excluded the White Irish, it did include ‘Gypsy, Roma or Irish Traveller populations’ within the scope of the call. This group has relatively poor health compared to the general population (Irish in Britain, 2023). There is good recognition of the need to include Gypsy, Roma and Irish Travellers in strategies to reduce healthcare inequalities (e.g., NHS England 2021). However, the White Irish group is not prioritised. My literature review can help to establish some of the reasons for this differential consideration of Irish groups.

## 4 Results

### 4.1 Composition of the Literature

There were 140 sources: 93 secondary quantitative analyses, 17 primary qualitative, 11 primary quantitative, six mixed methods, six literature reviews, six discussion articles and one book chapter. The majority were peer‐reviewed journal articles, although a small number were non‐peer‐reviewed reports. Four of the articles had a historical perspective, either discussion articles or secondary analysis of historic psychiatric records.

A range of survey datasets was employed: Ethnic Minority Psychiatric Illness Rates in the Community (EMPIRIC), Health Survey for England (HSE), Understanding Society: UK Household Longitudinal Study, the Census (including ONS Longitudinal Study, Samples of Anonymised Records), Scottish Health and Ethnicity Linkage Study, Scottish Twenty‐07 study, National Child Development Study, Hospital Episode Statistics, UK Biobank, School Census, Fourth National Survey of Ethnic Minorities, general practice patient survey, evidence for equality National survey (EVENS), NHS patient records, and death certificates.

A wide range of health topics was addressed. These include (from most to least common): mental health/well‐being, mortality, use of health services, alcohol, self‐rated health, limiting long‐term illness, how discrimination or racism is linked to health, smoking, cardiovascular health, depression, hospital admissions, suicide, cancer, dementia, health beliefs, childhood trauma from institutional abuse, oral health, respiratory conditions, diabetes, health behaviour (such as exercise and diet), vaccine hesitancy, use of screening services, breast feeding, life expectancy, obesity, place of death, COVID‐19, self‐harm and coronary heart disease. One article each looked at autism, drug use, gastrointestinal issues, kidney health, maternal health, measles, coping strategies and stroke.

**FIGURE 2:**
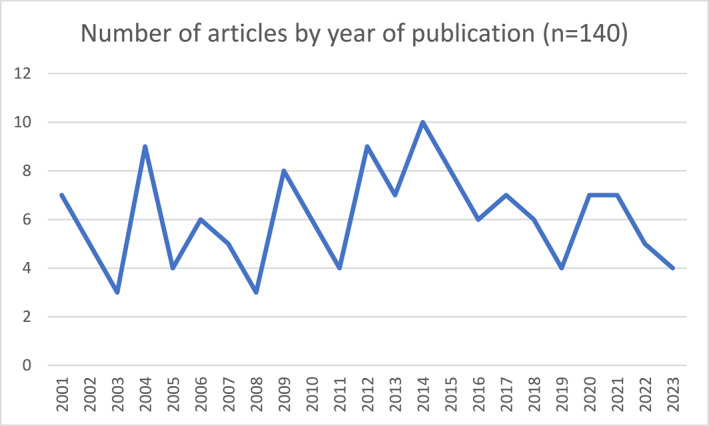
Trend over time.

Figure 2 shows the frequency of papers published in each year. Please note that the results do not include papers that were published after May 2023. Some papers (*n* = 43) had the Irish as the main focus, whereas others included the Irish as part of group comparisons. There are peaks in 2004 and 2014, related to large datasets (e.g., EMPIRIC or HSE) or the activity of specific researchers. There is a reduction in publications over time, but research on the health of the Irish in Britain has not been extinguished.

### 4.2 Construction of the Irish

The category of ‘Irish’ was operationalised in a variety of ways. Earlier articles used ‘Irish’ (not White Irish) meaning country of birth being the Republic of Ireland (or the Irish Free State). A few articles included people born on the island of Ireland (either Republic of Ireland or Northern Ireland) as part of the Irish group (McCambridge et al. 2004; Scanlon et al. 2006). Some used self‐identification as Irish rather than asking people to fit into the census categories (Moore 2019), for example Kelly and Ciclitira (2011) had categories of White Irish, Anglo–Irish, and Egyptian–Irish. Some Scottish articles (Abbotts 2004; Abbotts, Williams, and Ford 2001; Walls and Williams 2003, 2004) used ‘Catholic descent’ as a proxy for Irish, arguing that they descended from Irish famine emigrants. The ethnic group categories in the census of Scotland differ from those used in England & Wales, such that there is no separate ‘White: Northern Irish’ category in Scotland unlike in England & Wales. This could mean that Scottish data has Northern Irish people in either the White: Irish category or the White: Other British category, whereas in England & Wales those who select White: Northern Irish are usually combined with White British in analyses.

The majority of articles in England and Wales focused on first‐generation migrants, although some included second‐ (Das‐Munshi et al. 2013 2014a; Puthussery et al. 2010) and third‐generation (Harding and Balarajan 2001). Two of the surveys (EMPIRIC and HSE 2004) operationalised Irish as either being Irish‐born or having at least one parent born in Ireland, and so included second‐generation Irish (Sproston and Mindell 2006; Weich et al. 2004). In some cases Irish (not White Irish) was used because the source was death records, which recorded country of birth but not ethnicity (Ougrin et al. 2011).

Some authors referred to the Irish as an ethnic minority group (Alobaidi, Bernabe, and Delgado‐Angulo 2022; Commander et al. 2003; Das‐Munshi et al. 2016; Lowe et al. 2020; Moselhy and Telfer 2002; Puthussery et al. 2010; Rao, Schofield, and Ashworth 2015; Twamley et al. 2011), as part of the Black and minority ethnic population (Rao, Wolff, and Marshall 2008; Tilki et al. 2010), part of the Black, Asian and minority ethnic (BAME) population (Tilki 2017), as one of the ‘seven largest minority ethnic groups in England’ (Sproston and Mindell 2006: 2), or as one of the ‘main six ethnic minority groups in the UK’ (Amininia, Bernabe, and Delgado‐Angulo 2023: 61). Others excluded the White Irish from ethnic minority groups: Aldridge et al. (2020) in a study of COVID‐19 compared White British, White Irish and BAME groups. It is not always clear whether these articles use the term ‘minority’ to refer to numerical minorities in the general population or to recognise shared experiences of racially minoritised groups. However, the sample sizes in many of the large surveys of people who identify as Irish (whether it is the White Irish, Irish Travellers, Irish people of colour or Irish descent) is likely to be numerically small, which is partly due to researchers not including Irish people when they design booster samples for ‘ethnic minority’ groups.

Almost all authors assumed that Irish people are White; the HSE used focused enumeration (where householders are asked about the ethnicity of people living either side of their address) but stated that as this only works for ‘visible’ minority groups it was not relevant for Irish people (Sproston and Mindell 2006). In Moselhy and Telfer (2002) Irish are referred to as one of the ‘Caucasian’ groups. Puthussery et al. (2008) discuss ‘invisibility’ of the second‐generation Irish, implying Irish people were white. Leavey and Eliacin (2013: 199) referred to the Irish in Britain as a ‘white ethnic group’. Chum, Teo, and Azra (2022) used a category called ‘Irish (Republic)’ but also referred to them as ‘Irish Whites’.

However, a few articles contrasted the ‘Irish’ group with a ‘White’ (Baker et al. 2013; Rao, Schofield, and Ashworth 2015; Smith, Kelly, and Nazroo 2012) or ‘White general’ (R. S. Bhopal et al. 2013) group. It could be argued that this means the authors did not view the Irish group as white, or that the authors wished to recognise the distinctive experience of Irish people. An early article referred to ‘non‐white groups’ and included the Irish as one of these (Bhui et al. 2005: 499). Only one piece of literature acknowledged that there are Irish people of colour, which was the one of most recent pieces: a secondary analysis of the 2021 Census (Irish in Britain 2023). Even that source was unable to present health data for Irish people of colour, as they were limited by the census data categories. It remains unclear whether the health status of Irish people of colour is similar to other people of colour in Britain.

Some articles made factually incorrect assumptions about the Irish. For example, Landman and Cruickshank (2001) referred to migrants from Ireland to England/Wales as internal migrants, even though the Republic of Ireland (RoI) is not part of the UK. Bansal et al. (2014) used a dichotomous variable of UK/non‐UK born, but their UK category included those born in the ‘British Isles’ which would include the Republic of Ireland. Similarly, R. S. Bhopal et al. (2018) and Cezard et al. (2020) combined UK and RoI country of birth into one category with no justification.

Irish Travellers were usually reported on using the combined Census categories of White Gypsy or Irish Traveller. Therefore, in most cases, the findings do not allow for separate consideration of Irish Travellers, but there were some exceptions for example on vaccine uptake (Dixon, Mullis, and Blumenfeld 2017) or alcohol use (Hurcombe et al. 2012). In some articles Roma people are discussed alongside other Travelling people (Thompson, Stone, and Tyson 2022), although the Census 2021 has a separate category for White: Roma.

Overall, the Irish were constructed as migrants who were assumed to be white. A handful of publications called the Irish a minority ethnic group and this became more frequent from 2010. Some authors made assumptions about the legal relationship between the UK and the Republic of Ireland, indicating incomplete understanding about their history. This misunderstanding could point towards a reason why the Irish are not always included in studies of ethnic minorities in Britain.

### 4.3 Theoretical Pathways to Explain the Irish Health Disadvantage

Several articles noted that the Irish do not conform to the healthy migrant effect, which argues that the rigours of the migration process (securing funds, bureaucracy and long‐distance travel) select out the unhealthy (Bhugra et al. 2004). However, these assumptions do not hold for migrants from Ireland to Britain. In particular, proximity, low fares and lack of visa requirements mean that Irish people can make snap decisions to move to Britain (Abbotts 2004). Therefore, the Irish in Britain may include both healthy and unhealthy populations. Moreover, some people immigrated to Britain following institutionalisation in industrial schools or mother‐and‐baby homes (Leavey et al. 2007). In other words, those who are most vulnerable may have emigrated (Harding and Balarajan 2001). Another assumption of the healthy migrant effect is that later generations show similar patterns of health to the host population (Smith, Kelly, and Nazroo 2009), due to assimilation. However, studies examining second‐ and third‐generation Irish showed health inequalities persisting, disputing assimilation (Das‐Munshi et al. 2014; Harding and Balarajan 2001).

Alternative arguments focused on the value of social ties. Explaining a case study of a close‐knit Irish community in London with good mental health by drawing on the theory of salutogenesis, Malone (2001) argued that those who live near similar people have a stronger sense of coherence, better social capital and better health. Relatedly, the ethnic density effect argues that living in an area with people from your own ethnic group is advantageous to health, because of strong networks and reduced risk of racism (Bécares, Nazroo, and Stafford 2011; Das‐Munshi et al. 2010). These theories are not specific to Irish people, but have been used to understand why Irish people who live alone have high suicide rates (e.g., Leavey 1999).

A further theory was that ethnic identity is threatened by the migration process, and for the Irish this was exacerbated by moving to Britain specifically. Irish people in Britain are living in the land of their former coloniser and may face a dilemma of identity (Clarke 2010). Acculturation stress (the cognitive dissonance experienced by migrants who try to relate to their original and host country's cultures at the same time) leads to poor mental health (Aran et al. 2022; Crawford et al. 2005). The myth of return argues that some migrants who emigrate during adulthood for work assume that they will retire to their home countries but this expectation is often not met (due to practical or financial difficulties) leaving older migrants disappointed. It has been argued that the inability to return may have a stronger impact on the Irish than on emigrants from more distant countries, as it is relatively easy to return and so there may be a greater emotional reaction to the unmet expectation (Leavey, Sembhi, and Livingston 2004; Ryan et al. 2006).

Other papers focused on the effect of racism on health (Karlsen et al. 2005). Employment discrimination against Catholics was identified in Glasgow (Walls and Williams 2003, 2004). The EMPIRIC survey found that 7% of the Irish reported experiencing verbal or physical racist abuse (Karlsen et al. 2005). Socioeconomic disadvantage, influenced by structural racism, leads to poor health (Abbotts, Williams, and Ford 2001; Landman and Cruickshank 2001; Livingston et al. 2002). It is of note that the studies examining racism and discrimination experienced by the Irish were from the earlier part of the corpus; there is room for updated research in this area. Accumulation of disadvantage over the life course means that inequalities between ethnic groups become wider over time (Watkinson, Sutton, and Turner 2021). Age‐related health issues appear earlier in the life course for minority ethnic groups, a phenomenon known as weathering (Geronimus 1992; Stopforth et al. 2021). There is evidence that middle‐aged Irish in Britain exhibit health problems more typically associated with later life (Tilki et al. 2009).

The Irish experience barriers to accessing health and social care services, and cultural and structural arguments are used to explain this. For example, issues like machismo, shame, stigma and pride are said to be barriers to help seeking from health services, while a resistance to residential care is a barrier to social care (Tilki et al. 2009). In contrast, lack of recognition by services of Irish culture has been identified as a barrier (Tilki et al. 2009), while Irish Travellers experience social exclusion and difficulty accessing services (Parry et al. 2007; Van Cleemput and Parry 2001). Irish people are less likely to have knowledge about cancer symptoms or to approach services to discuss symptoms, particularly Irish men of lower socioeconomic status (Scanlon et al. 2006).

Finally, there were historic depictions of the Irish being a public health threat (Scally 2004). For example, the Irish in Liverpool were blamed for high levels of infectious diseases, without recognition of structural racism forcing migrants to live in overcrowded conditions (Scally 2004). The Irish in 19^th^ century Lancashire asylums were seen as violent, drunken and degenerate (Cox, Marland, and York 2012). Coming to more recent literature, although there is a recognition that the Irish have greater alcohol use than other groups, Tilki (2006) argued that health policy makers and practitioners may be reluctant to address alcohol use among Irish people because they are concerned about drawing on the stereotype of the Irish being problem drinkers. This could be another possible reason for excluding the Irish from studies of ethnicity and health.

### 4.4 The Evidence on the Irish Health Disadvantage

In answer to the question, ‘Are the Irish still disadvantaged in terms of health?’, the answer is: it depends on how health is measured. The objective measures (diagnosis, mortality, alcohol use and oral health) show disadvantage for the Irish while the more subjective measures (self‐rated health and limiting long‐term illness) show fewer differences. These were topics where there was a good amount of literature, so it is possible to form a synthesis. This section also discusses research on COVID‐19, as it will have relevance to health research for decades to come. The Irish Traveller group had consistently poor health over time, and so there is a separate consideration of this group. See online appendix for a summary table of the 140 papers.

### 4.5 Mortality

Since 2001, Irish people had consistently higher standardised mortality rates (SMRs) than the comparison group (general population, White British or United Kingdom (UK)‐born) from all causes. Differences were found when looking at specific causes (Irish had higher SMRs for lung and colon cancer (Wild et al. 2006), respiratory disease (Wallace and Kulu 2015), cerebrovascular disease (Wild et al. 2007), alcohol‐related disease (Bhala et al. 2009), Chronic obstructive pulmonary disease (COPD) (R. Bhopal et al. 2015), suicide (PHE 2017) and gender (men usually worse but some cases of women being worse than men). There was no difference in death from infectious disease (Wallace and Kulu 2015), hepatocellular cancer (Bhala et al. 2009) or breast cancer (Wild et al. 2006). White Irish had lower risk of death from COVID‐19 than the general population (Aldridge et al. 2020). There were consistent findings over time of higher suicide rates among the Irish, that is men and women born in the Republic of Ireland compared with those born in England & Wales (Maynard et al. 2012), Irish‐born people compared with the rate expected given their population size (Ougrin et al. 2011), and men and women born in Ireland compared with those born in England (PHE 2017).

### 4.6 Mental Health

EMPIRIC and HSE show that the Irish have high rates of common mental disorders (Sproston and Mindell 2006). The importance of subdividing by gender is demonstrated; EMPIRIC shows a worse situation for Irish men than women (Weich et al. 2004). Subdividing by socioeconomic status (SES) is also key, and greatest within‐group inequality was found for the Irish (Mangalore and Knapp 2012). For psychosis there was little difference between the Irish and White (or White British) groups (Heuvelman, Nazroo, and Rai 2018). The Irish had higher rates of diagnosis of substance use disorder (Morris et al. 2020). An early study of 1085 people aged 65+ living in Islington showed no difference in likelihood of the Irish‐born compared with the UK‐born having depression measured with the comprehensive assessment and referral evaluation (Short‐CARE) (Livingston et al. 2001), but later studies found some comparisons where White Irish do have higher levels compared with White British (Lievesley 2013 using EMPIRIC for symptoms of depression among White Irish; Mansour et al. 2020 reporting on diagnoses of White Irish within medical records in South London; Watkinson, Sutton, and Turner 2021 using the general practice patient survey of over 1 million people aged 55+ in England where White Irish people had significantly higher odds of poor quality of life in the domains of anxiety/depression compared with the White British). This could indicate depression rates are getting worse over time, or alternatively could be due to methodological differences or improvements in diagnostic accuracy over time. Rates of dementia are worsening over time. The previously mentioned Islington sample showed no difference between the Irish and UK born after controlling for demographics (Livingston et al. 2001), but 2021 data on diagnoses from South London medical records of over 12,000 people aged 65+ found the White Irish significantly more likely than the White British to have vascular dementia (Tsamakis et al. 2021), while the study of the general practice patient survey found White Irish men more likely to have a diagnosis of Alzheimer's or other dementia, and White Irish women more likely to have Alzheimer's compared with the White British/Northern Irish (Watkinson, Sutton, and Turner 2021). The White Irish do well in being offered appropriate treatment compared to other minorities (Mansour et al. 2020). The EVENS study showed the White Irish had greater odds of increased loneliness due to the pandemic and also higher risk of anxiety compared with the White British, whereas Gypsy or Travellers were less likely to experience loneliness compared with the White British (Finney et al. 2023).

### 4.7 Alcohol

The use of alcohol by the Irish is similarly high over time. Irish consistently drink more (both volume and frequency) than comparison groups (Rao, Schofield, and Ashworth 2015 using primary care records of nearly 30,000 people aged 65+ in Lambeth reporting on number of units per week, Irish compared with ‘White’; Sproston and Mindell 2006 using the Health Survey for England 2004 reporting on number of days per week drinking, number of times exceeding recommended daily limit and binge drinking, comparing the Irish‐born or second‐generation with general population), and the Irish had higher rates of alcohol‐related hospitalisations, alcoholic liver disease and deaths (Bhala et al. 2016 using the Scottish health linkage study comparing death records of the White Irish with White Scottish; PHE 2017 examining hospital admission rates in England for the White Irish). There were indications that men drink more heavily than women (Das‐Munshi et al. 2014; Hurcombe et al. 2012; McCambridge et al. 2004). The first‐generation Irish had more abstainers from alcohol (probably related to the pioneer (teetotal) pledge taken by Catholics) than the second‐generation Irish, and the second‐generation Irish drank more frequently than the White English (Smith, Kelly, and Nazroo 2009).

### 4.8 Oral Health

Oral health was only reported in some of the most recent studies in the corpus by a set of linked authors all performing secondary data analysis of the health survey for England (HSE), and seems quite poor among the Irish. Alobaidi, Bernabe, and Delgado‐Angulo (2022) using the HSE 2010/11 found that the Irish had the worst oral health of all the ethnic groups studied, examining number of teeth, self‐rated oral health and the extent to which oral health impacts daily activities. Delgado‐Angulo, Mangal, and Bernabé (2019) using a pooled analysis of six waves (1999–2005) of the HSE comparing the Irish (rather than White Irish) with White British and Amininia, Bernabe, and Delgado‐Angulo (2023) using three of the same waves of the HSE found the Irish had significantly higher odds than the White British of experiencing toothache, while Delgado‐Angulo et al. (2020) using the HSE 1999 reported that edentulousness was more common among the first‐generation Irish than among the White British.

### 4.9 Limiting Long‐Term (or Long‐Standing) Illness (LLTI)

For the White Irish or Irish, there was some indication of improvement over time, especially for women (Clucas 2009; Evandrou et al. 2016; Stopforth et al. 2021). For example, Clucas (2009) using Census 2001 Individual Licenced SARs found that the White Irish were significantly more likely to report LLTI compared with the White British in multivariate analyses; whereas Evandrou et al. (2016) used Understanding Society 2009–2011 to examine people aged 60+ and found that the Irish (not White Irish) were not significantly different from the White British in terms of limiting health conditions. Meanwhile, Stopforth et al. (2021) harmonised six datasets from 1993 to 2017 to find that the Irish (not White Irish) aged 40+ were not significantly different from the White or White British aged 40+ in reporting LLTI. When looking at particular age groups or at men separately, there are some findings of poor LLTI, but they are inconsistent (Becares 2013; Tilki et al. 2009). The White Gypsy or Irish Traveller group reported consistently high rates of LLTI over time (Irish in Britain 2023; Parry et al. 2007; Ryan et al. 2014).

### 4.10 Self‐Rated Health

Self‐rated health is measured in a number of surveys with a Likert scale question rating health from excellent to poor or very bad. There were some consistent findings over time that there is a difference at the bivariate level (the Irish worse than the British or general population) (Abbotts, Williams, and Ford 2001 comparing Catholics with non‐Catholics in Scotland in the West of Scotland Twenty‐07 study; the Irish in Britain, 2023 using the 2021 Census of England and Wales comparing the White Irish with national average) but in most cases this disappears once controlling for SES, age and sex (Evandrou et al. 2016 using Understanding Society 2009/11; Karlsen and Nazroo 2010 using the Health Survey for England 1999 and 2004; Smith, Kelly, and Nazroo 2009 also using the HSE 1999 and 2004). If the analysis looks at gender or age then more differences are found (Das‐Munshi et al. 2013 looking at second‐generation Irish in the National child development survey, finding greater differences as the cohort entered middle‐age). The White Gypsy or Irish Traveller group consistently reported bad or very bad self‐rated health over time (Irish in Britain 2023; Parry et al. 2007).

### 4.11 COVID‐19

Three sources examined COVID‐19. One looked at the risk of death in hospital and reported the White Irish (and White British) having a lower risk of death compared with the population as a whole (Aldridge et al. 2020). The second article was a letter to the editor offering potential reasons for ethnic group differences in COVID death rates, for example that the higher rate of haemochromatosis among the Irish could actually be protective, while people with sickle cell or thalassaemia (higher among Black and South Asian groups) are at higher risk (M. Cook 2021). Interestingly, only one other paper in the corpus mentioned the higher rate of haemochromatosis among the Irish but it was not the main focus of the article (Shafiq, Parveen, and Oyebode 2021). Finally, the EVENS study reported on experiences of ethnic and religious minority groups during the 2021 stage of the COVID pandemic (Finney et al. 2023). Among other outcomes, the White Irish and Gypsy or Traveller groups both had higher rates of COVID‐19 infection than the White British, and also higher rates of bereavement because of COVID‐19. This contrasts with the data from 2020 showing the White Irish had lower risk of death from COVID‐19 (Aldridge et al. 2020), indicating the White Irish were more at risk during the second wave than the first.

### 4.12 Health of Irish Travellers

Many of the findings discussed above referred to the ‘White Irish’ or ‘Irish‐born’ and did not distinguish people from Irish Traveller communities. As this latter group is known to have generally very poor health outcomes, their health status is considered separately here. As might be expected, given the small population size of Irish Travellers, most of the 13 articles with Gypsy, Roma or Irish Traveller groups as the main focus used a qualitative methodology. These articles had a starting point that this population uses health services at a lower rate, and so sought to understand health behaviours and beliefs. For example, attitudes towards breastfeeding (Condon and Salmon 2015), vaccination (Jackson et al. 2017) and alcohol use (Hurcombe et al. 2012) or understanding of cancer (Berlin, Smith, and Newton 2018). The few studies that collected quantitative data did so as part of a larger study, for example the Born in Bradford study (Pickett et al. 2022) which found that Gypsy or Traveller children were significantly more likely than White British children to lack three meals a day and more likely to worry all the time about money. An audit of GP records in the East of England found that Irish Traveller children had a 40% lower vaccination rate than non‐Travellers (Dixon, Mullis, and Blumenfeld 2017). A study of 293 Gypsies and Travellers in five regions in England found they had significantly poorer self‐rated health, were significantly more likely to have limiting long‐term illness or disability and significantly worse quality of life (EuroQol‐5D) compared with an age and sex matched comparison group, whereas the Irish Travellers were significantly more likely than UK‐born Gypsy or Travellers to be depressed or anxious (Parry et al. 2007). Finally, as mentioned above, the 2021 Census of England and Wales showed that the Gypsy or Traveller group had worse self‐rated health and more limiting long‐term illness or disability than either the White Irish or White British populations as well as much greater socioeconomic inequality (Irish in Britain 2023).

So, if the Irish are still disadvantaged in terms of health, why are they being excluded from ethnic minority booster samples, funding calls and research reports on ethnicity and health?

## 5 Discussion

I started this review assuming that interest in the health of the Irish in Britain had declined. Although 140 sources were located that examined the health of the Irish, other studies combine the White Irish group with the White British, as mentioned in the introduction. One of the research aims was to understand why the Irish are not always considered in ethnicity research. Conducting the review generated potential answers to this question.

### 5.1 Change in the View of the Irish as a Threat to British Identity

Firstly, the Irish are no longer a ‘threat’ to British identity, and are not constructed as an ‘ethnic other’ in the same way as they were historically (Walls and Williams 2003). This point reinforces the argument that ethnic identity is socially constructed and can change over time and place (Willis 2017). In the 19^th^ Century the Irish were seen as ‘pests’ and dangerous to public health (Scally 2004), due to ‘Irish fever’ (typhus) among those fleeing the famine, and also due to immigrants living in inner‐city areas rife with cholera. The Irish were historically perceived to be dirty, stupid, diseased, drunken and violent (Malone 2001). The depiction of the Irish in Punch cartoons shows them with ape‐like features, evoking a sub‐human or ‘missing link’ (Curtis Jr 1971; Punch 1862). However, the discourse about the Irish changed in the 20th century, coinciding with the forming of the Irish Free State and the later Republic of Ireland, and also immigration from the New Commonwealth. A Cabinet Office memorandum stated ‘*The Irish are not*—*whether they like it or not*—*a different race from the ordinary inhabitants of Great Britain*’ (Cornish 1955, 2–3), while at the same time the memorandum depicted the Irish as “less civilised than the English” (M. J. Hickman 1998: 297). M. J. Hickman (1998: 290) argued that there was an assumption of “cultural homogeneity” across the British Isles, a myth that challenges recognition of the ethnic distinctiveness of the White Irish people in Britain (Willis 2017). The whiteness of most Irish immigrants became conflated with Irishness and was contrasted with the immigrants of colour from the Caribbean, South Asia and East Africa. The inequalities and discrimination experienced by New Commonwealth immigrants were recognised and became important policy issues. Meanwhile the Irish, despite also experiencing discrimination, were not as high up the public agenda. In summary, the Irish had been viewed as subhuman violent disease carriers, with a foreign religion, disposed to political disruption and terrorism. They therefore represented a threat to Britain, and something that needed a policy response (Scally 2004). However, post‐independence (and the Good Friday Agreement) the political question subsided.

### 5.2 Republic of Ireland Not Included in Immigration Restrictions

Secondly, immigration law in Britain tightened restrictions on citizens of the New Commonwealth (Spencer 1997) but the Republic of Ireland was no longer part of the Commonwealth (M. J. Hickman 1998). The Irish were omitted from immigration discussions because, under the Common Travel Area, there were no restrictions, and this may have led to the Irish being left out of research that focused on immigrants. Furthermore, M. J. Hickman (1998) argues that it was in the interests of the British government to not impose restrictions on Irish citizens as they were an important part of the labour force in post‐war Britain, and doing so would have required monitoring also of the movements of Northern Irish people, which was considered politically and logistically impossible. Similarly, arguments about restricting freedom of movement to EU citizens during the Brexit debate did not include the Irish. There may have been an assumption that the Common Travel Area and rights to live and work would persist (although this was not guaranteed), but there may also have been an underlying sense in British society that Ireland is not ‘different enough’ from Britain, and (at the extreme) a misunderstanding about the legal relationship between them.

### 5.3 Equating Irishness With Whiteness

Thirdly, there is an assumption in the literature that all Irish people are White. As discourses about ethnicity and immigration to Britain have largely focused on people of colour, the Irish tend to be omitted (Foster 2003; Walls and Williams 2003). However, one does not have to be a person of colour to be an ethnic minority in Britain. It is common to see the terms ‘ethnic minority’ or ‘ethnic’ used to refer only to people of colour. This demonstrates a misconception that may be part of the reason why the Irish are left out of some ethnicity research. Relatedly, it may be assumed that the White Irish do not experience racial discrimination, but several of the studies reported on racism, particularly against those who lived through the eras of ‘no blacks, no dogs, no Irish’ and the Troubles (Fitzpatrick and Newton 2005). Here, we can draw on the theory of accumulation of disadvantage over the life course and the link to ‘weathering’ of health status among minority groups, which can be used to explain poor health outcomes for Irish people in later life.

### 5.4 Assimilation Hypothesis

Fourthly, as the Irish population in Britain has been in residence for a long time, it could be assumed that they have assimilated and health differences have disappeared. The evidence shows that this is not true. On the contrary, the continued disadvantages in terms of common mental disorder and psychological symptoms (Das‐Munshi et al. 2013, 2014) and mortality (Harding and Balarajan 2001) into the second‐ and third‐generations are in opposition to the assimilation hypothesis.

### 5.5 Change in Government Interest in Diversity

Fifthly, J. Cook (2010) argues that there was a UK policy shift away from interest in funding minority ethnic community groups and that this may have been a reaction to the July 7th bombings in London. Segregated communities came to be seen negatively by local authorities, and the preference was for integration. Thus, a survey focussing on identity (Citizenship Survey) was cancelled in 2011. The later ‘hostile environment’ migration policy perhaps did not view the Irish as ‘undesirable’ because they are incorrectly seen as ethnically similar to White British people. This could be why some of the articles combined the UK‐born with people born in the Republic of Ireland (Bansal et al. 2014; R. S. Bhopal et al. 2018; Cézard et al. 2020).

### 5.6 Assumption About Lack of Barriers

Sixthly, as the Irish are mostly native speakers of English, and entitled to use the NHS, they may be assumed to have no challenges accessing services. However, findings from Moselhy and Telfer (2002) show important difficulties in accessing addiction services, in that the Irish patients were illiterate and learnt about the service through word‐of‐mouth. Furthermore, Tilki (2017) identifies ways health and social care services need to be more culturally appropriate for Irish people with dementia.

### 5.7 Assumption About Affluence

Seventhly, the White Irish do not all have low SES, which is one of the key drivers of poor health. Instead, there are two distributions of SES among the White Irish in Britain: one well‐educated professional class and another less‐educated manual class (Irish in Britain 2023; Tilki et al. 2009). It is the latter group who have greater health needs but are ignored when interactions between SES and ethnicity are not considered. In contrast, the Irish Traveller group have poorer SES than the White Irish. For example, the Gypsy or Irish Traveller group have a much lower percentage of individuals in the managerial/professional category of employment (15% female, 17% male) compared with the White Irish (50% female, 50% male) (Irish in Britain 2023). Similarly, the Gypsy or Irish Traveller group has a higher percentage of economically inactive people (53%) compared with the White Irish (21%) or general population (25%). These findings are among the reasons why Gypsy, Roma and Traveller communities have been included as a priority in the NHS Core20PLUS5 strategy.

In summary, there may be an underlying view in British society that people from the Republic of Ireland are more similar than different, due to proximity, colonial history, common language and whiteness (Bhugra et al. 2004; Tilki et al. 2010). There could be an incorrect view that the Republic of Ireland still belongs to Britain and therefore its immigrants do not need to be monitored. These reasons combine to leave Irish immigrants and their descendants off the agenda when examining ethnic health inequalities.

### 5.8 Limitations and Strengths

This article may be limited by the choice of databases, as key articles may have been missed. However, the additional strategies of using Connected Papers, snowballing and drawing on already known articles helped to overcome this problem. The state‐of‐the‐art methodology may be criticised for being subjective, but the interpretivist philosophical position was appropriate for the aims of this paper. The literature search process was conducted robustly and rigorously, so is not weaker than the process for a systematic review. Most of the articles were quantitative, but the qualitative articles provided important theoretical explanations for the quantitative findings. This paper represents a key contribution to the sociology of health, as it synthesises a large corpus of evidence on the health of Irish people, addresses theoretical relationships between health and ethnicity and brings to prominence an overlooked population group.

## 6 Conclusion

There is a decline over time but interest in the health of the Irish in Britain has not disappeared. The Irish are still disadvantaged in key measures of health (e.g., mortality, suicide and alcohol use), and may be worsening in others (e.g., dementia and oral health). The White Irish in Britain are the oldest population in the country, and some have experienced lifetime trauma related to institutional abuse in Ireland. This population is at particular risk of reexperiencing trauma if they require residential care in later life, especially if they also have dementia. Older Irish people of colour may have experienced a lifetime of prejudice, linking to health problems. Irish Travellers are a younger population with high social and health disadvantages. Policy makers and health and social care providers should recognise that the Irish in Britain are a minority ethnic group with specific histories and traditions that need to be adjusted for. Failure to recognise the distinctiveness of the Irish in Britain can lead to failure to address the serious disadvantages experienced by some in this group, particularly among the less affluent. Surveys should include the Irish in their samples in large enough numbers to allow statistical comparison, and recognise that not all Irish people are white. Researchers should report on these groups in their findings where data are available, cross‐referencing with gender and SES. Funders interested in ethnic inequalities must not exclude the White Irish from their calls, as doing so is based on false assumptions.

This review has demonstrated the importance of including Irish people in Britain in health research. More work is needed to explore evidence for the theoretical pathways to ill‐health, which will point towards ways to intervene and improve outcomes. This in turn needs to be monitored by including the Irish in routinely collected data.

## Author Contributions

**Rosalind Willis:** conceptualization (lead), formal analysis (lead), investigation (lead), methodology (lead), visualization (lead), writing–original draft (lead), writing–review and editing (lead).

## Ethics Statement

The author has nothing to report.

## Conflicts of Interest

The author declares no conflicts of interest.

## Supporting information

Supporting Information S1

Supporting Information S2

Supporting Information S3

## Data Availability

Supplementary online material is available and has been submitted as part of the documents for review with this article. This comprises: Appendix 1: List of the 140 sources included in the review. Appendix 2: Summary table of the 140 sources included in the review. Appendix 3: Literature search strategy tables.
