# Supplementary material for: Where Are the Irish in Research on Ethnic Health Inequalities in Britain? A State‐Of‐The‐Art Literature Review
Source: Sociol Health Illn. 2025 Jan 2;47(1):e13874. doi: 10.1111/1467-9566.13874 (PMC11694090; doi:10.1111/1467-9566.13874)
Supplement: Supplementary file 1 — Supporting Information S1 [file SHIL-47-0-s001.docx]

**Online Appendix: Full list of 140 references included in the state-of-the-art review**

Abbotts, J. (2004) 'Irish Catholic health disadvantage in the West of Scotland', *Scottish Affairs*, 46(1), pp. 131-148.

Abbotts, J., Harding, S. and Cruickshank, K. (2004) 'Cardiovascular risk profiles in UK-born Caribbeans and Irish living in England and Wales', *Atherosclerosis*, 175(2), pp. 295-303.

Abbotts, J., Williams, R. and Ford, G. (2001) 'Morbidity and Irish Catholic descent in Britain. Relating health disadvantage to socio-economic position', *Social science & medicine (1982)*, 52(7), pp. 999-1005.

Aldridge, R.W. *et al.* (2020) 'Black, Asian and Minority Ethnic groups in England are at increased risk of death from COVID-19: indirect standardisation of NHS mortality data', *Wellcome open research*, 5, p. 88.

Allnock, D. *et al.* (2012) 'In demand: Therapeutic services for children and young people who have experienced sexual abuse', *Child Abuse Review*, 21(5), pp. 318-334.

Alobaidi, F., Bernabe, E. and Delgado-Angulo, E.K. (2022) 'The Role of Area Deprivation in Explaining Ethnic Inequalities in Adult Oral Health in England', *Community dental health*, 39(3), pp. 158-164.

Amininia, M., Bernabe, E. and Delgado-Angulo, E.K. (2023) 'Ethnicity, Social Support and Oral Health Among English Individuals', *Community dental health*.

Anseimi, L. *et al.* (2020) 'Estimating local need for mental healthcare to inform fair resource allocation in the NHS in England: cross-sectional analysis of national administrative data linked at person level', *British Journal of Psychiatry*, 216(6), pp. 338-344.

Aspinall, P.J. (2002) 'Suicide amongst Irish migrants in Britain: A review of the identity and integration hypothesis', *International Journal of Social Psychiatry*, 48(4), pp. 290-304.

Aspinall, P.J. and Jacobsen, B. (2004) 'Ethnic disparities in health and health care: A focused review of the evidence and selected examples of good practice'. London: London Health Observatory.

Aspinall, P.J. and Mitton, L. (2014) 'Smoking prevalence and the changing risk profiles in the UK ethnic and migrant minority populations: implications for stop smoking services', *Public health*, 128(3), pp. 297-306.

Baker, J. *et al.* (2013) 'Ethnic differences in the cost-effectiveness of targeted and mass screening for high cardiovascular risk in the UK: cross-sectional study', *Heart (British Cardiac Society)*, 99(23), pp. 1766-1771.

Bansal, N. *et al.* (2014) 'Ethnicity and first birth: age, smoking, delivery, gestation, weight and feeding: Scottish health and ethnicity linkage study', *European Journal of Public Health*, 24(6), pp. 911-916.

Barry, E. *et al.* (2015) 'Ethnic group variations in alcohol-related hospital admissions in England: does place matter?', *Ethnicity & health*, 20(6), pp. 557-563.

Becares, L. (2013) *Which ethnic groups have the poorest health? Ethnic health inequalities 1991 to 2011. Dynamics of Diversity: Evidence from the 2011 Census*. Manchester.

Berlin, J., Smith, D. and Newton, P. (2018) '"It's because it's cancer, not because you're a Traveller"-exploring lay understanding of cancer in English Romany Gypsy and Irish Traveller communities', *European Journal of Oncology Nursing*, 34, pp. 49-54.

Bhala, N. *et al.* (2009) 'Alcohol-related and hepatocellular cancer deaths by country of birth in England and Wales: analysis of mortality and census data', *Journal of Public Health*, 31(2), pp. 250-257.

Bhala, N. *et al.* (2016) 'Ethnic Variations in Liver- and Alcohol-Related Disease Hospitalisations and Mortality: The Scottish Health and Ethnicity Linkage Study', *Alcohol and alcoholism (Oxford, Oxfordshire)*, 51(5), pp. 593-601.

Bhavsar, V. and Bhugra, D. (2009) 'Bethlem's Irish: migration and distress in nineteenth-century London', *History of psychiatry*, 20(78 Pt 2), pp. 184-198.

Bhopal, R. *et al.* (2015) 'Risk of respiratory hospitalization and death, readmission and subsequent mortality: scottish health and ethnicity linkage study', *European journal of public health*, 25(5), pp. 769-774.

Bhopal, R.S. *et al.* (2012) 'Does the 'Scottish effect' apply to all ethnic groups? All-cancer, lung, colorectal, breast and prostate cancer in the Scottish Health and Ethnicity Linkage Cohort Study', *Bmj Open*, 2(5).

Bhopal, R.S. *et al.* (2014) 'Ethnic variations in five lower gastrointestinal diseases: Scottish Health and Ethnicity Linkage Study', *Bmj Open*, 4(10).

Bhopal, R.S. *et al.* (2018) 'Mortality, ethnicity, and country of birth on a national scale, 2001-2013: A retrospective cohort (Scottish Health and Ethnicity Linkage Study)', *Plos Medicine*, 15(3).

Bhopal, R.S., Humphry, R.W. and Fischbacher, C.M. (2013) 'Changes in cardiovascular risk factors in relation to increasing ethnic inequalities in cardiovascular mortality: comparison of cross-sectional data in the Health Surveys for England 1999 and 2004', *BMJ open*, 3(9), p. e003485.

Bhugra, D. *et al.* (2004) 'Migration and mental health', *Acta Psychiatrica Scandinavica*, 109(4), pp. 243-258.

Bhui, K. *et al.* (2005) 'Racial/ethnic discrimination and common mental disorders among workers: findings from the EMPIRIC Study of Ethnic Minority Groups in the United Kingdom', *American journal of public health*, 95(3), pp. 496-501.

Bracken, P.J. and O'Sullivan, P. (2001) 'The invisibility of Irish migrants in British health research', *Irish Studies Review*, 9(1), pp. 41-51.

Cézard, G. *et al.* (2020) 'Ethnic variations in falls and road traffic injuries resulting in hospitalisation or death in Scotland: the Scottish Health and Ethnicity Linkage Study', *Public health*, 182, pp. 32-38.

Chum, A., Teo, C. and Azra, K.K. (2022) 'Does the longitudinal association between neighbourhood cohesion and mental health differ by ethnicity? Results from the UK Household Longitudinal Survey', *Social psychiatry and psychiatric epidemiology*, 57(4), pp. 859-872.

Clarke, L. (2010) 'An examination of the mental health of Irish migrants to England using a concept of Diaspora', *Advances in Mental Health*, 9(3), pp. 231-242.

Clucas, M. (2009) 'The Irish health disadvantage in England: contribution of structure and identity components of Irish ethnicity', *Ethnicity & Health*, 14(6), pp. 553-573.

Co, M. *et al.* (2023) 'Ethnicity and survival after a dementia diagnosis: a retrospective cohort study using electronic health record data', *Alzheimers Research & Therapy*, 15(1).

Cohuet, S. *et al.* (2009) 'A measles outbreak in the Irish traveller ethnic group after attending a funeral in England, March-June 2007', *Epidemiology and Infection*, 137(12), pp. 1759-1765.

Collingwood Bakeo, A. (2006) 'Investigating variations in infant mortality in England and Wales by mother's country of birth, 1983-2001', *Paediatric and Perinatal Epidemiology*, 20(2), pp. 127-139.

Commander, M.J. *et al.* (2003) 'Characteristics of patients and patterns of psychiatric service use in ethnic minorities', *The International journal of social psychiatry*, 49(3), pp. 216-224.

Condon, L.J. and Salmon, D. (2015) ''You likes your way, we got our own way': Gypsies and Travellers' views on infant feeding and health professional support', *Health expectations : an international journal of public participation in health care and health policy*, 18(5), pp. 784-795.

Cook, J. (2010) 'Exploring older women's citizenship: understanding the impact of migration in later life', *Ageing & Society*, 30, pp. 253-273.

Cook, M. (2021) 'Potential factors linked to high COVID-19 death rates in British minority ethnic groups', *The Lancet. Infectious diseases*, 21(4), p. e68.

Cox, C., Marland, H. and York, S. (2012) 'Emaciated, Exhausted, and Excited: The Bodies and Minds of the Irish in Late Nineteenth-Century Lancashire Asylums', *Journal of Social History*, 46(2), pp. 500-524.

Crawford, M.J. *et al.* (2005) 'Suicidal ideation and suicide attempts among ethnic minority groups in England: results of a national household survey', *Psychological medicine*, 35(9), pp. 1369-1377.

Curran, M.J., Bunting, B. and MacLachlan, M. (2002) 'The health and acculturation of the Irish Diaspora in Britain', *The Irish Journal of Psychology*, 23(3-4), pp. 222-233.

Das-Munshi, J. *et al.* (2016) 'Ethnicity and cardiovascular health inequalities in people with severe mental illnesses: protocol for the E-CHASM study', *Social psychiatry and psychiatric epidemiology*, 51(4), pp. 627-638.

Das-Munshi, J. *et al.* (2010) 'Understanding the effect of ethnic density on mental health: multi-level investigation of survey data from England', *BMJ (Clinical research ed.)*, 341, p. c5367.

Das‐Munshi, J. *et al.* (2014) 'Cross‐cultural factorial validation of the Clinical Interview Schedule-Revised (CIS‐R); Findings from a nationally representative survey (EMPIRIC)', *International Journal of Methods in Psychiatric Research*, 23(2), pp. 229-244.

Das-Munshi, J. *et al.* (2019) 'Depression and cause-specific mortality in an ethnically diverse cohort from the UK: 8-year prospective study', *Psychological Medicine*, 49(10), pp. 1639-1651.

Das-Munshi, J. *et al.* (2017) 'Ethnicity and excess mortality in severe mental illness: a cohort study', *The lancet. Psychiatry*, 4(5), pp. 389-399.

Das-Munshi, J. *et al.* (2013) 'Does childhood adversity account for poorer mental and physical health in second-generation Irish people living in Britain? Birth cohort study from Britain (NCDS)', *BMJ open*, 3(3).

Das-Munshi, J. *et al.* (2014) 'Born into adversity: psychological distress in two birth cohorts of second-generation Irish children growing up in Britain', *Journal of public health (Oxford, England)*, 36(1), pp. 92-103.

Das-Munshi, J. *et al.* (2014) 'Does social disadvantage over the life-course account for alcohol and tobacco use in Irish people? Birth cohort study', *European journal of public health*, 24(4), pp. 594-599.

Delaney, L., Fernihough, A. and Smith, J.P. (2013) 'Exporting Poor Health: The Irish in England', *Demography*, 50(6), pp. 2013-2035.

Delgado-Angulo, E.K., Mangal, M. and Bernabé, E. (2019) 'Socioeconomic inequalities in adult oral health across different ethnic groups in England', *Health and quality of life outcomes*, 17(1), p. 85.

Delgado-Angulo, E.K. *et al.* (2020) 'Is there a healthy migrant effect in relation to oral health among adults in England?', *Public health*, 181, pp. 53-58.

Dixon, K.C., Mullis, R. and Blumenfeld, T. (2017) 'Vaccine uptake in the Irish Travelling community: an audit of general practice records', *Journal of Public Health*, 39(4), pp. E235-E241.

Emerson, E. (2012) 'Deprivation, ethnicity and the prevalence of intellectual and developmental disabilities', *Journal of Epidemiology and Community Health*, 66(3), pp. 218-224.

Evandrou, M. *et al.* (2016) 'Ethnic inequalities in limiting health and self-reported health in later life revisited', *Journal of Epidemiology and Community Health*, 70(7), pp. 653-662.

Finney, N. *et al.* (Eds.) (2023) *Racism and Ethnic Inequality in a Time of Crisis: Findings from the Evidence for Equality National Survey.* Bristol: Policy Press.

Fischbacher, C.M. *et al.* (2007) 'Variations in all cause and cardiovascular mortality by country of birth in Scotland, 1997-2003', *Scottish medical journal*, 52(4), pp. 5-10.

Fitzpatrick, M. and Newton, J. (2005) 'Profiling mental health needs: what about your Irish patients?', *British Journal of General Practice*, 55(519), pp. 739-740.

Foster, J.H. (2003) 'The Irish alcohol misuser in England: Ill served by research and policy? Some suggestions for future research opportunities', *Drugs: Education, Prevention & Policy*, 10(1), pp. 57-63.

Gruer, L. *et al.* (2016) 'Life expectancy of different ethnic groups using death records linked to population census data for 4.62 million people in Scotland', *Journal of epidemiology and community health*, 70(12), pp. 1251-1254.

Gruer, L.D. *et al.* (2018) 'Differences in all-cause hospitalisation by ethnic group: a data linkage cohort study of 4.62 million people in Scotland, 2001-2013', *Public Health*, 161, pp. 5-11.

Harding, S. and Balarajan, R. (2001) 'Mortality of third generation Irish people living in England and Wales: longitudinal study', *BMJ (Clinical research ed.)*, 322(7284), pp. 466-467.

Harding, S., Rosato, M. and Teyhan, A. (2008) 'Trends for coronary heart disease and stroke mortality among migrants in England and Wales, 1979-2003: slow declines notable for some groups', *Heart*, 94(4), pp. 463-470.

Harding, S., Rosato, M. and Teyhan, A. (2009) 'Trends in cancer mortality among migrants in England and Wales, 1979-2003', *European journal of cancer (Oxford, England : 1990)*, 45(12), pp. 2168-2179.

Heuvelman, H., Nazroo, J. and Rai, D. (2018) 'Investigating ethnic variations in reporting of psychotic symptoms: a multiple-group confirmatory factor analysis of the Psychosis Screening Questionnaire', *Psychological medicine*, 48(16), pp. 2757-2765.

Hurcombe, R. *et al.* (2012) 'Perspectives on alcohol use in a traveller community: An exploratory case study', *Ethnicity and Inequalities in Health and Social Care*, 5(3), pp. 89-97.

Irish in Britain (2023) 'Preliminary findings from the 2021 Census of England and Wales. First Summary Report - 6th April 2023.'. London: Irish in Britain. Available at: https://www.irishinbritain.org/assets/files/Irish-in-Britain-summary-report---April-2023.pdf (Accessed: 26/4/2023).

Jack, R.H. *et al.* (2014) 'Breast cancer screening uptake among women from different ethnic groups in London: a population-based cohort study', *BMJ open*, 4(10), p. e005586.

Jackson, C. *et al.* (2017) 'Needles, Jabs and Jags: a qualitative exploration of barriers and facilitators to child and adult immunisation uptake among Gypsies, Travellers and Roma', *BMC public health*, 17(1), p. 254.

Jackson, C. *et al.* (2016) 'UNderstanding uptake of Immunisations in TravellIng aNd Gypsy communities (UNITING): a qualitative interview study', *Health technology assessment (Winchester, England)*, 20(72), pp. 1-176.

Kapadia, D., Nazroo, J. and Tranmer, M. (2018) 'Ethnic differences in women's use of mental health services: do social networks play a role? Findings from a national survey', *Ethnicity & health*, 23(3), pp. 293-306.

Karlsen, S. and Nazroo, J. (2010) 'Religious and ethnic differences in health: Evidence from the Health Surveys for England 1999 and 2004', *Ethnicity & Health*, 15(6), pp. 549-568.

Karlsen, S. *et al.* (2005) 'Racism, psychosis and common mental disorder among ethnic minority groups in England', *Psychological medicine*, 35(12), pp. 1795-1803.

Kelly, A. and Ciclitira, K. (2011) 'Eating and drinking habits of young London-based Irish men: a qualitative study', *Journal of Gender Studies*, 20(3), pp. 223-235.

Koffman, J. *et al.* (2014) 'Does Ethnicity Affect Where People with Cancer Die ? A Population-Based 10 Year Study', *Plos One*, 9(4).

Landman, J. and Cruickshank, J.K. (2001) 'A review of ethnicity, health and nutrition-related diseases in relation to migration in the United Kingdom', *Public health nutrition*, 4(2B), pp. 647-657.

Leavey, G. and Eliacin, J. (2013) ''The past is a foreign country': vulnerability to mental illness among return migrants', in Percival, J. (ed.) *Return Migration in Later Life: International Perspectives*. Bristol: Bristol University Press, pp. 195-218.

Leavey, G. *et al.* (2007) 'Explanations of depression among Irish migrants in Britain', *Social science & medicine (1982)*, 65(2), pp. 231-244.

Leavey, G., Sembhi, S. and Livingston, G. (2004) 'Older Irish migrants living in London: Identity, loss and return', *Journal of Ethnic and Migration Studies*, 30(4), pp. 763-779.

Lievesley, N. (2013) *Evidence to the Birmingham Policy Commission: Healthy Ageing in the 21st Century*. London.

Livingston, G. *et al.* (2001) 'Mental health of migrant elders: The Islington study', *The British Journal of Psychiatry*, 179(4), pp. 361-366.

Livingston, G. *et al.* (2002) 'Accessibility of health and social services to immigrant elders: The Islington Study', *British Journal of Psychiatry*, 180, pp. 369-373.

Lowe, M. *et al.* (2020) 'Human leukocyte antigen associations with renal function among ethnic minorities in the United Kingdom', *HLA*, 96(6), pp. 697-708.

Malone, M. (2001) 'The health experience of Irish people in a North West London 'community saved'', *Community, Work & Family*, 4(2), pp. 195-213.

Mangalore, R. and Knapp, M. (2012) 'Income-related inequalities in common mental disorders among ethnic minorities in England', *Social psychiatry and psychiatric epidemiology*, 47(3), pp. 351-359.

Mansour, R. *et al.* (2020) 'Late-life depression in people from ethnic minority backgrounds: Differences in presentation and management', *Journal of affective disorders*, 264, pp. 340-347.

Maynard, M.J. *et al.* (2012) 'Trends in suicide among migrants in England and Wales 1979–2003', *Ethnicity & Health*, 17(1-2), pp. 135-140.

McCambridge, J. *et al.* (2004) 'Patterns of alcohol consumption and problems among the Irish in London: A preliminary comparison of pub drinkers in London and Dublin', *Addiction Research & Theory*, 12(4), pp. 373-384.

Millard, A.D. *et al.* (2015) 'Mortality differences and inequalities within and between 'protected characteristics' groups, in a Scottish Cohort 1991-2009', *International Journal for Equity in Health*, 14.

Millett, C. *et al.* (2007) 'Socio-economic status, ethnicity and diabetes management: an analysis of time trends using the health survey for England', *Journal of public health (Oxford, England)*, 29(4), pp. 413-419.

Mindell, J.S. *et al.* (2014) 'Explanatory factors for health inequalities across different ethnic and gender groups: Data from a national survey in England', *Journal of Epidemiology and Community Health*, 68(12), pp. 1133-1144.

Moore, J. (2019) 'Perceived functional social support and self-rated health: The health promoting effects of instrumental support for the Irish community in London', *Journal of Immigrant and Minority Health*, 21(5), pp. 1004-1011.

Moore, J. (2022) 'Correlates of primary healthcare access and the moderating effects of gender: A cross sectional analysis of Irish migrants', *Journal of Immigrant and Minority Health*, 24(2), pp. 546-550.

Moore, J., Flynn, M. and Morgan, M. (2019) 'Social ecological resilience and mental wellbeing of Irish emigrant survivors of clerical institutional childhood abuse', *Child Abuse Review*, 28(1), pp. 52-68.

Moore, J., Thornton, C. and Hughes, M. (2017) 'On the road to resilience: The help‐seeking experiences of Irish emigrant survivors of institutional abuse', *Child Abuse Review*, 26(5), pp. 375-387.

Moore, J. *et al.* (2018) 'The moderating effect of functional social support on the association between unfair treatment and self-rated health: A study of the resilience of a community-based sample of Irish migrants in London', *Irish Journal of Sociology*, 26, pp. 267 - 288.

Morris, R.M. *et al.* (2020) 'Ethnicity and impact on the receipt of cognitive-behavioural therapy in people with psychosis or bipolar disorder: an English cohort study', *BMJ open*, 10(12), p. e034913.

Moselhy, H.F. and Telfer, I. (2002) 'The pattern of substance misuse among ethnic minorities in a community drug setting', *The European Journal of Psychiatry*, 16(4), pp. 240-247.

Niksic, M. *et al.* (2016) 'Ethnic differences in cancer symptom awareness and barriers to seeking medical help in England', *British journal of cancer*, 115(1), pp. 136-144.

Nilforooshan, R., Amin, R. and Warner, J. (2009) 'Ethnicity and outcome of appeal after detention under the Mental Health Act 1983', *Psychiatric Bulletin*, 33(8), pp. 288-290.

Ougrin, D. *et al.* (2011) 'Suicide survey in a London borough: primary care and public health perspectives', *Journal of Public Health*, 33(3), pp. 385-391.

Parry, G. *et al.* (2007) 'Health status of Gypsies and Travellers in England', *Journal of Epidemiology and Community Health*, 61(3), pp. 198-204.

PHE (2017) 'Public Health Outcomes Framework: Health Equity Report. Focus on Ethnicity'. London: Public Health England. Available at: https://www.gov.uk/government/uploads/system/uploads/attachment_data/file/629563/PHOF_Health_Equity_Report.pdf.

Pickett, K.E. *et al.* (2022) 'Vulnerabilities in child well-being among primary school children: a cross-sectional study in Bradford, UK', *Bmj Open*, 12(6).

Polling, C. *et al.* (2021) 'Variation in rates of self-harm hospital admission and re-admission by ethnicity in London: a population cohort study', *Social psychiatry and psychiatric epidemiology*, 56(11), pp. 1967-1977.

Puthussery, S. *et al.* (2008) ''They're more like ordinary stroppy British women': attitudes and expectations of maternity care professionals to UK-born ethnic minority women', *Journal of Health Services Research & Policy*, 13(4), pp. 195-201.

Puthussery, S. *et al.* (2010) ''You need that loving tender care': maternity care experiences and expectations of ethnic minority women born in the United Kingdom', *Journal of health services research & policy*, 15(3), pp. 156-162.

Rao, R., Schofield, P. and Ashworth, M. (2015) 'Alcohol use, socioeconomic deprivation and ethnicity in older people', *BMJ open*, 5(8), p. e007525.

Rao, R., Wolff, K. and Marshall, E.J. (2008) 'Alcohol use and misuse in older people: A local prevalence study comparing English and Irish inner-city residents living in the UK', *Journal of Substance Use*, 13(1), pp. 17-26.

Roman-Urrestarazu, A. *et al.* (2021) 'Association of Race/Ethnicity and Social Disadvantage With Autism Prevalence in 7 Million School Children in England', *Jama Pediatrics*, 175(6), p. 11.

Ryan, L. *et al.* (2014) 'Analysis of 2011 Census data. Irish community statistics, England and selected urban areas. Report for London'. London: Social Policy Research Centre, Middlesex University.

Ryan, L. *et al.* (2006) 'Depression in Irish migrants living in London: case-control study', *The British journal of psychiatry : the journal of mental science*, 188, pp. 560-566.

Saxena, S., Eliahoo, J. and Majeed, A. (2002) 'Socioeconomic and ethnic group differences in self reported health status and use of health services by children and young people in England: cross sectional study', *BMJ (Clinical research ed.)*, 325(7363), p. 520.

Scally, G. (2004) ''The very pests of society': the Irish and 150 years of public health in England', *Clinical medicine (London, England)*, 4(1), pp. 77-81.

Scanlon, K. *et al.* (2006) 'Potential barriers to prevention of cancers and to early cancer detection among Irish people living in Britain: A qualitative study', *Ethnicity & Health*, 11(3), pp. 325-341.

Shafiq, S., Parveen, S. and Oyebode, J.R. (2021) 'How people of African Caribbean or Irish ethnicity cope with long-term health conditions in UK community settings: A systematic review of qualitative, quantitative and mixed method studies', *Health & social care in the community*, 29(2), pp. 319-327.

Sharpe, K.H. *et al.* (2015) 'Policy for home or hospice as the preferred place of death from cancer: Scottish Health and Ethnicity Linkage Study population cohort shows challenges across all ethnic groups in Scotland', *BMJ supportive & palliative care*, 5(4), pp. 443-451.

Smith, N.R. and Grundy, E. (2011) 'Time period trends in ethnic inequalities in limiting long term illness in England and Wales', *Ethnicity and Inequalities in Health and Social Care*, 4(4), pp. 200-209.

Smith, N.R., Kelly, Y.J. and Nazroo, J.Y. (2009) 'Intergenerational continuities of ethnic inequalities in general health in England', *Journal of Epidemiology and Community Health*, 63(3), pp. 253-258.

Smith, N.R., Kelly, Y.J. and Nazroo, J.Y. (2012) 'The effects of acculturation on obesity rates in ethnic minorities in England: evidence from the Health Survey for England', *European journal of public health*, 22(4), pp. 508-513.

Sproston, K. and Mindell, J. (2006) *Health Survey for England 2004 The Health of Minority Ethnic Groups: Summary of Key Findings*. London.

Stopforth, S. *et al.* (2021) 'Ethnic inequalities in health in later life, 1993–2017: the persistence of health disadvantage over more than two decades', *Ageing & Society*, FirstView, pp. 1-29.

Thompson, R.M., Stone, B.V. and Tyson, P.J. (2022) 'Mental health support needs within Gypsy, Roma, and Traveller communities: a qualitative study', *Mental Health and Social Inclusion*, 26(2), pp. 144-155.

Tilki, M. (2006) 'The social contexts of drinking among Irish men in London', *Drugs-Education Prevention and Policy*, 13(3), pp. 247-261.

Tilki, M. (2017) 'Forgotten but not gone: Older Irish with dementia in England', *Journal of Dementia Care*, 25(3), pp. 30-31.

Tilki, M. *et al.* (2010) 'Older Irish people with dementia in England', *Advances in Mental Health*, 9(3), pp. 219-230.

Tilki, M. *et al.* (2009) 'The forgotten Irish: Report of a research project commissioned by Ireland Fund of Great Britain'. London: Social Policy Research Centre, Middlesex University.

Tsamakis, K. *et al.* (2021) 'Dementia in People from Ethnic Minority Backgrounds: Disability, Functioning, and Pharmacotherapy at the Time of Diagnosis', *Journal of the American Medical Directors Association*, 22(2), pp. 446-452.

Twamley, K. *et al.* (2011) 'UK-born ethnic minority women and their experiences of feeding their newborn infant', *Midwifery*, 27(5), pp. 595-602.

Van Cleemput, P. and Parry, G. (2001) 'Health status of Gypsy travellers', *Journal of Public Health Medicine*, 23(2), pp. 129-134.

Van Hout, M.-C. and Staniewicz, T. (2012) 'Roma and Irish Traveller housing and health—A public health concern', *Critical Public Health*, 22(2), pp. 193-207.

Wallace, M. and Kulu, H. (2015) 'Mortality among immigrants in England and Wales by major causes of death, 1971-2012: A longitudinal analysis of register-based data', *Social science & medicine (1982)*, 147, pp. 209-221.

Walls, P. and Williams, R. (2003) 'Sectarianism at work: Accounts of employment discrimination against Irish Catholics in Scotland', *Ethnic and Racial Studies*, 26(4), pp. 632-661.

Walls, P. and Williams, R. (2004) 'Accounting for Irish Catholic ill health in Scotland: a qualitative exploration of some links between 'religion', class and health', *Sociology of health & illness*, 26(5), pp. 527-556.

Watkinson, R.E., Sutton, M. and Turner, A.J. (2021) 'Ethnic inequalities in health-related quality of life among older adults in England: secondary analysis of a national cross-sectional survey', *The Lancet. Public health*, 6(3), pp. e145-e154.

Weich, S. *et al.* (2004) 'Common mental disorders and ethnicity in England: the EMPIRIC study', *Psychological medicine*, 34(8), pp. 1543-1551.

Wild, S.H. *et al.* (2007) 'Mortality from all causes and circulatory disease by country of birth in England and Wales 2001-2003', *Journal of public health (Oxford, England)*, 29(2), pp. 191-198.

Wild, S.H. *et al.* (2006) 'Mortality from all cancers and lung, colorectal, breast and prostate cancer by country of birth in England and Wales, 2001-2003', *British Journal of Cancer*, 94(7), pp. 1079-1085.

Wohland, P. *et al.* (2015) 'Inequalities in healthy life expectancy between ethnic groups in England and Wales in 2001', *Ethnicity & Health*, 20(4), pp. 341-353.

Wright, D.M. *et al.* (2017) 'Does equality legislation reduce intergroup differences? Religious affiliation, socio-economic status and mortality in Scotland and Northern Ireland: A cohort study of 400,000 people', *Health & place*, 45, pp. 32-38.
