# Supplementary material for: Where Are the Irish in Research on Ethnic Health Inequalities in Britain? A State‐Of‐The‐Art Literature Review
Source: Sociol Health Illn. 2025 Jan 2;47(1):e13874. doi: 10.1111/1467-9566.13874 (PMC11694090; doi:10.1111/1467-9566.13874)
Supplement: Supplementary file 2 — Supporting Information S2 [file SHIL-47-0-s002.docx]

**Online Appendix: Summary table of findings of 140 papers included in state-of-the-art review, chronological order**

| **Author(s)** | **Year** | **Research objectives** | **Methodology** | **Results** | **How Irish constructed** |
| --- | --- | --- | --- | --- | --- |
| Harding and Balarajan | 2001 | Examine mortality of third generation Irish people (grandchildren of migrants from the Republic of Ireland) | Secondary data analysis of the ONS Longitudinal Study of England and Wales, Mortality figures 1971 to 1997. | In terms of socio-economic status (access to a car, housing tenure), first generation Irish migrants were the most disadvantaged and third generation were least disadvantaged. In terms of mortality rates, the rate increased such that third generation had higher mortality rates than first generation. However, this was attenuated for first generation migrants by adjusting for socio-economic variables (but was still significant for the second and third generation). | Third generation Irish: grandchildren of migrants from Republic of Ireland. Dataset was from before 'White Irish' included in Census, so no way to categorise other than country of birth. |
| Landman and Cruickshank | 2001 | To distinguish between ethnicity/migration/generation and health outcomes, particularly of nutrition | Literature Review Study | Compared with people born in England & Wales, South Asia, Caribbean, West Africa, East Africa, and Scotland: Men born in Ireland had the highest standardised mortality ratio (20-69 years, 1989-92) of all causes of death, while Irish women had the third highest (after Scotland and West Africa). Looking at specific causes of death Irish men had the highest SMRs for lung cancer, Irish women the second highest (after Scotland). Coronary heart disease (CHD) and stroke higher among South Asian and Caribbean / African groups. Irish had relatively low rates of diabetes. Irish Catholics in Scotland had higher hip-to-waist ratio than non-Catholics. One study found older Irish Catholic (descent) men and women to be shorter than Protestant equivalents, and linked this to social disadvantage over the long term. Much of the data presented on nutrition and diet does not have info for the Irish specifically. | Looks at Irish migrants in England & Wales (and second generation migrants), and people of Irish Catholic descent in Scotland. Refers to migrants from Ireland to England/Wales as 'internal migrants' and in the same category as migrants from Scotland, incorrectly implying Republic of Ireland is part of the UK. |
| Livingston, Leavey, Kitchen, Manela, Sembhi and Katona | 2001 | To compare dementia and depression prevalence among older migrants with that of the UK born. | Quantitative primary data collection, cross-sectional. Relative risk of morbidity. Logistic regression for determinants of dementia or depression. Chi-squared and Mann-Whitney tests. | Irish born significantly lower risk of having dementia compared with the UK born, but in logistic regression no longer statistically significant. African-Caribbean significantly higher risk of dementia than UK born. No significant difference by country of birth in likelihood of having depression. | Interested in country of birth - people born in Ireland ("migrants"). Mentions that people born in Ireland who put 'British' down as their ethnic group have made a mistake. Assumes that country of birth is the sole determinant of ethnic identity. |
| Van Cleemput and Parry | 2001 | To gather data on the health status of Gypsy Travellers in Sheffield and compare it with UK residents of similar characteristics. | Face-to-face (FTF) interview based on previous study that identified low literacy rates among Travellers. | In many measures the Traveller group had significantly poorer health compared with the UK general population, but for overall EQ5D score was not significantly different from the 'matched' group (who were similar in terms of urban deprivation). However, when looking at individual items on the EQ5D index, Travellers had significantly worse scores on mobility, usual activity, perceived overall health problems compared with the 'matched' group. When colloquial wording for depression and anxiety (fed-up and nerves) was used, the Traveller group was significantly worse than the 'matched' group. Travellers who travelled more frequently had better scores. | Gypsy Traveller group included people of both Roma and Irish background, so not exclusively about Irish Travellers. |
| Abbotts, Williams & Ford | 2001 | Trying to ascertain if socio-economic disadvantage can explain the health inequalities experienced by Catholics (who are primarily of Irish descent) in Scotland | Using data from the longitudinal West of Scotland Twenty-07 Study. Three waves (1988, 1990, 1995). Logistic regression and ANOVA. Some cross-sectional analysis and some longitudinal. | Catholics had worse health in all self-rated measures and lung function, but when socio-economic factors were added to the model the odds ratio reduced and became non-significant. Longitudinal analysis looked at the direction of the relationship between socio-economic status (SES) and health. Health at age 56 did not predict SES at age 63. However, SES at age 56 did (partially) predict health (self-rated health, number of symptoms in the last month) at age 63. | Studied people of Catholic descent in Scotland, mostly of Irish descent. |
| Bracken & O'Sullivan | 2001 | A discussion article commenting on the lack of interest in researching/making policy on Irish health in Britain despite there being clear evidence for health inequalities in this group. | Literature review and comment piece | The Irish in Britain are "not different enough" to count as a minority ethnic group as far as researchers are concerned. | Describes the Irish in Britain as holding a "problematic and liminal position" (p.44) and that this is apparent in how they are treated (or not examined) in the research literature. Fails to recognise that there are some Irish people who are not White. |
| Malone | 2001 | Brings together a number of data sources (primary and secondary) to describe in depth how a community of Irish immigrants was formed in London, how they maintain this community, and its relevance for their health. Focus is on health beliefs. | Primary qualitative, secondary qualitative (newspaper sources), secondary quantitative (surveys). Content analysis was used for interview/focus group discussion (FGD) transcripts, and 'textual' content analysis was used for the literature and Census/local authority (LA) info. | There was a strong sense of place within community; which parts of the local area with 'in' and which were 'out'. Geographical boundaries to the sense of 'our' community group. Within that, people formed smaller groups based on similarities with other immigrants, e.g. from the same area back in Ireland. Irish migrants living in other parts of London were considered 'other', unless they had some other shared bond (e.g. from same place 'back home'). There was a strong culture of sharing of scarce resources within the community, e.g. one ladder in the whole community was shared between families as and when they needed it. This was partly explained by the exclusion of the Irish from the established British 'helping networks' (due to being immigrants and not part of the old boys network) and so they had to develop 'helping networks' of their own. This community seemed to have a lower level of mental ill health than other Irish people outside of this community. The community was not political; they didn't sympathise with Republican causes. Unlike other groups of Irish people they didn't blame their disadvantage on the British. Perception of health was shaped by being able to do what they wanted to do (do all their wanted/usual activities). Wanted to reinforce a lifetime of hard work and how they have not had to depend on government handouts. Health is described as something separate from conditions or illnesses, e.g. a woman with hypertension who nonetheless described her health as good. There is a discussion of blame in relation to ill health and some attempt to link this to the centrality of guilt and sin in Irish (Catholic) culture. Being owner-occupiers was seen as central to the sense of 'community saved' that these participants had. Owning their homes seems to have come about by having certain time-dependent opportunities (e.g. opportunity to purchase council houses, some advantages for people in the construction industry). Having a stable property situation enabled the formation of helping networks, and indeed encouraged their formation. A key characteristic of this group was that they did not want to return 'home' and had negative views about how life was back home. Not the rural idyll that other migrants might relate to. | Irish immigrants. |
| Saxena, Eliahoo and Majeed | 2002 | To compare children's health status and use of services by ethnicity and SES | Health Survey for England 1999. This one had a booster sampling for ethnic minority groups. Secondary data analysis. Logistic regression. | Irish children significantly higher odds of acute illness in past two weeks compared with general population. Most other groups either significantly lower (Bangladeshi, Chinese) or no significant difference. Irish and Afro-Caribbean children had highest rates of asthma. Looks like the Irish were the most frequent users of hospital outpatients, and most frequent hospital inpatients, but no significant differences in these once other factors controlled for in multivariate regression. | Irish included as one of the ethnic groups under study and compared with the 'general population' group, but not mentioned if Irish is measured as country of birth or ethnic identity. |
| Aspinall | 2002 | To look for evidence for or against Leavey's (1999) theoretical explanations for high rates of Irish suicide | Literature review | Leavey says that the Irish have higher rates of being single/divorced/living alone; Aspinall shows figures that they are not. Leavey says the Irish are more socially isolated; Aspinall attempts to counter this with figures on perceived support. Leavey argues that Irish migrants are economic migrants but don't end up doing well financially or settling down in one place, and they constantly have the expectation of returning home; Aspinall mainly refutes this by saying qualitative findings are anecdotal, and the samples were biased toward those who were in touch with services due to (alcohol) problems. Leavey says the Irish are less likely to use GP services; Aspinall says that the data that exist don't show any patterns, but are flawed because they don't adjust for level of need. Leavey says the Irish may not seek help for pain/suffering because of religious/cultural attitudes of stoicism; Aspinall says there is a mixed picture of evidence for this, nothing consistent. Regarding impact of alcohol or anti-Irish racism on suicide, Aspinall says there is not enough evidence. | Ireland as country of birth. Datasets from before White Irish included as an ethnic category. |
| Curran, Bunting & MacLachlan | 2002 | To understand the relationship between mental health and acculturation among the Irish in Coventry, using the ethnic density hypothesis. | Primary quantitative data collection (questionnaires) and some open questions (which were analysed using content analysis). Factor analysis, correlation. | General Health Questionnaire was significantly negatively correlated with the marginalisation and segregation factors of the Trinity Acculturation Scale (TAS). The people who expressed an intention to return to Ireland had significantly higher assimilation scores. Older participants were significantly more integrated. Gay/Lesbian participants did not want to return home due to how they would be treated, but they may also have faced rejection from their Irish peers in Coventry. Women were less likely than men to be integrated. | Ireland as country of birth. |
| Livingston, Leavey, Kitchen, Manela, Sembhi & Katona | 2002 | To examine health service use/accessibility among older immigrants in Islington, London, and to attempt explanations for any differences. | Face-to-face interviews. Chi-squared tests, Mann-Whitney U test. Relative risks, probability of using services. Logistic regression. | Those born in Ireland had a significantly lower relative risk of dementia compared to those born in the UK. Those born in Africa/Caribbean had significantly higher relative risk of dementia. Those born in Cyprus had significantly more depression symptoms and subjective health problems. The Irish and Cypriots were significantly more likely than UK born to attend the GP. The Cypriots were also significantly more likely to attend outpatient services and hospital medical services. Cypriots and Africans/Caribbeans were significantly less likely than UK born to have informal care. | Ireland as country of birth. Irish included as among the "immigrant elders" studied. |
| Moselhy & Telfer | 2002 | Descriptive study looking at substance misuse among minority groups compared with the British population. | Retrospective record review of psychiatric records from 1992-1995. Chi-squared tests. | The Irish were younger at first use of drugs than the British, Asian, or Afro-Caribbean groups. They were also had the highest percentage of having previously been in prison, lowest percentage having stable accommodation, lowest percentage keeping up a family relationship, and all were unemployed (but these factors were not significantly different from the Caucasian British group). They Irish almost exclusively used opiates, but one used benzodiazepines. The authors mention that since many of the Irish participants had poor education/SES they may have been illiterate, and therefore word-of-mouth would be a more effective way of accessing services for this group. They relate this to a particular feature of the Irish immigration experience. Their explanation for why the Irish may have been more likely to re-use needles is linked to the idea of sin, and that having clean needles would indicated "premeditated" drug use which is argued to be a worse sin than spontaneous drug use. | Irish are referred to as one of the 'Caucasian' groups at one point, and as one of the 'ethnic minority' groups at another. Comparison group is the British Caucasian. No recognition that it is possible to be a person of colour and Irish. |
| Commander, Odell, Surtees & Sashidharan | 2003 | Descriptive study looking at use of psychiatric services among minority ethnic groups. | Review of records and some data gathered from staff members. Chi-squared tests. | Percentage primary diagnosis in each ethnic group (psychosis/bipolar or depression/neurosis) were very similar between Irish and White. Black Caribbean much more likely than any other group to have psychosis/bipolar as their primary diagnosis. Black Caribbeans more likely to be detained under the Mental Health Act. | Includes the Irish-born in a list of 'ethnic minorities'. Irish category was assigned based on country of birth, but the remainder were from the 1991 Census ethnic categories. |
| Foster | 2003 | Discussion paper highlighting the need for research on Irish alcohol misusers. | Literature review | Discusses a study by Harrison & Carr-Hill that classified the Irish in Britain into two key groups: 1st was stable in terms of accommodation and "socially", 2nd was transient, "socially unstable", low level of education, and was mostly older men. The latter group was more likely to have alcohol problems compared with the general population in Britain. Alcohol problems got worse with migration. Other literature shows that alcohol misusers are not as assertive as drug misusers when it comes to approaching services and asking for help. It also seems that the Irish alcohol misuser is more likely to be treated within psychiatric inpatient services than within specialist alcohol services (which may be better suited for them). | Says that there has been relatively little research on Irish alcohol misusers despite the stereotype of alcoholic Irish. No mention of Irish people of colour. |
| Walls & Williams | 2003 | To examine experiences of discrimination at work among Irish Catholics living in Glasgow, linking to their health experiences. | Qualitative. Analytic induction. | The participants discussed discrimination they had observed or experienced in the workplace. Participants made an effort to distance themselves from the discrimination, so it was the fault of other people or the system. Accounts of some employers just not employing any Catholics, or very few, but saying that things have changed more recently. Catholics talking about not getting job offers because of Catholicism, and some interesting examples of Catholics 'passing' as Protestants, but then being sacked once they were 'found out'. Discrimination was apparent in that Catholics were less likely to be offered white collar jobs, while blue collar jobs were open to them. | Focuses on Catholics in Scotland of Irish descent. Participants spotted other people's Catholic identity not by church attendance, but by having attended a certain school, having a certain name, or supporting a certain football team. |
| Abbotts | 2004 | Discusses how the White Irish category in the 2001 census came to be, and how strong the evidence base for health inequalities is. | Literature review | The Midspan Study (published in 1998) showed elevated mortality for men with an Irish surname when looking at deaths from all causes or from coronary heart disease. This is compared with a lot of literature on mortality rates that also found elevated mortality rates for Irish men and women in other parts of Britain (notably, Harding/Balarajan/Marmot). The Twenty-07 Study found health disadvantage for Catholics even after controlling for sex and social class in terms of: self-rated health, symptoms in the last month, sadness/depression in the last year, eyesight, self-rated disability. Mentions Evandrou (2000) which pooled GHS from 1991 to 1996, one of the few studies to include Irish older people, higher limiting long-term illness (LLTI) and acute illness/injury for Irish men, higher 'not good' general health for Irish men and women. | People of Irish Catholic descent in the west of Scotland have health disadvantages. Many of these people do not identify ethnically as Irish, so it is only possible to identify them through their religion. |
| Abbotts, Harding & Cruickshank | 2004 | Examines the Health Survey for England (HSE) to look for patterns in cardiovascular risk for UK-born Irish people and UK-born Caribbeans living in England. | Secondary data analysis of HSE 1999. Chi-squared tests, t-tests, regression. | Bivariate: Aged 35-44 Irish men more likely to smoke than the general population (so were Caribbean men); lower blood pressure (BP) than general population men; Irish women shorter than general population women. Aged 45-54 Irish men more likely to smoke than general population; some other differences significant at bivariate but disappeared when controlling for smoking and SES. | UK-born Irish using self-reported ethnicity and country of birth. |
| Aspinall & Jacobsen | 2004 | Executive summary of official NHS report 'ethnic disparities in health and health care' | Descriptive analysis | Among children, Irish girls were more likely than the general population to have ever smoked. Respiratory symptoms more common among Irish, Black Caribbean, and general population children, than the other minority groups. | Just 'Irish', not much explanation of measurement. |
| Bhugra | 2004 | Literature review to examine evidence of how migration affects mental state and help seeking | Literature review | Migrants who come from further away have more stress. Migrants who do not speak the language of the host country fluently have more stress. Forced migrants have more stress than voluntary migrants. Forced migrants are more likely to end up in menial jobs with more stress. Culture shock may be greater for young adults who are still in the process of identity formation. Migrants who have to deal with legal or visa issues have more stress. Migrants from "socio-centric societies" may find it harder to adjust to "ego-centric societies". Raises the issue of the relationship between the home country and the host country, and whether there are any tensions. Greater country tensions are likely to impede the migrant's adjustment to the new life. | Irish as migrants. |
| Leavey, Sembhi & Livingston | 2004 | Exploring some of the hypotheses that have been proposed about the higher rates of suicide among the Irish in Britain: the issue of identity, the losses involved in being a migrant, and the myth of return. | Qualitative. Focus group discussions and individual interviews. | Reasons for migration were economic, participants talked about starvation conditions in the 1940s. Women also talked of the oppressive culture in rural Ireland and they wanted to escape and find freedom. Migration was a normative event for rural people because any land could only support the eldest son. Many had a strong sense of independence. People described culture clashes where Irish turns of phrase or types of humour were perceived as evidence of madness or stupidity by the English. The men tended to arrive without jobs and managed to fairly quickly get work in construction or demolition, but this was linked with living in over-crowded accommodation with poor facilities, or moving for work a lot, and therefore being very lonely and not putting down any roots or forming romantic attachments. The living conditions for men were so devoid of recreational space or opportunity that the pub became the only place to relax (five to a bedroom with no radio or TV or place to sit). The women tended to be recruited while still in Ireland by English agencies looking for domestic staff. Most discussed an assumption early on that they would return to Ireland once they had made their money, but this had not happened. There was a strong recognition that the Ireland they knew had changed, and that most of the people they knew when young had died or moved away. | Irish migrants. |
| McCambridge, Conlon, Keaney, Wanigaratne & Strang | 2004 | To compare drinking patterns among Irish men in London and Dublin | Questionnaire. Data collection took place in the pubs. People who were already intoxicated were excluded. Multiple regression. | The London participants had greater average units of consumption in the last week compared with the Dublin participants (63 v 43), and greater mean number of drinking days in the past month (20 v 14). Frequency of drinking and unit consumption was still statistically higher in London than Dublin after controlling for other factors in the regression. London sample significantly more likely to say an adverse effect of alcohol on your health is a reason to change drinking behaviour. Dublin sample significantly more likely to say that spending most of your leisure time drinking is problematic. Male gender and younger age was associated with greater risk in both locations. Argues for early intervention for alcohol problems with younger Irish people in Britain. | Born on the island of Ireland. |
| Scally | 2004 | A commentary piece taking a historical view of how the Irish have been perceived in England, including as a health threat. Is a call for research to focus its effort on addressing the poor health of the Irish in Britain. | A commentary with evidence from academic, policy, and popular literature. | The Royal Commission on the Irish Poor (1836) discussed the Irish in Ireland but also the Irish in Britain, about how the Irish tended to present health problems as commonly located in pain or oppression around the heart even if the cause of illness was nothing to do with the heart. Tells the story of Kitty Wilkinson who helped people with cholera in Liverpool in the 1800s, and is credited with setting up the first public wash-house. | Shows a progression in how the Irish were perceived in the 19th century as a source of contagion to now being in poorer health / greater risk. |
| Walls & Williams | 2004 | Looking at reasons for leaving jobs and trying to relate it to Protestant versus Catholic differences, thereby attempting to link to poorer health among Irish Catholic descent people in Glasgow. | Qualitative. Analytic induction. | Many reasons for leaving jobs were shared between Catholics and Protestants and signified normal life events such as childcare, seeking new opportunities, or redundancy. There were some reasons that Catholics were more likely to give than Protestants, however. These included poor health (particularly work-related stress), difficulties with colleagues, going self-employed as a means of escaping difficulties as an employee, going bankrupt, or lack of prospects/promotion. | People of Catholic descent in Glasgow |
| Weich, Nazroo, Sproston, McManus, Blanchard, Erens, Karlsen, King, lloyd, Stansfeld & Tyrer | 2004 | To establish the prevalence of common mental disorders among Britain's ethnic minority groups. | Survey and standardised clinical interview. EMPIRIC survey. | At bivariate level Irish men were significantly more likely to have common mental disorders compared with White men, but when adjusted this only remained significant at the 35-54 ages, and was not affected by SES adjustment. Irish women were not statistically significant from White women at either bivariate or multivariate level. | EMPIRIC dataset. Irish included the Irish-born or those who had an Irish-born parent. The rest were identified using the 1991 census ethnicity categories. |
| Bhui, Stansfeld, McKenzie, Karlsen, Nazroo & Weich | 2005 | To establish the link between racial discrimination at work and common mental disorders. | Secondary analysis of the EMPRIC survey. Logistic regression. | At multivariate level, being insulted because of one's ethnicity was significantly associated with a higher risk of common mental disorders among the Irish, Indian, and Bangladeshi participants. | At one point they refer to "non-white groups" and include the Irish in this. No mention of whether the Irish were white or people of colour. |
| Crawford, Nur, McKenzie & Tyrer | 2005 | To look at how common suicidal ideation, attempted suicide, and service use related to attempted suicide are among ethnic minority groups in England. | Secondary analysis of the EMPIRIC survey. Logistic regression. | Irish men had significantly higher odds of suicidal ideation "lifetime thoughts of taking own life" compared with White British. When adjusted for age, the significance persisted in only the men aged 35-54. Irish women not significantly different from White British women. Only other significant differences were for Pakistani men (less likely), Bangladeshi women (less likely), and Pakistani women aged 35-54 (less likely). No significant differences between Irish and White British in attempted suicide. Among the Irish, specific demographic factors significantly associated with suicidal ideation were being unmarried, having a long-standing physical illness, having a high CIS-R score (and therefore likely to have a diagnosable common mental disorder), and difficulty getting on with others. The older Irish group (55-74) were significantly less likely than the youngest age group to have suicidal ideation. White British and Irish had higher rates of service use following a suicide attempt compared with other ethnic groups. The measures of acculturation or British identity had hardly any effect on suicidal feelings, so the acculturation hypothesis was not upheld. This variable was significant for the Irish, and could relate to feeling out of place or feeling a sense of hostility from the British. | EMPIRIC dataset. Irish included the Irish-born or those who had an Irish-born parent. The rest were identified using the 1991 census ethnicity categories. |
| Fitzpatrick & Newton | 2005 | Editorial reminding GPs to consider the mental health needs of Irish patients. | Comment piece, drawing on literature | Identifies the poor health of the Irish in Britain, particularly in terms of suicide and mental health. Argues that the Irish have been ignored because race relations usually prioritise skin colour over country of birth. Also mentions that the Irish in Britain have experienced racial discrimination, e.g. "no blacks or Irish", backlash over the Troubles. The 2001 Census showed 15% of the Irish reported their health as "not good" compared with 10% of the general population. Explains what culturally competent services are, gives some examples of Irish-specific services in Liverpool and Islington. Argues that such services are small and their funding sources are insecure. Argues that government needs to commit to supporting such services. Argues that GPs need to capture the ethnicity of their patients, including measuring Irishness. | Refers to data for "the Irish" throughout, when in some cases they are referring to date for "White Irish" Census category. |
| Karlsen, Nazroo, McKenzie, Bhui & Weich | 2005 | To relate experiences of common mental disorder (CMD) with experiences of racism. Particular focus on psychosis. | Secondary analysis of the EMPIRIC survey. Regression. | 7% of the Irish reported experiencing racism (either verbal or physical) in the last year. This compares with 15% of Caribbean, 13% of Pakistani, 12% of Indian, and 9% of Bangladeshi. The Irish (7%) and the Bangladeshi (8%) had the lowest percentages of experiencing employment-related discrimination (36% Caribbean, 19% Indian, 16% Pakistani). In terms of beliefs about British employers being racist, the Bangladeshi (65%), Indian (36%) and Pakistani (36%) were least likely to think British employers were racist. This compares with only 11% of Caribbeans and 15% of the Irish who believed British employers were not racist. When all ethnic groups were combined, experiences of racist abuse, employment discrimination, and beliefs of employer racism were all significantly associated with risk of common mental disorder. Racist abuse and employment discrimination were also significantly associated with risk of psychosis. In the regression that looked at each ethnic group separately, experience of verbal or physical racist abuse was significantly associated with higher risk of CMD, and a belief that British employers are racist was also significantly associated with higher risk of CMD. No relationship with psychosis for the Irish. | EMPIRIC dataset. Irish included the Irish-born or those who had an Irish-born parent. The rest were identified using the 1991 census ethnicity categories. |
| Collingwood Bakeo | 2006 | To examine England & Wales death records of infants to see if there are any variations by country of birth (CoB) of the mother. | Analysis of official records. Only singleton live births included. So, does not include stillbirths or deaths of infants who were part of multiple births (twins, etc). | Death due to congenital abnormalities highest where mother's CoB was Pakistan (more than half), Bangladesh (more than half) or Republic of Ireland (RoI) (less than half, but next largest). Overall, rates of infant mortality declined from 1983-89 to 1990-2001, and the steepest decline was for RoI mothers. Infant mortality rate (IMR) was highest overall for mothers born in Pakistan, followed by West Africa, and the Caribbean. For RoI mothers, IMR was 50% higher where the father was in the manual social class compared with the non-manual class; this was the largest difference among all countries of birth. | Death certificates record mother's country of birth (RoI) but do not record ethnicity. Second/third generation migrants therefore not captured. |
| Ryan, Leavey, Golden, Blizard & King | 2006 | To test the hypothesis that the higher rate of depression and suicide among Irish migrants to Britain is due to poorly-planned migration | Case-control study. Logistic regression. | The group with depression were disadvantaged on several measures compared to the group without depression; they were more likely to be unemployed, less likely to own their own home, more likely to have experienced discrimination, less likely to be acculturated, and more likely to misuse alcohol. They were also more likely to have a high score on the "unplanned migration" measure, even when adjusted for age and gender. Men with depression were significantly more likely than women with depression to be unemployed, less likely to own their own home, and less likely to be married or have children. They had higher Hospital Anxiety and Depression Scale (HADS) and Beck Depression Inventory (BDI) scores but were no more likely than women to be receiving treatment. History of childhood emotional abuse was significantly related to depression when gender was combined, and for women (but not men) when gender was considered separately. Acculturation was not relevant in the adjusted model. Low level of social support was significant, as was unemployment for men, discrimination (for women). In the model adjusting for pre-migration factors, poorly planned migration was significant for men but not women. In the model adjusting for post-migration factors, poorly planned migration was not significant for either men or women. | Irish-born. |
| Scanlon, Harding, Hunt, Petticrew, Rosato & Williams | 2006 | To understand barriers to cancer prevention and diagnosis among Irish people in Britain, compared with White British people. | Qualitative. Interviews and focus groups. Miles & Huberman's qualitative analysis. | Narratives: Knowledge of cancers and their signs and symptoms; attitudes to cancer; fear; secrecy; denial and stoicism towards personal risk; beliefs about cancer causation; socio-economic and environmental factors; stress; interaction with health services. Overall, there were similarities between White British (WB) and Irish in their use of narratives about cancer. Poorer knowledge of cancer symptoms was found among people of lower SES in both ethnic groups, but also specifically among first generation older Irish. Both ethnic groups were afraid of cancer and equated it with a death sentence. However, the Irish used "more pessimistic language and imagery, such as 'a demon', 'a terrible curse', 'a scourge', 'a scab' and 'being riddled with it'." (p.331). The authors made a link between the Irish having contact with people whose cancer was detected late and died, so that may be partly behind the difference. Older Irish participants also spoke about stigma and the need to keep cancer secret for fear of bringing shame on the family. The Irish participants thought that Irish men were particularly unlikely to talk about cancer due to a macho culture, and was linked to having to become strong due to post-migration discrimination and racism. The Irish had beliefs that cancer was common in their families because it was a "curse". Authors relate this to the Irish having bigger families. Many of the Irish participants linked cancer to the harsh socio-economic circumstances that were faced back home and later in manual jobs in England. The Irish were also more likely to blame alcohol as a cause of cancer, and linked this to the social networks of the Irish men in Britain being limited to the pub. The Irish identified stress, trauma, social isolation, and a sense of not being wanted in Britain to causing cancer. The lower SES male Irish participants tended to delay seeking help from health services. Older Irish participants spoke about a preference to not know if they have cancer or not. There is a link between machismo and not going to the doctor; going to the doctor is described as "for softies" (p.336). Finally, the younger Irish participants spoke about their parent's generation as having a passive patient role and not wanting to bother the doctor. | Irish includes both Northern Ireland (NI) born and Republic of Ireland born, and includes 2nd & 3rd generation as well. |
| Sproston & Mindell (Eds) - summary report | 2006 | To summarise the headline findings of the Health Survey for England 2004 on the health of minority ethnic groups in England | Interviews and health tests. | Irish not significantly different from general population on self-reported bad or very bad general health. The graphs/tables are not shown for each outcome so it is quite difficult to ascertain the pattern of Irish people from the narrative alone (they are often not mentioned at all). It appears as though there was no significant difference between the Irish and the general population on severe lack of social support. Irish men had the highest prevalence of cardiovascular disorder, but not mentioned if this is significantly different from any other group. Irish in lowest income category had significantly higher risk of cardiovascular disease, ischaemic heart disease, or stroke than the Irish in the highest income category; this was said to be the same pattern in the general population and Black Caribbean women. Irish not significantly different from the general population in diabetes. Irish and Bangladeshi men significantly more likely to smoke than general population, even controlling for age. Irish and Black Caribbean women were the most likely to smoke. There was a trend over time between 1999 and 2004 for fewer smokers in the Irish groups. The Irish drank on more days in the past week than the general population, were the most likely to exceed the recommended daily limit, and had the highest percentage of binge drinking. Obesity was highest among Irish and Black Caribbean men, but when age standardised Irish were not significantly different from general population in terms of obesity. Irish not significantly different from general population in terms of brisk walking. Irish actually more likely than general population to meet the physical activity recommendations. No significant difference in hypertension between Irish and general population. Irish men not significantly different from the general population in terms of eating 5 portions of fruit and veg a day, but all the other minority groups significantly more likely than general population. Irish least likely to use salt in cooking, although more likely to add salt at the table without tasting food first. Anaemia zero among Irish men and Irish women had the lowest levels of anaemia of all the minority group women. An indicator of diabetes and vascular complications (glycated haemoglobin) was significantly lower among the Irish than the general population. Irish not significantly different from general population in terms of using complementary or alternative medicine. Irish girls the most likely to have ever smoked a cigarette. Irish girls (aged 8-15) the most likely to have ever drunk alcohol (boys similar to general population). | The Irish included as part of the ethnic minority boost of HSE 2004. The Irish referred to as one of the "seven largest minority ethnic groups in England". Irish people were those who were born in Ireland or who had at least one parent born in Ireland. Note, not 'white Irish'. However, the researchers did make an assumption that Irish people were white, because they said that the focused enumeration technique (where householders are asked if people living either side of their address are from one of the minority ethnic groups) only works for "visible" minority groups, and they did not use this technique for Irish people. |
| Tilki | 2006 | To understand the drinking culture of Irish men in London | Qualitative, following Miles & Huberman. Focus Group Discussions and individual interviews. | Describes the cohort of men who migrated from Ireland in the 1950s and 1960s tending to work in the construction industry and moving around the country to follow work. Tilki argues that the pub was the natural place to go after work (rather than home) because it was where they were recruited from, picked up for casual labour, dropped off afterwards, and paid. It seems to be a cash-in-hand, non-official employment status. Moreover, men who decided not to remain in the pub after work were considered "poor mixers" (p.251) and likely not to get another job offer. A further reason to congregate in the pub is that it was deemed a 'safe space' to meet with other Irish people in the context of anti-Irish sentiment during the Troubles. There is a contrast made between the Irish solo migrant and the migrants of other ethnic groups who were assumed to migrate with extended families. Irish men live in 'digs' and there is no entertainment and no visitors allowed. Tilki says there were "significant numbers" of Irish speakers with limited English. Pubs were said to have an oral culture of "slagging", whereby a flattened hierarchy outside of the typical construction hierarchy could be achieved. The culture of buying rounds was seen as compulsory (you couldn't refuse a drink, nor refuse to buy a round), and this resulted in about 10 pints a night and maybe spirits too. A link is drawn between the ability to drink heavily without becoming intoxicated and a sense of masculinity. Physical strength in the construction industry was also a key part of masculine identity, and those who became sick and were unable to work had a crisis of masculine identity. In times of sickness, physical, or mental pain, alcohol was turned to as a familiar and socially acceptable 'medicine'. Tilki says "there is now widespread evidence that young people fleeing institutional and family abuses in Ireland from the 1950s until the 1970s came to Britain " (p.255). This is the first paper that's mentioned institutional abuse. An industrial school in Dublin (not named) is mentioned. Family sexual abuse is mentioned by another. | Those born in Ireland |
| Wild, Fischbacher, Brock, Griffiths & Bhopal | 2006 | To examine differences in mortality rate from cancer by country of birth (in England & Wales) | Standardised mortality ratios and 95% confidence intervals. | Irish men and women had statistically significantly higher standardised mortality ratios from cancer than the general population, and in fact were the highest overall for men and second highest for women (after Scotland). Lung cancer was significantly higher for Irish and Scottish men and women compared with the general population. Most other minority groups significantly lower than general population. Breast cancer and prostate cancer were not significantly different for the Irish compared with the general population. Colorectal cancer was higher for Irish men and Scottish men and women compared with general population. The link between smoking and lung cancer is made. A link between colorectal cancer and eating meat/fat is also made. | Those born in Ireland (both NI and RoI examined together) |
| Fischbacher, Steiner, Bhopal, Chalmers, Jamieson, Knowles & Povey | 2007 | To examine differences in mortality rate from coronary heart disease by country of birth (in Scotland) | Standardised mortality ratios. | Looking at all causes of death, men born in the Republic of Ireland had significantly higher mortality ratios than the Scottish born; all other groups had lower mortality ratios than the Scottish born. Both Irish-born men and women had significantly higher mortality than England & Wales resident population. Looking at deaths from coronary heart disease, Irish born not significantly different from Scottish born (but Irish born were sig higher than England & Wales residents). Same pattern found for stroke. | Country of birth - RoI |
| Leavey, Rozmovits, Ryan & King | 2007 | To understand causes of depression among Irish-born people living in London | Qualitative | There are three types of migrants: escapers, adventurers, and a mixed group. The escapers were trying to escape trauma or oppression in Ireland, including sexual abuse, religious oppression, or early parental bereavement. The adventurers tended to be men who had no difficulties in their early lives, but once in England did not settle down or stay in one place. The third group tended to be quite resilient and focused on career or family life. There was a sense that short-term migration to England was a normal part of a young Irish person's life, and tended to happen in an unplanned way. This lack of planning sometimes led to difficulties that were later said to be responsible for depression. Female escapers were trying to gain freedom from menial labour and abuse/exploitation in Ireland. Unwanted pregnancy is also mentioned, as is sexual abuse, and homophobia. The religious school system was also a source of life-long trauma. Some Irish women on arrival in England rushed into marriage with Irish men. Others relished the opportunities for socialising and novelty of living in a new country. Irish men tended to live in small poorly-equipped 'digs' and spent their social time in the pub. Loneliness and homesickness were evident. Many participants have returned to Ireland and then come back to England several times over their lives. The younger participants were more aware of the problems caused by drinking, and those who were not depressed were afraid of the lure drinking culture could hold. Alcohol was discussed spontaneously by the participants both as a normal part of life and as a coping strategy; for some it became a source of mental health problems. Some of the women discussed physically abusive alcoholic husbands as a cause for depression. Depression is also linked to chronic pain or caregiving duties. Experiences of "daily racist hassles" (p.239) were described as well as discrimination and attention from the police, but these were not linked by the participants to depression. | Country of birth - RoI |
| Millett, Saxena, Ng, Mainous, Majeed | 2007 | To examine change over time in diabetes management among different ethnic and SES groups in England | Secondary data analysis | The White Irish had significantly better cholesterol and blood pressure control improvements over time when compared with the White British group. The patterns for the South Asian and Black groups were more mixed. Findings indicate good access to health services and good uptake of medications by the White Irish group. The change over time in prescribing: The White Irish had significantly lower increase of being prescribed insulin than the White British group, but significantly greater increase in being prescribed lipid-lowering and antihypertensive medications. | "White Irish people were considered as a separate ethnic group as they have been shown to experience significantly higher all-cause mortality than the national average in England and Wales" (p.414). However, they used the HSE, which did not actually use the 'White Irish' ethnic category but instead looked at people born in Ireland or with at least one Irish parent (though they did assume all such people would be white). |
| Parry, van Cleemput, Peters, Walters, Thomas & Cooper | 2007 | To report on the health of Gypsies and Travellers in England | Primary data collection. Chi-Squared, t-test, Wilcoxon, McNemar's test. | Gypsies and Travellers were less likely than the comparison group to have attended school at all, or to have gone to school after primary level. They were more likely to smoke, more likely to be an informal carer, and had significantly more children. Gypsies and Travellers had significantly poorer health, significantly more likely to have LLTI/Disability, significantly worse EuroQol-5D (EQ-5D) scores than the comparison group. No significant differences in diabetes, stroke, or cancer. Gypsies and Travellers significantly more likely to have bronchitis/emphysema, rheumatism, heart disease, eye/vision problems, asthma, arthritis, or nerve problems. Also asthma, anxiety, chronic cough/sputum. Rates of miscarriage were significantly higher among Gypsies and Travellers, but hypertension significantly lower. The Irish Travellers were significantly more likely than the UK Gypsies and Travellers to be depressed or anxious. Overall conclusion is that Gypsies and Travellers have significantly worse health than settled people, including those from other minority ethnic groups or economically disadvantaged white groups. People with worse health were more likely to live in settled accommodation than travel. | Gypsies and Travellers of both UK and Irish origin. Most findings not specific to only Irish Travellers. |
| Wild, Fischbacher, Brock, Griffiths & Bhopal | 2007 | To examine differences in mortality rate from circulatory diseases and all causes by country of birth | Sex-specific standardised mortality ratios. | Deaths from all causes for people aged 20+: both men and women born in Ireland had significantly higher SMRs than the general population. The same can be said for people born in Scotland, East Africa, West Africa, Bangladeshi men, Indian women, and Pakistani women. Aged 20-44: Irish men had significantly higher SMRs, Irish women did not. Aged 45-59; aged 60-69; and aged 70+: both Irish men and women had significantly higher SMRs. When looking at the three different causes of death (cerebrovascular disease, ischaemic heart disease, circulatory disease) Irish men and women all had significantly higher SMRs than the general population. | Country of birth. Cannot distinguish people born in RoI from those born in NI (or born pre-partition). |
| Harding, Rosato & Teyhan | 2008 | To examine change over time in mortality rate from coronary heart disease and stroke among migrant groups | Age-standardised and sex-specific death rates and rate ratios, 1979-83, 1989-93, 1999-2003. | Death from coronary disease (men): significantly higher among both NI and RoI born men compared with England & Wales, and the difference increased over time. Death from coronary disease (women): significantly higher among women from NI and RoI compared with England & Wales. Mortality from stroke was significantly higher among men and women from RoI, and for men this increased over time. Overall, however, death from coronary and stroke declined over time. | Country of birth. Separately for Republic of Ireland and Northern Ireland. |
| Puthussery, Twamley, Harding, Mirsky, Baron & Macfarlane | 2008 | To examine the attitudes of health care professionals working in maternity services toward minority ethnic patients | Qualitative. In-depth interviews. Grounded Theory. | The participants said that the second-generation Irish did not have any specific maternity needs that were different from white English patients. The stereotypes are of women who are oppressed by patriarchy and who must cover up (i.e. Muslim); women who can't make decisions on their own or who might refuse services. | The authors discuss "invisibility" of the second-generation Irish, implying Irish people were all assumed to be white. It's not explicitly stated, but the overall impression is that when participants talk about "migrants" they are talking about non-Irish migrants |
| Rao, Wolff & Marshall | 2008 | To understand alcohol use among older people in the UK, comparing people from English and Irish backgrounds | Primary data collection. Questionnaires. Chi-squared test, Mann-Whitney U test. Bonferroni correction. | The Irish participants were significantly younger than the English group. There were no significant differences between the Irish and the English in smoking, the Short Form Health Survey-36 (SF-36), or own past psychiatric history. However, the Irish were significantly more likely to have a family history of psychiatric problems (depression or alcohol misuse). The picture for alcohol was mixed; consumption was generally low in the sample. No significant differences between English and Irish in mean weekly units over the lifetime, but Irish had a significantly higher median score on the AUDIT scale (hazardous/harmful use). There were several significant differences that became insignificant after Bonferroni correction. Irish more likely to drink daily (especially the men), and more likely to drink above "sensible" limits, but not significant after Bonferroni. The English were much more likely than the Irish to have reduced their drinking over their lifespan. | Irish explicitly included as part of the BME population: "Among Black and Minority Ethnic (BME) populations within the United Kingdom, people of Irish origin show the highest prevalence of drinking above 'sensible' limits." (p.18) |
| Bhala, Bhopal, Brock, Griffiths & Wild | 2009 | To examine deaths related to alcohol in England and Wales by country of birth | Standardised mortality ratios. | Alcohol-related conditions: SMR higher for men and women born in Ireland compared with national average in England and Wales (same for people born in Scotland and men born in India). Hepatocellular Cancer (HCC) specifically: Irish-born not statistically significant. Scottish and Irish most likely to exceed recommended alcohol consumption (mostly men). | Country of birth |
| Bhavsar & Bhugra | 2009 | Historical paper that looks at the mental health of Irish migrants to Britain in the 19th century (1843-1853), focusing on the Bethlem Royal Hospital in London | Review of casebooks from the Bethlem hospital 1843-1853. | The Irish group had significantly longer admissions than the comparison group. Irish more likely to be diagnosed with mania than melancholia; comparison group was the other way round. Irish patients' problems were said to be more likely to have been caused by childbirth-related problems, "over-study, religious over-enthusiasm, and political over-enthusiasm" (p.190). Authors argue that the greater proportion of mania diagnoses among the Irish is analogous to the greater proportion of modern mania diagnoses among Black Caribbeans. Authors conclude that the Irish-born patients were unlikely to have emigrated because of the famine as they were not destitute or paupers. Instead, they make the assumption that they migrated in an earlier wave and were more likely to be "strong and resourceful individuals" (p.195). Unfortunately, the case books did not list year of migration. | Irish born. Includes both Catholics and Protestants. |
| Clucas | 2009 | To understand whether differences in health between Irish and British people can be explained by SES or ethnic identity | Secondary data analysis | In multivariate analysis controlling for all demographic and socio-economic variables, the White Irish still had significantly worse self-rated health (more likely to be in the 'not good' category rather than the 'fair or good' category). When looking at the three groups of Irish (RoI born, NI born, Great Britain (GB) born), the patterns differed. Although all three groups were still significantly in worse health compared to WB, the RoI born reduced quite a lot, the GB born increased their odds, and the NI born only slightly reduced when the control variables were added. Furthermore, of the NI born, those who self-identified as Irish had significantly poorer health than those who self-identified as British, even after adding the control variables. Turning to LLTI, White Irish significantly more likely to report LLTI than WB even in multivariate analysis. At multivariate, the NI born and UK born Irish were significantly more likely than White British to have LLTI, but the RoI born lost significance after controlling for age/SES. The first-generation differences are argued to be largely eliminated once their greater age and SES disadvantage is accounted for. However, the second-generation differences are not accounted for by these factors and must be due to something else. | This study looks at RoI born, NI born, and GB born Irish. Also looks at White Irish. No mention of Irish people who are not white. |
| Cohuet, Bukasa, Heathcock, White, Brown, Ramsay & Fraser | 2009 | To report on an outbreak of measles among Irish travellers linked to a single event (funeral attendance) | Measles is a "statutory notifiable disease" and so it was mandatory to collect the information that is reported here. | Most of the cases in the measles outbreak happened among Travellers who had attended or were linked to the funeral. After a few weeks cases in non-Traveller groups started to appear (but not as many), and some even appeared in Norway. The people who contracted measles had not been vaccinated; vaccine uptake is low in the Traveller community. This triggered a public health campaign to reach the Traveller community, including a Traveller specific radio station, and a mobile MMR vaccination unit. "Culturally sensitive health education" (p.1762) was provided, but doesn't say what this involved. | Irish Traveller ethnic group. |
| Harding, Rosato & Teyhan | 2009 | To report on changes over time in deaths from cancer by country of birth | Age-adjusted rates and rate ratios | Men born in RoI had significantly higher mortality (rate ratios) from all types of cancer compared with those born in England & Wales, and the higher mortality (rate ratios) increased over the three time periods. Lung cancer and colon cancer were also significantly higher for the Irish born men. For women born in RoI, they also had significantly higher rate ratios for all types of cancer compared with England & Wales born, and these increased in two of the three time periods. Lung cancer was also high for RoI born women, but breast cancer was not significantly different in two time periods and was significantly lower in one of the time periods. High rates of oral-pharyngeal and oesophageal cancers in men were linked to alcohol use. | Country of birth |
| Nilforooshan, Amin & Warner | 2009 | To compare different ethnic groups detained under the Mental Health Act 1983 who appeal their detention | Review of one year of records of Mental Health Act detentions and any appeals. | Only 4 people out of 232 had successful appeals. Significantly more White Irish and Black Caribbean people appealed when compared with White British. No statistical tests on which ethnic group is the most likely to be detained (because there is no denominator). In terms of absolute numbers the largest group detained were Black British Caribbean, followed by White British, and then Asian. | White Irish |
| Smith, Kelly & Nazroo | 2009 | To test whether health inequalities among ethnic minority groups persist into the second and third generation. | Health Survey for England 1999 and 2004. Stepwise logistic regression. | There was an overall trend for second generation to have better SES indicators than first generation in each ethnic group (including Irish), although the increase was quite small for Bangladeshi and Pakistani. This indicates general upward social mobility in the second generation. There was also an overall trend for health-related behaviours to more closely resemble the majority population by second generation. The first generation Irish had more abstainers than the second generation, and the second generation were more frequent drinkers than the first generation (in second generation more drinking 4-7 days a week than White, fewer drinking 1-3 days a week than White, and more abstainers than White). The Irish had significantly lower odds of fair/poor health in the second generation (compared with the first generation), but once controlling for age and sex this was not significant. When comparing White (English) with the Irish population, there were no significant differences in fair/poor general health at either first or second generation. Overall, when adjusting for SES, health worsened in the second generation. | Irish, not White Irish. Data from HSE, so likely to be born in Ireland or have at least one parent born in Ireland. |
| Tilki, Ryan, D'Angelo & Sales | 2009 | To report on social disadvantage experienced by the Irish in Britain | Primary and secondary data analysis. Census 2001 data. Labour Force Survey 2006. GIS mapping. Interviews with key informants. | The authors identified three types of Irish people who they considered to be specifically at risk: (i) Middle aged inactive and sick and isolated (MISIs), (ii) "Elderly people" (in general, but also men in particular), and (iii) "elderly women". The MISIs are likely to have taken early retirement due to ill-health and are now struggling financially, socially isolated, and depressed. The older men are likely to be never-married and living alone, they mention those in the construction industry who moved around a lot during life and they can't go back to Ireland. The older women are at risk because many of them have retired from the kind of occupations that had accommodation (like nursing) and now they have to live alone (or are homeless) in a new area (don't know how to manage a household, pay bills, etc), no social network and they can't go to the pub like men because it is not seen as acceptable. Other women may be widowed and their children have moved away, so they are now isolated. A further two groups they identified as having problems are (i) Irish Travellers, and (ii) Irish Prisoners. The problems that they identify as disproportionately affecting the Irish population are: (i) physical and mental ill-health, (ii) alcohol misuse, (iii) homelessness, (iv) institutional abuse, (v) domestic violence. Issues like machismo, stigma, shame, and pride are barriers to help-seeking among the Irish. There is also mention of cultural insensitivity from services acting as a barrier. Geographical areas with a high density of Irish people also tend to be socially deprived. Census 2001 - Irish men and women aged 50-64 more likely than White British to report LLTI/D. Irish women tend to be in higher occupational classes than Irish men, but the authors explain this by the way nursing is classified. The key informants talk about "particular resistance to residential care among older Irish people" (p.26), linked to fears about loss of control/independence, but also recognition that services do not celebrate Irish traditions/customs. The older Irish men who had worked in construction had been paid cash-in-hand. This led to them being afraid of asking official services for help with health/disability/benefits because they were worried they'd be forced to pay taxes that they owed. | White Irish ethnicity and country of birth (RoI) |
| Clarke | 2010 | A discussion paper using the lens of diaspora to understand the mental health of Irish people in England (including historical perspective) | Contrasts theoretical depictions of the Irish in Britain living in the land of the "oppressor" with the qualitative data from Leavey 2007. | A commentary on whether alienation was a cause of depression described by Leavey's participants. "Unexpectedly, the study’s sample did not speak of anti-Irish hostility as a source of their mental distress. Which is not to say that they hadn’t experienced insults and racism, but that they didn’t think it induced their depression. The situation is complicated by virtue of participants denying the relevance of racism whilst revealing it as a constant source of antagonism" (p.238) | Irish migrants |
| Cook | 2010 | Looking at older migrant women to England, including some Irish people, and how they accessed citizenship and services. | Qualitative. Life story interviews and focus groups. Data from the Older Women's Lives and Voices Study. | Three themes: The migration histories and entitlements to welfare citizenship; Experiences of accessing welfare rights and services; The importance of resourcing communities. Unsurprisingly, those who had lived in the UK the longest had the best understanding of the welfare system, and had also accrued rights to welfare. No representation of the Irish in the second theme (accessing welfare rights and services), so we must assume they had no difficulties. In the final theme, the importance of community centres, one Irish woman explains how she was refused a council grant because they said she might use it to donate to the IRA. Community centres are not used instead of mainstream services, but rather as a way to enable access to mainstream services. | Irish migrants. |
| Das-Munshi, Becares, Dewey, Stansfeld & Prince | 2010 | To test the ethnic density hypothesis on mental health | Secondary data analysis of the EMPIRIC survey (from 2000) | Prevalence of common mental disorders was highest in Pakistani (20%) and Irish (19%) followed by Indian (18%), Black Caribbean (17%), White British (16%) and Bangladeshi (13%). The ethnic density effect was supported for Bangladeshi and Irish people (and "all ethnic minority groups combined"), whereby they had a significant decline in common mental disorder for every 10% point increase in own-group ethnic density. This is despite the fact that these areas of high ethnic minority density are also socially deprived. However, the theoretical link between social support and experiences of racism did not attenuate the significance of the variables, so this part of the hypothesis was not supported. The web extra tables show that the Irish had significantly higher odds of mental illness if they had experienced racism within the last year, and if they had ever experienced work-place discrimination. Social support was not significant. The middle aged Irish group (35-54) had significantly higher odds of mental illness, compared with the younger age group (16-34). | Parents born in Ireland |
| Karlsen & Nazroo | 2010 | To examine religious differences in health in the 1999 and 2004 HSE, comparing with ethnicity and SES | Secondary data analysis of the HSE (1999 and 2004). Regression. | Christian Irish men had significantly higher odds than Christian White British men of reporting fair/poor/very poor self-rated health, controlling for age. Christian Irish women not statistically significantly different. When men and women combined and controlling for SES, not statistically significantly different. The same pattern was found for having LLTI and for not participating in regular physical activity. Not significantly different for hypertension, diabetes, high body mass index (BMI). In contrast, Christian Irish women had significantly higher odds of having high hip-waist ratio, but men did not; when both genders combined and controlling for SES it was statistically significant. Christian Irish men significantly higher odds than Christian White British men of using tobacco controlling for age; women not significantly different; combining men and women and controlling for SES and age Irish had significantly higher odds than White British. | Uses the HSE, so Irish is born in Ireland or at least one parent born in Ireland. Does not call them White Irish in the paper but does assume they are all white. Several times refers to White (not Irish) people, to make that distinction. |
| Puthussery & Twamley, Macfarlane, Harding & Baron | 2010 | To report on experiences of maternity care among UK-born ethnic minority women | Qualitative in-depth interviews. Grounded theory. | When asked directly, none of the participants thought that their minority ethnic status had made a difference to their care. Low satisfaction was evident when staff were perceived to be unfriendly or impersonal, and when there was no continuity of care, when it was difficult to get information, and when the buildings were austere or unwelcoming. | Ethnicity in terms of 'descent'. All participants UK-born of 'foreign' parents. Irish are included as an ethnic minority group. |
| Tilki, Mulligan, Pratt, Halley & Taylor | 2010 | To argue that the needs of Irish people with dementia in Britain need to be a higher priority to policy makers | Mapping exercise of Irish services for people with dementia / carers. Also some data from semi-structured interviews of people with dementia / carers / staff at Irish services. | The Irish are the oldest ethnic group in Britain and therefore have a higher risk of dementia. Many of this group are in the oldest-old group, more so than any other minority ethnic group. Irish who live in areas with low Irish ethnic density may have difficulty accessing services and be socially isolated. Further reasons for social isolation are widowhood/divorce/separation (likely due to age), or never marrieds (particularly men) without children, lost contact with Irish people back home due to death of parents / infirmity of siblings with age. Many of the older Irish group worked in occupations that did not qualify them for pensions or pay national insurance. Many Irish men have been forced to give up work in middle age due to poor health. Points out the link between social isolation and dementia, already mentioned the high risk of social isolation among the Irish. Reluctance to access services may be caused by past bad experiences of racism/hostility/insensitivity. Irish people have experienced health professionals stereotyping them and assuming heavy alcohol use. There has also been communication barriers because of strong accents or the way English is used by Irish people; some staff make fun of the Irish service users. Services need to understand and be sensitive to the spoken English of Irish people (and also be able to speak/translate for Irish speakers), and also recognise the issues of institutional abuse or historical racism/discrimination experienced by older people. Mentions the importance of wakes & funerals in Irish culture and how they are reassuring and not morbid. Has a few quotes from Irish people with memory problems about how the Irish-specific services help them relax when they hear familiar accents. Services need to implement culturally-sensitive reminiscence activities. Ends by calling for better provision of third sector organisations by the coalition government's Big Society agenda. | Uses the term BME to refer to Irish people in Britain. |
| Ougrin, Banarsee, Dunn-Toroosian & Majeed | 2011 | To understand the rate of suicides in a single London borough to help design action plans. | Record review. Linked with health records. | There were 54 suicides. There was a bimodal distribution in terms of age (peaks at young adulthood and old age). More males than females committed suicide. Seven of the suicides were Irish-born people (13%), and the Irish had a suicide rate of 17.7 per 100,000 per year, which was much higher than the expected suicide rate of 6.8 per 100,000 per year. The seven Irish suicides were all male, average age 68.1, 6 had chronic ill health, 3 had psychiatric illness, 3 had misused alcohol. | Country of birth |
| Kelly & Ciclitira | 2011 | To understand the diet and alcohol use of young Irish men living in London | Qualitative. Braun & Clarke's thematic analysis. | Themes: Lifestyle choices: Lack of time or unwillingness to cook; Being an Irish man, consuming alcohol, and the cost of a healthy diet; Masculine versus feminine food; Dieting as a female activity. The key aspects that the participants related to their Irishness were the focusing on 'spuds' as part of a proper meal (although recognising they are not as nutritious as vegetables), and also the importance of alcohol which took priority over eating properly when socialising. Two of the men said they expected they would eat more healthily if they lived with a girlfriend, which indicates they expected to be looked after by a girlfriend as if she was his 'mammy'. Or possibly, recognising that continuing the weekend all-day drinking lifestyle would not be possible if they had a live-in girlfriend. Implies that Irish men (including older Irish men) who live alone may be at risk of poor diet. | Self-identified ethnicity. White Irish (most), Anglo-Irish (x1), Egyptian-Irish (x1). |
| Smith & Grundy | 2011 | To examine change over time in limiting long-term illness (LLTI) by ethnicity | Secondary data analysis of the ONS Longitudinal Study of England and Wales, 1991 and 2001. Multivariate logistic regression. | Bivariate: Increase in LLTI between 1991 and 2001 for all groups, In 2001 White Irish significantly higher percentage of LLTI than the White British group, controlling for age and sex (but so were Indian, Pakistani, Bangladeshi, and Chinese in 2001). The White Irish significantly more likely to have LLTI than White British in 2001 but not in 1991, indicating a widening of the disparity over time. Contrary to the assumptions about greater length of stay leading to a reduction in inequalities, for this specific ethnic group. Black Caribbean was in the other direction (significantly different in 1991 but not in 2001). Multivariate: analysed separately for males and females. White Irish men significantly more likely to have LLTI in 2001 compared with 1991 even when adjusted for age, SES, length of stay. For women, odds of LLTI in 2001 compared with 1991 significantly higher in all models, but not quite as high as for the Irish men. Note that this analysis did not compare ethnic groups against each other. Controlling for SES did not attenuate the outcomes. Conclusion recognises that the determinants of ill health may be different between different ethnic / migrant groups, and may also be different with different generations. | White Irish. |
| Twamley, Puthussery, Harding, Baron & Macfarlane | 2011 | To understand new mothers' experiences of feeding their babies, focusing on UK-born ethnic minority women | Individual interviews, qualitative. Grounded Theory. | The mothers recognised the health benefits of breast feeding, but reported the logistical problems. Breast feeding takes a long time and mothers can't multi-task while it is happening. Bottle feeding is faster, and someone else can hold the baby/bottle which gives the mother free time to go out/ do chores/ rest. Many of the South Asian women reported pressure from grandparents to introduce bottle feeding because of a perception that breast milk wasn't sufficient (either in nutrients or in volume) to feed an infant. The Irish women were the only ones who intended to feed formula from the start; there was some embarrassment at the idea of breast feeding but also a common understanding among their family and friends that formula was normal or standard. There was no argument with grandparents about this, perhaps because the Irish women had internalised the idea that formula was normal and fine. | UK-born ethnic minority women, includes Irish. |
| Allnock, Radford, Bunting, Price, Morgan-Klein, Ellis & Stafford | 2012 | To understand need for (and unmet need) services to support children who have been sexually abused | Mapping of services, cross-referenced with data from an NSPCC survey about numbers of children who have been sexually abused. Questionnaires to service managers. Interviews with service managers/commissioners. | The findings estimate that there is a shortfall of 54,220 spaces. That means, 54,220 children or young people who wish to use a service cannot do so. This does not include the children/young people who do not wish to use services even though they have experienced sexual abuse. A very small number of the referrals were from ethnic groups other than White British. White Irish are reported separately, but the 'mean' figure is 1.1, while mixed is 1.6, Asian is 1.2 and Black is 1.1. White British mean is 28. | White Irish. |
| Bhopal, Bansal, Steiner, Brewster & Scottish health and Ethnicity Linkage Study | 2012 | To examine ethnic differences in Scotland in cancer rates | Scottish Health and Ethnicity Linkage Study, linked to 2001 Census | White Scottish had higher rates of all cancers than any other ethnic group, apart from 'any mixed background'. White Irish had similar rates to White Scottish of lung cancer. White Irish men and White Scottish men had the highest rates of colorectal cancer. White Irish women had lower directly age standardised rates and ratios (DASRRs) than White Scottish women for breast cancer. White Irish men had lower DASRR than White Scottish men for prostate cancer. In many cases the 'visible' minority groups had lower cancer rates than the White Scottish. | White Irish. |
| Cox, Marland & York | 2012 | To examine records from asylums in late 1800s Lancashire with a focus on Irish patients. Also to discuss public discourses about the Irish. | Historical study. Asylum records review. | The asylum was sometimes a final destination for those who entered the workhouse due to poverty. Many were admitted to the asylum due to violence and unmanageable behaviour. The Irish were perceived as a very mobile people ("tramping"), having moved around Britain a lot, while others had come via America or had been former soldiers. As a result they had no roots or social network, and so were more likely than others to be long-stay patients at the asylums. Another cause of mental illness among the Irish in Lancashire was thought to be related to the stresses of migration, and particularly the unfulfilled ambition to reach America or Australia. The records do not link the famine experience to the rates of mental illness; this is despite the fact that the poor physical condition and general under-nourishment of the Irish inmates is noted. The Irish were more likely to be diagnosed with mania than the rest of the patients, which the authors argue is due to stereotyping of the Irish as "excitable, bellicose and willful" (p.512). The authors argue that over time, the link between Irishness and physical and mental infirmity was solidified, and no longer related to the famine. It was described as a "degenerative trait" by this stage (p.513). There is mention of "sectarian violence" in Lancashire at the time. Public attitudes towards the Irish got worse. Irish people were less likely than other inmates to recover, and recovery rates were worse for those who were single or widowed. By the 1870s the Irish inmates' facial features are described as being linked to their illness and overall degeneracy. A similarity to a monkey is mentioned, which is clearly related to the popular depictions of the Irish as simian or having a prognathis jaw. | Born in Ireland, 1800s, so still part of United Kingdom |
| Emerson | 2012 | To understand the relationship between childhood intellectual and developmental disability and ethnicity and social deprivation | Data from the 'School Census' of Spring 2008. Multilevel logistic regression. | Controlling for all the variables in the model, Gypsy/Romany and Irish Traveller children had higher rates of moderate learning difficulties and severe learning difficulties (SLD) (but not 'profound and multiple learning difficulties' (PMLD) or autistic spectrum disorder (ASD)). Children eligible for free school meals were more likely to have an intellectual or developmental disability (IDD), controlling for other variables. Rates of identification of IDDs were lower in most minority groups, although rates of severe learning difficulties and profound and multiple learning difficulties were higher among Pakistani children, and PMLD among Bangladeshi children. Irish children (not Travellers) were significantly less likely to have moderate learning disabilities than the White British children, but not significantly different in SLD, PMLD, or ASD. | Irish (not White Irish), Traveller of Irish heritage |
| Hurcombe, Bayley, Thickett & Thom | 2012 | To understand the use of alcohol among Irish Travellers | Oral life history approach | Alcohol was seen as a normal part of celebratory events, or more regular moderate drinking. Girls were not supposed to drink until they were married. This was seen as part of keeping them 'pure' and suitable for marriage. One reason for girls not staying in school was to avoid mixing with the wrong type of girls who would lead them into bad behaviours. Women were allowed to drink but didn't drink much because they were generally busy with caregiving or domestic duties. Men might go out to drink, but this would be moderate, as men were expected to work and provide financially for their families. Young men were thought to behave differently to the previous generation, and were more likely to go out into 'discos' in town and mix with other people, and drink more. The change to a more settled lifestyle meant the close-knit nature of life on the halting sites was changing; they didn't know everyone in a housing estate so they didn't feel safe to let their children run around unsupervised. Socialising with other travellers was more difficult, but people still came together in times of crisis. | Irish Travellers |
| Mangalore & Knapp | 2012 | To assess whether ethnic group disparities in common mental illness is related to socio-economic position | Secondary data analysis of the EMPIRIC survey (from 2000). Examined income distribution within ethnic groups. | Descriptive level: Irish had the highest percentage of 'cases' of common mental illness (21%) and also had the equal highest mean number of Clinical Interview Schedule-Revised (CIS-R) symptoms (tied with Pakistani). Irish had the second-highest mean income, after White (not Irish). Pakistani and Bangladeshi groups had the lowest incomes. Looking at the within-group analyses, the Irish had the highest 'within group inequality', meaning that the inequality in mental health caused by income was greatest in this ethnic group. This was standardised for age and gender. This implies greater disadvantage for the poorer Irish compared with the richer Irish, and to a greater extent than other ethnic groups. However the between groups analysis shows that both the White and Irish groups have a "concentration of ill-health among the higher income groups" (p.354), which is contrary to the usual expectation that low income is related to poorer health. | Irish includes born in Ireland or a parent born in Ireland (EMPIRIC survey). |
| Maynard, Rosato, Teyhan & Harding | 2012 | To assess change over time in rates of suicide among migrants to Britain | Secondary analysis of death records and Censuses between 1979 and 2003. | Men from the Republic of Ireland had higher suicide rates than men born in England & Wales; men from the Republic of Ireland also had an increase in suicide rate over time. The highest suicide rate in 1979-1983 among men was RoI followed by Scottish; in 1989-1993 was the Northern Irish followed by RoI; in 1999-2003 was the Jamaicans, followed by RoI. For women from Republic of Ireland, suicide rates were higher than England & Wales born women. Suicide rates higher for men than for women, as is typical. For women, highest suicide rates in 1979-1983 were RoI followed by Scottish; in 1989-1993 was RoI followed by India, for 1999-2003 was RoI followed by Scottish. | Country of birth. RoI or NI. |
| Smith, Kelly & Nazroo | 2012 | To examine the relationship between ethnicity and acculturation (generation) on obesity | Secondary data analysis of the 1999 and 2004 Health Survey for England | Irish men in both first and second generation significantly higher odds of being a current smoker compared to White British (WB) men; Irish women not significantly different for smoking. Irish people in first generation significantly lower odds of having drunk alcohol in the past week compared to WB; second generation not significant. Irish people in first and second generation significantly lower odds of eating crisps/chocolate/biscuits 6+ times a week compared to WB. First generation Irish people significantly higher odds of eating fried food 3+ times a week; second generation not significantly different. Irish people's odds of being obese not significantly different from WB reference group in either first or second generation. Second generation Irish not significantly different odds of obesity compared to WB even when adjusting for health behaviour, age, sex, and SES. Irish were generally quite well-off; most Irish people were in the highest income quintile (different from WB); first and second generation had similar SES (although second gen slightly more likely to be in second social class than first, while first gen more likely to be in first than second), which differs from most minority groups where there tends to be upward social mobility between generations. For most of the minority groups included, odds of obesity reduced in second generation in line with the acculturation hypothesis (get closer to White British population with subsequent generations), but Black Caribbean bucked the trend with second generation significantly higher odds than WB of being obese. | Irish (not White Irish), from HSE. Comparison group is just 'White', implies Irish group are not White. |
| Van Hout & Staniewicz | 2012 | To argue that the needs of Roma and Irish Travellers in terms of housing and health need more attention | Desk research, interviews with Roma and Travellers, and with advocates, government and other stakeholders. Also some case studies. This was included in an EU report. | Literature review brings together some studies on the health of Irish Travellers in Britain and Ireland. Mentions that the age structure of the Irish Travellers is similar to that of a developing country with high fertility and low numbers of older people. There is also high infant mortality and congenital disease. Respiratory illnesses like asthma and chest infections are common, as are issues related to hygiene like diarrhoea and infections. Heart disease and disability are also mentioned. Suicide levels are high and argued to be related to the psychological pressure to adopt a settled lifestyle. Access to health services is compromised by the difficulty in finding suitable halting sites. | Irish Travellers |
| Baker, Mitchell, Lawson & Pell | 2013 | Is screening for cardiovascular risk among different ethnic groups cost-effective? | Secondary analysis of the Health Survey for England 2003 and 2004 | Mass screening would be least cost-effective in the Irish and Black Caribbean groups (because you would have to screen more people in these groups to find a high risk individual). Targeted screening (focusing on the most deprived quintile) was more cost-effective in all minority groups in comparison with the general population. | Irish is compared with 'White' and sometimes 'general population'. Implies authors do not see the Irish as white. |
| Becares | 2013 | Short briefing paper (from Manchester Centre on Dynamics of Ethnicity) that reports on ethnic health inequalities 1991 to 2011 | Secondary analysis of the Census, 1991-2011 | In 2011: The worst health (in comparison with White British) is found among the White Gypsy or Irish Traveller group (both men and women) - it is twice the rate of LLTI/D among White British. White Irish men and Mixed White-Black Caribbean men also have a higher rate of LLTI than White British men. None of the other ethnic groups have a higher rate. Among women, as well as White Gypsy or Irish Traveller, Pakistani, Bangladeshi, Arab, Black Caribbean and Other Black women also have higher rates of LLTI than WB, but White Irish women have a slightly lower rate. In 2001: White Irish men had the highest rate of LLTI, followed by Bangladeshi, Pakistani, Mixed White-Black Caribbean and Black Caribbean men (in comparison with WB). Among women, the Pakistanis had the worst health, followed by Bangladeshi, Black Caribbean, Indian, White Irish and Other Black women (in comparison with WB). So, over time, the White Irish men were consistently in poor health (LLTI) but are no longer in the worst health (that is White Gypsy or Irish Traveller), whereas the White Irish women seem to have improved their health over the decade 2001-2011. So, the very bad health of the White Irish men in 2001 may have been partially, but not fully, reflecting the White Gypsy or Irish Traveller. | White Irish. White Gypsy or Irish Traveller. |
| Bhopal, Humphry & Fischbacher | 2013 | To assess change over time in cardiovascular risk factors among ethnic groups | Secondary analysis of the Health Survey for England 1999 and 2004 | Absolute numbers (not controlling for age): Change over time: Irish men worsened over time on waist-hip ratio, but improved on diabetes, physical activity, high-density lipoprotein (HDL), triglicerides, and smoking. No change on BMI, total cholesterol, or blood pressure. Irish women worsened over time on waist-hip ratio, improved on physical activity, triglycerides and smoking. No change on diabetes, total cholesterol, blood pressure, BMI, HDL. The groups that had a strong pattern of worsening over time were Pakistani and Bangladeshi women, Black Caribbean, Indian and Pakistani men. Relative numbers (controlling for age): Irish men not worse on any indicator, better on physical activity, smoking, triglycerides, waist-hip ratio, and diabetes, no difference in HDL, total cholesterol, BP, or obesity compared with earlier wave. Irish women: worse over time in HDL and physical activity. Better over time on obesity, triglycerides, diabetes and waist-hip ratio. No change on smoking, total cholesterol or blood pressure. Overall Bangladeshi and Pakistani men and women had the most number of declines in health indicators over time. Overall conclusion regarding the Irish is that there has been an improvement in risk factors over time (contrary to the other minority ethnic groups), but the authors caution that the finding needs validation. | This article studied "the major ethnic groups in England" (p.1) and included the Irish as one of these. Later it mentions that the HSE included "the largest non-White ethnic minority populations" (p.2), so seems to be classing the Irish as non-White. |
| Das-Munshi, Clark, Dewey, Leavey, Stansfeld & Prince | 2013 | To use a lifecourse perspective to understand the poor health of second-generation Irish people in Britain | Secondary data analysis of the National Child Development Survey | 2nd generation Irish children lived in more socially adverse circumstances than the rest of the cohort, which continued into early adulthood (23), started to tail off at age 33, and by midlife (42, 44/45) had become equivalent to the rest of the cohort. 2nd generation Irish were more likely than the rest of the cohort to have depression at age 23 and later in their lifecourse (with a bit of a reduction in the size of the risk). No difference in fair/poor self-rated health at age 23 or 33, but this seemed to become more different at age 44/45. Including childhood material adversity and family adversity into the models attenuated the risk of common mental disorder and poor self-rated health in midlife, but health-related behaviours and earlier psychological health/self-rated health did not attenuate the risk. The authors conclude that childhood/early adulthood is a critical period whereby adversity at that time can have long-term health implications. This is an explanation for why there can be health differences in midlife (and presumably later) even when socio-economic status with the host population might have equalised. | Second generation Irish (children born in Great Britain who had at least one parent born in RoI or NI) |
| Delaney, Fernihough & Smith | 2013 | Compares the health status of different waves of migrants from Ireland to Britain, and argues that the poorest health was in those born between 1920 and 1960. | Secondary analysis of the Health Survey for England 1999 and 2004; Living in Ireland Survey wave 7 2000; The Irish Longitudinal Study on Ageing (TILDA) 2011. | Data from TILDA show that 11.5% of people (aged 50+) experienced abuse in childhood (7% sexual, 8% physical); rates of abuse in childhood were 50% higher among those who had emigrated and returned compared to those who had never left Ireland. There are no figures on the rates of childhood abuse among Irish emigrants who did not return, but there are indications that the rates are even higher in this group. There is a discussion of abuse in industrial schools based on the Ryan Commission report, and an extrapolation of figures to suggest that survivors of industrial schools make up 6.3% of the migrants from 1927 to 1970. Graph on p.2021 showing self-rated bad health in the three groups: Irish in Ireland, English in England, Irish in England, with very noticeable worse health for the Irish in England born 1921-1960, but tailing off/equalising for those born 1961 onwards. Similar health for those born pre 1921. The same treatment is given to General Health Questionnaire (GHQ) scores (mental health), which show that Irish people born 1931-1950 have worse mental health than the other two groups, but not much difference for later-born people between the three groups. Irish born pre 1921 have the best mental health out of the three groups. Regression analyses show the Irish-born between 1921-1960 and now living in England have worse health on some measures than the English (self-rated health, cardiovascular conditions), and also than the Irish living in Ireland (self-rated health, mental illness especially for men). The migrants who were born between 1921-1950 were less educated than their contemporaries who stayed at home; in contrast the migrants born between 1961-1980 are more educated than their contemporaries who stayed at home. Drinking data (from HSE) shows the Irish in England drink more heavily than the White English, and also that there is a larger proportion of the Irish who have stopped drinking. Overall conclusion is that the cause of the poor health of the Irish in Britain is that the emigrants (born 1921-1960) were less healthy, less skilled, and less educated to start with (and likely to have a history of abuse), and they brought their poor health and poor life chances with them when they emigrated. It was not a result of a negative environment when they arrived (e.g. anti-Irish sentiment due to the Troubles). | Irish-born living in England |
| Leavey & Eliacin | 2013 | Book chapter focusing on mental health among Irish migrants who have returned to live in Ireland | Literature review | Rationales for higher rates of mental illness among Irish migrants to Britain: **An undeveloped sense of ethnic identity** (i) The fact that Ireland used to be a British colony means that the Irish in Britain cannot "develop and celebrate" a "positive ethnic identity" (p.199), (ii) their ethnic identity is therefore "fragile" (p.200), and (iii) they are not recognised as an ethnic (minority) group so they don't have any "political redress" (or didn't at the time that was written, late 1990s) (p.200), (iv) if they don't have a strong sense of their own ethnic identity then they do not have the protective effects this offers (and links with the ethnic density effect, which could reinforce positive ethnic identity). T**he unplanned nature of migration** (i) Ireland is very close to Britain and easy and relatively cheap to get to, (ii) the common travel area means there are few bureaucratic barriers to emigrating, (iii) people can therefore make a spontaneous decision to travel without any serious plans for settling down or finding a social network, (iv) people can assume they can return home whenever they want to, (v) this leads to being marginalised in Britain with no social networks and few resources to draw on in case of emergency. **Return migrants** have to go through a process of acculturation when they come 'home', and they may not have anticipated this. They may feel out-of-step with their 'home' culture, have lost their social networks from the new country, and may feel isolated as a result of how they have changed or their disappointment at how their 'home' is no longer the same. | Irish born. Refers to the Irish in Britain as "a white ethnic group", so not considering Irish people of colour. |
| Lievesley | 2013 | Centre for Policy on Ageing document, giving evidence to the Birmingham Policy Commission on Healthy Ageing in the 21st Century | Population projections into the future of ethnic minority groups in England & Wales, based on 2001 Census data. Also draws on EMPIRIC and HSE 2004. | Older Irish men and women are more likely to be smokers than the general population. For older women, this is the opposite pattern than most BME groups. For older men, several BME groups are more likely than the general population to be smokers. When reporting data from EMPIRIC, Irish not included in the figures on psychosis for older people. Irish do have higher levels of "feeling downhearted and low" (aged 55-74) compared with White British, but lower than Black Caribbean, Bangladeshi, Indian, Pakistani. Presents the population projections for older people from BME groups up to 2051, also care home population of BME groups up to 2051, and projected cases of dementia for BME groups up to 2051. | Irish, White Irish |
| Aspinall & Mitton | 2014 | To examine smoking rates among different ethnic/migrant groups in the UK, with a focus on new migrant groups | Secondary analysis of the Integrated Household Survey (2009/10-2011/12) and GP Patient Survey (2012). | White Irish men (15%) have higher rates of being a regular smoker than White British men (13%); White Irish women are slightly lower (9%) than White British women (10%). White Irish men and women have higher rates of being former smokers compared to White British men and women, and lower rates of being never smokers. White Gypsy or Irish Traveller men (39%) and women (35%) have very high rates of being regular smokers compared to White British men (13%) & women (10%). In fact, the White Gypsy or Irish Traveller group have the highest rate of being regular smokers of all ethnic groups included in the survey. Most of the other ethnic groups have similar or lower rates of smoking compared to White British, apart from Bangladeshi men (18%) and Arab men (17%). When looking at country of birth data, rates were very high among migrants from Eastern Europe. The relationship between SES and smoking is variable depending on ethnic group. | White Irish, White Gypsy or Irish Traveller |
| Bansal, Chalmers, Fischbacher, Steiner, Bhopal & SHELS | 2014 | To report on the health of mothers and babies from different ethnic groups in Scotland | Scottish Health and Ethnicity Linkage Study (SHELS), linked to 2001 Census and hospital records. | On most indicators, white Irish women/babies tended to be healthier than white Scottish women/babies. However, the White Irish had low breast feeding rates and slightly high caesarean rates. | White Irish. Note: They use a 'country of birth' category of UK/non-UK, but their UK category includes anyone born in the British Isles, including those born in the Republic of Ireland (not part of the UK). |
| Bhopal, Cezard, Bansal, Ward, Bhala & SHELS | 2014 | To report on ethnic group differences in gastrointestinal diseases in Scotland | Scottish Health and Ethnicity Linkage Study, linked to 2001 Census, hospital and death records. | White Irish men and women had higher risks of appendicitis and diverticular disease compared with White Scottish men and women. White Irish men had lower risk of ulcerative colitis compared with White Scottish men, when controlling for age, index of multiple deprivation and country of birth. | White Irish |
| Das-Munshi, Clark, Dewey, Leavey, Stansfeld & Prince | 2014 | To assess whether socio-economic disadvantage can account for mental illness among second-generation Irish in Britain | Secondary data analysis of the National Childhood Development Survey (NCDS) (children born 1958) and the 1970 Birth Cohort (BCS). | Six percent of the participants in the two birth cohorts were second-generation Irish. The second-generation Irish children experienced greater childhood adversity compared with British children. Mothers of Irish second-generation children had indicators of poorer health (chronic conditions, mental illness), but these attenuated when controlling for material conditions. Irish second-generation children had more psychological symptoms than British children. Controlling for material hardship attenuated this relationship in the NCDS at ages 7 and 16. In the NCDS at age 11 the relationship was not attenuated. In the BCS, Irish children had better mental health than the rest of the cohort after controlling for material hardship and mother's psychological health. | British-born children of Irish-born parents who migrated to Britain |
| Das-Munshi, Leavey, Stansfeld & Prince | 2014 | To assess whether socio-economic disadvantage accounts for alcohol and tobacco use among second-generation Irish in Britain | Secondary data analysis of the National Childhood Development Survey (children born 1958). | Irish men had significantly higher odds of harmful (more than 50 units a week) alcohol use at age 23 compared with rest of cohort; Irish men and women at age 33 had significantly higher odds of hazardous (yes/no answers about feelings on own alcohol use) alcohol use. In both cases the finding was non-significant at age 42. Second generation Irish women significantly higher odds of being a smoker in 1991 and 2002; Irish men not significantly different from rest of cohort. Second-generation Irish children more likely to experience childhood material and social disadvantage, but these issues become similar to the rest of the cohort by mid-life. This is an indicator of social mobility within the generation. | British-born children of Irish-born parents who migrated to Britain |
| Das-Munshi, Castro-Costa, Dewey, Nazroo & Prince | 2014 | To validate the Clinical Interview Schedule-Revised (CIS-R) with different ethnic/cultural groups | Secondary data analysis of the EMPIRIC survey 2000. Principal components factor analysis. Ordered logistic regression. | The CIS-R has a list of 14 symptoms of psychological distress; the Irish and White British reported similar levels of symptoms. This was argued to be due to sharing a common language and therefore similar ways of expressing distress. There was greater variety in the other ethnic groups, including the anticipated higher rate of reporting somatic symptoms among Asian groups. The factor analysis showed the measure was valid in different ethnic groups, although there were clear differences between them. | Irish |
| Jack, Moller, Robson & Davies | 2014 | To examine ethnic group differences in use of breast cancer screening opportunities in London | Examination of health records. Logistic regression. | White Irish women significantly less likely than White British women to attend either the first or the routine later appointments (controlling for age, location, and socio-economic deprivation). White Irish significantly more likely than some groups (e.g. Mixed White & Black African, Bangladeshi, or Black African women). Results differed depending on the part of London the women were from: White Irish women from North London, Barking/Havering/Redbridge/Brentwood, South East London, and 'All London' significantly less likely to attend first appointment compared to White British women, whereas no significant difference for West of London, Central & East London, or South West London. For routine later appointments, White Irish women in most parts of London significantly less likely than White British women to attend, except in West London and South West London (no significant difference). The ethnic group differences are not accounted for by age or socio-economic deprivation. | White Irish |
| Koffman, King Ho, Davies, Gao & Higginson | 2014 | To look at place of death from cancer in London across different ethnic groups | Examination of death records. Poisson regression. | Comparing with UK-born people, and controlling for age, sex, marital status, cancer type, and index of deprivation, Irish-born people were significantly more likely to die in their own home or in a nursing/residential home, and significantly less likely to die in a hospital. No significant difference for hospice. The Irish places of death seem to have a specific pattern that does not match that of any of the other 'country/region' groups studied. For example, Caribbean people significantly less likely than UK-born to die in their own homes, and European-born significantly less likely than UK-born to die in a communal establishment. | Country of birth (RoI). |
| Mindell, Knott, Ng Fat, Roth, Manor, Soskolne & Daoud | 2014 | To examine potential reasons for ethnic differences in health using the HSE | Secondary data analysis of the Health Survey for England 2003-2006. Logistic regression. | Poor self-rated health: Irish men not significantly different from White British men, controlling for all variables. Irish women had significantly lower odds of poor self-rated health compared with White British women, but this became non-significant once 'community characteristics' were controlled for. Limiting long-standing illness (LLI): Irish men not significantly different from White British men. Irish women non**-**significantly lower odds of LLI compared with WB women, and this became significant after controlling for demographic factors and also psychosocial factors. Health was worst for Pakistani and Bangladeshi women, and also Black Caribbean women. The Chinese and Black Africans had the best health. Many of the health inequalities were better explained by socio-economic disparities, and the authors argue that it is essential to include a wide range of socio-economic indicators in order to adequately identify the kind of disadvantage migrant/ethnic groups experience. | Irish |
| Ryan, D'Angelo, Puniskis & Kaye | 2014 | To analyse the 2011 Census data with a focus on Irish people in London | Secondary data analysis of the 2011 Census | The numbers of people living in England but born in either RoI or NI have reduced between 2001 and 2011. In London, Irish-born people mostly live in the North West and West, specifically Brent, Islington, and Hammersmith & Fulham. In 2011 the White Irish is the 9th largest ethnic group in London (out of 18). White Irish have a very old age structure. Comparing ethnicity with country of birth; of those born in the Republic of Ireland, 1.3% (1678) report having a mixed/multiple ethnic group, 1.5% (1948) identify as Asian/Asian British and 2.4% (3074) identify as Black/Black British. The majority (28.4%) arrived before 1961, with large numbers also arriving between 1961-1970 (18.1%) and 1981-1990 (15.1%). Dip 1971-1980 (9.3%). As is common for migration, the majority of people migrated at young working age (16-24 or 25-34), and many arriving as children (0-15). Fewer arrived after the age of 35. Education: White Irish (26.1%) and White Gypsy or Irish Traveler (54.3%) are the groups most likely to have no qualifications at all (compared with White British, White Other, Mixed, Asian, Black and Other). White Irish (37.3%) have almost identical percentage with a degree as White British (37.2%), and this is similar to Asian (39.7%), Mixed, (34.6%), Black (33.5%) and Other (33.9%), but lower than White Other (42.5%) and much higher than White Gypsy or Irish Traveller (13.3%). LLTI/D: Highest percentages overall in Gypsy or Traveller (25.0%) and White Irish (23.9%). When examining by age, the White Irish have greater percentages with LLTI/D at ages 35-49 and 50-64 than White British (but lower than Mixed, Asian, and Other; higher than White Other and Black), and slightly less at age 65+ than WB. However, Gypsy or Traveller have higher percentages at every age range. White Irish largest percentage of people aged 65+ living alone in London out of all ethnic groups. | Irish-born (RoI, NI), White Irish, White Gypsy or Irish Traveller |
| Barry, Laverty, Majeed & Millett | 2015 | To determine whether ethnic group differences in admission to hospital for alcohol related issues is patterned by geographical location | Hospital records study (Hospital Episode Statistics). Descriptive analysis only. | White Irish had high rates of hospital admission for alcohol-related issues in the country as a whole, but there was geographical variation. Higher in London compared with other regions (in fact, White Irish in London had highest rates of all groups and all regions). There was also some variation when comparing whether alcohol was the primary or secondary reason for admission - when looking at the secondary reason the White British actually had the highest rates in the North of England. North of England generally had higher rates than the South of England (not including London). Pakistani & Bangladeshi lowest overall. Alcohol is a significant issue for White Irish people living in London in 2010/11. No controls for age. | White Irish |
| Bhopal, Steiner, Cezard, Bansal, Fischbacher, Simpson, Douglas, Sheikh & SHELS researchers | 2015 | To examine ethnic group differences in respiratory-related cases of hospitalisation, readmission, and death. | Scottish Health and Ethnicity Linkage Study. Hospital records and death records 2001-2010 linked with Census 2001 data | White Irish men had significantly higher relative risk of first all-respiratory disease hospitalisation or death compared with White Scottish men when controlling for age only. Once the index of deprivation was included the difference became non-significant, and was still non-significant when controlling for country of birth. White Irish women were not significantly different from White Scottish women on the same measure. When looking at age-adjusted rates for first chronic obstructive pulmonary disease (COPD) event or death in those aged 40+, White Irish men and women had significantly higher relative risks compared with their White Scottish counterparts controlling for age, index of multiple deprivation, and country of birth (each in separate models). When combining all three covariates into one model, however, there was no significant difference between White Irish and White Scottish men and women. When looking at deaths from respiratory causes following hospitalisation for any COPD disorder, White Irish men had no significant difference from White Scottish men, and White Scottish women actually had significantly lower hazard ratios of death than White Scottish women. | White Irish |
| Condon & Salmon | 2015 | To understand the views of Gypsy, Traveller & Roma women about breastfeeding | Qualitative. Semi-structured interviews. | Key themes were (i) centrality of the family, (ii) beliefs and traditions related to culture, and (iii) travelling lifestyle. The women spoke about a tradition of large families and older siblings taking care of younger siblings. They therefore considered that they had learned how to take care of children already and did not need advice from a professional. They viewed knowledge from experience more highly than knowledge from books, and considered that health visitors who had never had children were not able to give good advice because it only came from books. Health visitors often gave written materials, but many of the women couldn't read (some at all, some in English). Roma women saw breast feeding as normal, whereas English Gypsies and Irish travellers more commonly used bottle feeding, and considered breast feeding to be a thing that settled people ('gauje people') would do. Breast feeding was seen as the same as exposing yourself, and therefore not appropriate behaviour. The lack of opportunities for privacy when living in a caravan (people in and out all the time) meant it was difficult to get private time for breastfeeding. | Irish Traveller |
| Millard, Raab, Lewsey, Eaglesham, Craig, Ralston & McCartney | 2015 | To look at mortality differences by religion, age, ethnicity, disability, and gender in Scotland | Scottish Longitudinal Study (census linkage from 1991, 2001, 2011). Hazard ratios and cox proportional hazard models. Relative index of inequality (RII) | People raised Roman Catholic were more likely to live in deprived areas than those raised in the Church of Scotland. Hazard ratios (higher mortality rates) were higher for Roman Catholics and 'no religion' within each deprivation decile and social class group. | White Irish and White Other collapsed in with White Scottish and Other British into a single White group. White Irish are hidden because they were collapsed into a single White group. Roman Catholic religion, however, can be seen as an imperfect proxy of Irishness in this Scottish dataset (although some will be Polish or of other origin). |
| Rao, Schofield & Ashworth | 2015 | To examine links between ethnicity an socio-economic factors on alcohol use among older people | Records from primary care database. Regression. | Looking at alcohol use, the Irish group was the only ethnic group that was a significant positive predictor of alcohol use compared with the 'white' group; all the other ethnic groups were significant predictors of not drinking compared with the 'white' group. The Irish group was also a significant predictor of unsafe drinking compared to the white group; None of the other ethnic groups were significantly different from the white group on unsafe drinking. These analyses controlled for sex, age, index of multiple deprivation, and comorbidity. Index of multiple deprivation was a significant negative predictor of unsafe drinking; that is, the lower the social deprivation the more likely to drink unsafely. Being younger-old was significantly related to drinking/unsafe drinking compared to being older-old. | Irish. Includes the Irish as an ethnic minority group. One of the comparison groups is "white", so the Irish are not considered 'white' in this study. |
| Sharpe, Cezard, Bansal, Bhopal & Brewster | 2015 | To understand place of death from cancer in Scotland and whether it varies by ethnicity. | Secondary data analysis of records of Census, hospital episode statistics, mortality data. | In the multivariate analysis, White Irish significantly more likely than White Scottish to die at home (same outcome for Other White British). The authors linked this to living in a rural area, and therefore the issue may have been proximity to hospice rather than behavioural / attitudinal issues. | White Irish |
| Wallace & Kulu | 2015 | To examine whether there is a healthy migrant effect of migrants to Britain by looking at mortality rates and causes of death. | Survival analysis of the ONS Longitudinal Study. | Men and women from the Republic of Ireland have high mortality from all causes compared with UK-born men and women, even when controlling for SES, marital status, and area of residence type. When looking at death from cardiovascular disease, men and women have higher mortality than the UK born, but only women still have higher cardiovascular disease (CVD) mortality after SES is controlled for. Death from cancer is initially higher for RoI men and women, but this becomes non-significant after control variables added. Death from respiratory disease was significantly higher among RoI men and women even after controlling for SES. No significant difference in deaths from infectious diseases between RoI and UK born. When looking at death from "other causes" and controlling for SES, mortality rates are lower for RoI born men compared with UK-born (women not significantly different). The explanation given for the high mortality among RoI-born (and also NI born and Scotland born) is that the ease of migration for these groups means people do not have to self-select for health (i.e. there is no imperative for the healthy migrant effect to operate). The authors assume that immigrants from RoI will have "extensive support networks" in the new country (p.219) and therefore not experience the "physical and psychological challenges associated with migration" (p.219). | Country of birth (RoI) |
| Wohland, Rees, Nazroo & Jagger | 2015 | To compare disability free life expectancy and healthy life expectancy among different ethnic groups | Secondary analysis of the 2001 Census. Two methods of producing disability-free life expectancy (DFLE) and healthy life expectancy (HLE), called Standardised Illness Ratio (SIR) and Geographically Weighted Method (GWM). | Irish men had about the same number of years of life expectancy at birth compared to White British men; Irish women had on average two years more life expectancy at birth compared to White British women. Irish men had fewer number of years of disability-free life expectancy compared with White British men, and White Irish women had slightly more (but largely the same) number of years compared with White British women. | White Irish |
| Bhala, Cezard, Ward, Bhansal, Bhopal, SHELS | 2016 | To compare hospital admissions and mortality related to alcohol and liver disease in Scotland, and look for ethnic group differences | Secondary analysis. Risk Ratios. | White Irish men and women had significantly higher risk ratios of first alcohol related disease and first alcoholic liver disease compared with White Scottish men and women. White Irish women had no significant differences on all liver disease, compared with White Scottish, but White Irish men had very slightly significantly greater risk ratio of all liver disease compared with White Scottish reference group. Controlling for age, Scottish Index of Multiple Deprivation (SIMD), and country of birth made the difference between White Irish men and White Scottish men on risk of alcoholic liver disease hospitalisation or death non-significant. For White Irish women the association became non-significant after controlling for age and country of birth. For alcohol-related diseases, the association did not attenuate into non-significance for White Irish men and women even when controlling for all variables. White Irish men and women had the highest risk of alcoholic liver disease out of all ethnic groups. Seriously at risk. | White Irish. |
| Das-Munshi, Ashworth, Gaughran, Hull, Mrogan, Nazroo, Roberts, Rose, Schofield, Stewart, Thornicroft & Prince | 2016 | This paper is a protocol for a study called E-CHASM, which aims to look at cardiovascular disease and ethnicity among people with severe mental illness. | Secondary analysis of patient records. Qualitative interviews and focus groups with clinicians, service users, and carers. | This is a protocol for a study, so the findings here are only the sample characteristics. Irish are included as "Irish" rather than "White Irish" and discussed as one of a number of "ethnic minority groups". | Includes the Irish as an "ethnic minority group". |
| Evandrou, Falkingham, Feng, Vlachantoni | 2016 | To use up-to-date data to review if their previous findings on ethnic inequalities in health are still present | Secondary data analysis of the first wave of Understanding Society 2009-2011 | In multiple regression analysis controlling for age, sex, length of time in UK, income quintile, and deprivation quartile, the Irish are not significantly different from the White British in terms of limiting health condition or self-rated poor health. For women, at the bivariate level the Bangladeshi group have the highest rates of limiting health condition at age 75+ followed by the Indian and then Pakistani, and the Irish have the fourth-highest rates of limiting health-condition. Irish rates of poor health are at the lower end of the range of ethnicities. | Irish (not 'White Irish'). |
| Gruer, Cezard, Clark, Douglas, Steiner, Milard, Buchanan, Vittal Katikireddi, Sheikh & Bhopal | 2016 | To examine ethnic group differences in life expectancy in Scotland | Linkage of death records 2001-2004 with 2001 Census. Part of the SHELS study. | White Scottish men and women had low life expectancy, in most cases lower than any other ethnic group (apart from Any Mixed Background). White Irish men were very similar to White Scottish, but had a shorter life expectancy than any other ethnic group (apart from Any Mixed Background). White Irish women had a higher life expectancy than White Scottish women and Any Mixed Background women, but a shorter life expectancy than women of all other groups. | White Irish |
| Jackson, C., Dyson, L., Bedford, H., Cheater, F. M., Condon, L., Crocker, A., Emslie, C., Ireland, L., Kemsley, P., Kerr, S., Lewis, H. J., Mytton, J., Overend, K., Redsell, S., Richardson, Z., Shepherd, C., & Smith, L. | 2016 | Health Technology Assessment report on vaccine uptake among Gypsies, Roma, and Travellers | Qualitative, framework analysis. Interviews first, followed by workshops to design interventions. | The Irish Travellers were "least confident [of the English speakers] in their knowledge" about immunisation (xxiv). There is a variety of views across the groups about vaccination, e.g. some thought vaccination during pregnancy was fine, others didn't; some thought immunising teenage girls against human papillomavirus (HPV) would imply promiscuity, others didn't. Literacy was a barrier to acquiring knowledge about services; some English-speakers were not literate, while some Roma people had limited/no English. | Irish Traveller |
| Niksic, Rachet, Warburton & Forbes | 2016 | To examine ethnic group differences in awareness of cancer symptoms and barriers to accessing services | Secondary analysis of multiple surveys. Kruskal-Wallis. Logistic regression. | Overall, the findings were very similar between the White Irish and White British. White Irish (and White British) were better at recognising cancer symptoms than "ethnic minority groups" (p.138). Lower awareness of symptoms was associated with greater socio-economic deprivation across all ethnicities. In terms of barriers to healthcare, White Irish were significantly more likely than White British to say they were too busy to attend the doctor to discuss potential cancer symptoms, were more likely to say they were worried about many other things, and more likely to have transport difficulties. | White Irish. |
| Das-Munshi, Chang, Dutta, Morgan, Nazroo, Stewart & Prince | 2017 | To examine ethnic group differences in excess mortality among people with severe mental illness | Linkage of death records with South London and Maudsley (SLaM) case registry. Cox regression, hazard ratios, age-and-sex-standardized mortality ratio. | People with a severe mental illness had higher standardised mortality ratios than people without a mental illness, regardless of ethnicity. Findings for the Irish mostly not significantly different from White British, although in bivariate analysis the Irish had significantly higher hazard ratio of death from natural causes compared to White British (this became insignificant after controlling for the other variables in the model). South Asian and Black groups had lower mortality risk compared to the White British (and White Irish). | Irish included rather than White Irish. |
| Dixon, Mullis & Blumenfeld | 2017 | Comparison of vaccine uptake among Traveller and non-Traveller people in England | Audit of GP records. | Irish Traveller children had 40% lower vaccination coverage than non-Traveller children. Irish Traveller children are also less likely to complete the full expected set of vaccinations. | Irish Travellers |
| Jackson, C., Bedford, H., Cheater, F. M., Condon, L., Emslie, C., Ireland, L., Kemsley, P., Kerr, S., Lewis, H. J., Mytton, J., Overend, K., Redsell, S., Richardson, Z., Shepherd, C., Smith, L., & Dyson, L. | 2017 | To explore barriers to vaccine uptake among Gypsy Roma Traveller groups in England and Scotland | Qualitative, framework analysis. Interviews. | The participants who were adolescent girls from the London Irish Traveller group seemed to have less awareness of the HPV vaccine than other groups interviewed. There seemed to be an overall shift toward receiving health messages from healthcare professionals rather than from family, but the Irish Travellers still relied heavily on word-of-mouth. | Irish Traveller |
| Moore, Thornton & Hughes | 2017 | To understand how Irish survivors of institutional childhood abuse (ICA), and who later emigrated to Britain, seek help with their trauma | Participatory Action Research. Qualitative. Thematic analysis. | It was common for participants to rely on "self-management" (p.380) rather than seek help, despite reporting "depressive symptoms, nightmares, difficulties sleeping and flashbacks" (p.380). A minority were actively in therapy, and a slightly larger number wanted therapy in the future. Many had disclosed their experiences for the first time only recently, because of the setting up of the Residential Institutions Redress Board which could offer compensation to adult survivors of institutional abuse. They found the experience of giving evidence to the Board distressing. Some of them found it such a negative experience that it put them off seeking future help. Interestingly, living in the UK rather than Ireland meant that survivors could conceal their experience if they wanted to, because the institutions are not common knowledge in the UK, and questions about schooling, etc, would not be asked. Participants spoke about mainstream healthcare professionals having either limited knowledge or no knowledge at all about the extent of physical abuse and neglect that went on in the institutions. There was a strong aversion to authority figures and loss of control, which hindered help-seeking. Many participants did not know their family, or had little contact with them, and so were unable to answer health professionals' questions about whether certain health conditions were in their family. This led to great distress when such questions were asked repeatedly or their answers met with incomprehension by mainstream staff. | Irish born |
| Public Health England | 2017 | Report on public health outcomes framework focusing on ethnicity | Descriptive analysis | White Irish people have a high rate of admission to hospital for alcohol related issues (as do Other White and White British). Looking at country of birth, people born in RoI or NI (also Scotland) have significantly worse mortality rates than the average for England as a whole, and worse than any other country of birth. Suicide is high for people born in Ireland (also Scotland, Poland, Germany). Men born in Ireland have significantly higher premature cardiovascular disease mortality than England (as do EU Accession countries, Central and Western Africa, Southern Asia, Scotland, and South and Eastern Africa). Women are not significantly different from the English rate. Men born in Ireland have the worst rate of premature cancer mortality of all ethnic groups. Women born in Ireland have the third highest (after NI and Scotland). White Irish children have similar levels of excess weight to the English average. White Irish men and women have a disproportionate number of admissions to hospital for alcohol-related reasons compared to admission for all causes. Suicide rates are significantly higher among women and men born in Ireland compared with the England average. | White Irish. Irish born (RoI, NI). |
| Tilki | 2017 | To argue that the Irish with dementia have specific needs that mainstream services in the Britain need to address | Discussion article | Irish people with dementia have specific needs. These can be traced to migration experiences, including discrimination and negative experiences with authority. Travellers may develop dementia at earlier ages. Irish people may be stoic and reluctant to ask for help until crisis point. An Irish person with dementia in England may benefit from culturally specific services that include Irish symbols, staff with Irish accents, and familiar music from their youth. Staff need to be able to pronounce Irish names and to understand often strong Irish accents. Irish people from the Gaeltacht (Irish language speaking areas) might lose their second language (English) as dementia progresses. Irish people may have painful experiences in their past, which culturally competent staff can be prepared to hear and be sensitive to. Reminiscence activities may uncover painful memories that the staff do not expect. Even if Irish people have rejected the church, they may still find value and comfort in rituals around death such as wakes and memorial services. | Includes the Irish as an ethnic minority group and within the 'BAME' umbrella. |
| Wright, Rosato, Raab, Dibben, Boyle & O'Reilly | 2017 | To understand if mortality differences persist between Catholics and Protestants in Scotland and Northern Ireland, and if equality legislation has changed anything | Linkage of death records with the ONS Longitudinal Studies in Scotland and Northern Ireland. Mortality rates and incidence rate ratios. | Mortality risk was higher in Scotland than NI overall. Catholics were disadvantaged in Scotland compared to Protestants in terms of mortality, but there was no significant difference in mortality between Catholics and Protestants in Northern Ireland. The authors argued that this could have been due to the longer duration of equality legislation in NI. They also suggested that it could be related to persistent disadvantage caused by the migration of the Irish Catholics in the 1840s, whom most Scottish Catholics are descended from. NI Catholics didn't face the same migration history. | People of Catholic descent in Scotland and Northern Ireland. |
| Berlin, Smith & Newton | 2018 | To understand lay perceptions of cancer among Gypsies and Travellers | Qualitative. Focus group discussions with a critical constructivist approach. Boyatzis' type of thematic analysis. | The participants said they avoid using the word cancer. The authors related this to a belief that cancer cannot be cured. There was reluctance to approach a doctor because of past history of discrimination against Gypsies/Travellers. The Irish Travellers spoke about visiting a 'curing man' who specialises in cancer. The Irish Travellers also used prayer and penance to respond to cancer rather than use medical treatment. One of the English Gypsy participants spoke about continuing to use sun beds despite the risk of skin cancer because conforming to a notion of beauty was more important. The fear of getting cancer did not lead to the stigmatising of people who get cancer; everyone rallies around them. Care was strongly gendered; only females can wash females and males wash males. This would ideally be done by family members, but care workers can be utilised if there is no other option. Similarly, remaining at home is preferred to moving in into a care home or hospice, but they will be used as a last resort if there is no suitable informal carer available or if the care needs are too complex. | Irish Traveller |
| Bhopal, Gruer, Cezard, Douglas, Steiner, Millard, Buchanan, Vittal Katikireddi & Sheikh | 2018 | To assess ethnic group differences in mortality rates in Scotland | Linkage of death records 2001-2013 with the 2001 Census in Scotland. Age-adjusted mortality rate ratios. Part of the SHELS study. | White Irish males have similar mortality rate and rate ratios as do the White Scottish males, no significant differences either before or after controls added to the model. Some of the other ethnic groups had significantly lower rate ratios than White Scottish. Interestingly, mixed was the only group with higher mortality rate and rate ratios than White Scottish, but these still were not significantly different. White Irish women had significantly lower age-adjusted rate ratios compared with White Scottish women. Those born outside UK/RoI had lower age-adjusted rate ratios than White Scottish born in UK/RoI. | White Irish. But UK and RoI country of birth combined into one category. |
| Gruer, Millard, Williams, Bhopal, Katikireddi, Cezard, Buchanan, Douglas, Steiner & Sheikh | 2018 | To assess ethnic group differences in hospitalisation in Scotland | Linkage of hospital records 2001-2013 with the 2001 Census in Scotland. Part of the SHELS study. Poisson regression. Hospitalisation rate ratios. | White Irish men had significantly higher rate ratios of hospitalisation than White Scottish, but the difference became insignificant once age was controlled for. White Irish women also had significantly higher rates of hospitalisation than White Scottish women, but controlling for age caused them to have significantly lower rates of hospitalisation. Length of stay was also longer for White Irish men and women compared with White Scottish, but after controlling for age White Irish men and women had shorter lengths of stay than White Scottish. | White Irish. Country of birth. Also combines UK/RoI into a single country of birth category. |
| Heuvelman, Nazroo & Raj | 2018 | To assess ethnic group differences in rates of reporting psychotic symptoms. | Secondary analysis of the Fourth National Survey of Ethnic Minorities (1993/4) in England & Wales, and the EMPIRIC survey (2000/1) in England. Factor analysis. | White Irish participants had raised odds of reporting all types of psychotic symptoms compared with White British, but the difference was not significant. Caribbean and Pakistani individuals had significantly higher odds of overall psychotic symptoms compared with White British. | White Irish |
| Kapadia, Nazroo & Tranmer | 2018 | To examine ethnic group differences and influence of social networks in mental health service use | Secondary analysis of the EMPIRIC survey 2000. Logistic regression. | At bivariate level White Irish women had the highest percentage (13%) using mental health services in the last six months. White Irish women had the highest percentage of positive aspects of support; second lowest percentage having frequent face-to-face contact with their relatives; second highest percentage with no frequent face-to-face contact with relatives. At multivariate level White Irish women not significantly different from White reference group in using mental health services (although the OR in each case was higher than 1.00). For all ethnic groups, having inadequate levels of social support was related to higher rates of using mental health services, and having frequent face-to-face contact with relatives was related to lower rates of using mental health services. | White Irish. Compares 'White' with 'White Irish'. |
| Moore, Tilki, Clarke & Waters | 2018 | To examine the process whereby social support is beneficial for health among Irish people in London | Data were collected in 2011, part of a larger action research project involving Irish in Britain. Hierarchical logistic binary regression. Moderation effects tested with interaction terms. | There was a significant association (chi-squared test) between poor social support and fair/poor health; also between poor social support and unfair treatment. One fifth (21.4%) of the sample had experienced "unfair treatment" in the last 2 years, and this was significantly more likely among those born in Ireland. Those with more sources of support had better self-rated health in the regression model. Better availability of practical help from neighbours had a moderating effect, whereby it reduced the negative effect of unfair treatment on self-rated health. In addition, women experiencing adversity showed an even greater benefit of support from neighbours on self-rated health. | People who self-identified as Irish. |
| Das-Munshi, Chang, Schofield, Stewart & Prince | 2019 | To check for ethnic group differences in mortality related to depression | Linkage of records from secondary mental health services with death certificates. Standardised mortality ratios and hazard ratios. Analysis controlled for salmon bias. | Overall, people with depression have a higher mortality risk than people in the general population. The Irish group is the only ethnic group with significantly higher hazard ratio of death (all causes) compared to the White British. All other ethnic groups are significantly lower than White British. When looking at 'unnatural cause mortality in unipolar depression' in the regression the Irish had a higher hazard ratio than WB but it was not significantly different. Looking at SMRs only, the Irish had an "elevated" risk. | Irish |
| Delgado-Angulo, Mangal & Bernabe | 2019 | To understand whether socio-economic inequalities explain ethnic group differences in oral health. | Secondary analysis of the Health Survey for England, pooled 1999, 2000, 2001, 2002, 2005 datasets. | The Irish group had significantly higher odds of experiencing toothache compared with the White British reference group, and controlling for confounding factors. Only the Caribbean group also had significantly higher odds. Bangladeshis had significantly lower odds of toothache. The Irish were not significantly different from the White British in terms of edentulousness (having no teeth at all). Most of the other minority ethnic groups were significantly less likely than the White British to be edentulous. Ethnic inequalities in oral health are not fully explained by socio-economic position. | Irish |
| Moore | 2019 | To test the main effects hypothesis of social support on health among Irish people in London | ANOVA. Hierarchical linear regression with interaction terms. | Being able to rely on the support of at least 3 people in a crisis was significantly associated with self-rated health in the final regression model. Emotional support was not significant. People whose source of advice with a health problem was an Irish community organisation were six times less likely to be in good health. At the bivariate level, younger adults had less social support, possibly indicating less opportunity to build a social network among new migrants. | People who self-identified as Irish. |
| Moore, Flynn & Morgan | 2019 | To compare resilience and mental wellbeing among survivors of Irish clerical institutional abuse who emigrated and who did not emigrate | Quantitative survey (all participants) and a structured interview (with 9 participants). MANOVA. Regression. Content analysis. | At the bivariate level, mental wellbeing was higher among the UK-dwelling sample than the Irish-dwelling sample. Other factors associated with lower mental wellbeing were being male, unemployment and being in an unskilled occupation, as well as being in a second or third relationship. On the resilience scale, again the UK-dwelling sample scored higher than the Irish-dwelling sample. Some features of resilience (personal skills and competencies; social and community inclusion) significantly predicted mental wellbeing in the regression model. Country of residence was not significant in the regression analysis. In the qualitative analysis, moving to the UK was seen as a turning point opening doors to greater employment opportunities and the freedom that comes from having choices and income. One of the coping strategies adopted was defiance, in the sense of being determined to make something of oneself even though they had been told they were useless by the institutions. Another was altruism, which seemed to sometimes be detrimental to the survivor, but they were doing their best to help prevent harm to others. Migrating to the UK had positive effects in a variety of ways, one of which was the anonymity it afforded. The participants could make a new identity for themselves that was not affected by other people's knowledge that they were a survivor. The authors point out that adult romantic relationships were not a source of resilience for the survivors; this is perhaps contrary to studies of resilience and wellbeing factors among other populations. Some of the strategies adopted by survivors (defiance, altruism) may seem maladaptive to practitioners who are not aware of the context of institutional abuse survivors; the authors advocate for greater awareness raising among health care practitioners. | Country of birth. |
| Aldridge, Lewer, Katikireddi, Mathur, Pathak, Burns, Fragaszy, Johnson, Devakumar, Abubakar, Hayward | 2020 | To examine risk of death from COVID-19 among people from minority ethnic groups. | Secondary analysis of hospital death records. Standardised mortality ratios adjusted for age and region. | White Irish and White British groups had a lower risk of death from COVID-19 compared with the population as a whole, even after adjusting for region and age. Black African, Black Caribbean, Pakistani, Bangladeshi, and Indian groups all had a higher risk of death compared with the whole population. No difference between the general population and Mixed or Chinese groups. | White Irish. |
| Anselmi, Everton, Shaw, Suzuki, Burrows, Weir, Tatarek-Gintowt, Sutton & Lorrimer | 2020 | To estimate the need for mental health and learning disability services in England based on population characteristics in each area | Secondary analysis of health service records. Linear regression used to estimate cost of care based on particular characteristics. Then creation of a new formula to estimate cost. | Irish people had higher costs for mental health services per year than White British by £34, and some other ethnic groups had much higher costs (e.g. Black Caribbean £134 higher). People from South Asian backgrounds and Chinese backgrounds had significantly less spent on them in terms of mental health services. Costs by age were highest for people aged between 20 and 45, indicating older people have much less spent on them in terms of mental health care. People living in care homes had the highest costs, followed by people in communal households, and then people living alone, all compared with people living in a two-person opposite-sex household. | Irish |
| Cezard, Gruer, Steiner, Douglas, Davis, Buchanan, Katikireddi, Millard, Sheikh & Bhopal | 2020 | To compare falls and road traffic injuries that lead to hospitalisation or death in Scotland across ethnic groups | Secondary analysis of the Scottish Health and Ethnicity Linkage Study (Census 2001 linked to death records and hospital records). Age-adjusted risk ratios (RRs). Poisson regression. | For males and females, White Irish group had significantly higher age-adjusted relative risks of hospitalisation and death due to falls compared with the reference group (White Scottish). Adjusting for SES and country of birth did not change the significance. For hospitalisation and death related to road traffic injuries, White Irish not significantly different from White Scottish for either males or females. Several of the "non-White" groups had much lower risks of fall/RTI related hospital admission or death. | White Irish. Country of birth (RoI). Collapse RoI/UK together. |
| Delgado-Angulo, Zuniga Abad, Scambler, Bernabe | 2020 | To find out whether migrants have better oral health than British born people, and if this can be explained by the healthy migrant effect. | Secondary analysis of the Health Survey for England 1999. | 9.2% of the sample had no teeth. 24% of those with teeth had experienced toothache in the last 6 months. Edentulousness was more common among first-generation Irish than White British. Second-generation Irish had higher odds of toothache than White British. First generation Irish not significantly different from WB on toothache. | Irish |
| Lowe, Payton, Verma, Gemmell, Worthington, Hamilton, Ollier, Augustine & Poulton | 2020 | Relationship between human leukocyte antigens and renal function among minority ethnic groups | Secondary analysis of data from the UK Biobank. Regression. | Among the Irish participants, a certain allele (HLA-DRB1*04:01) was significantly associated with increased (i.e. better) estimated glomerular filtration rate (eGFR) and decreased risk of chronic kidney disease. Among some of the other ethnic groups there were alleles associated with decreased (i.e. worse) eGFR or increased eGFR in a couple of cases. There is variation between ethnic groups in how the alleles function; among some an allele is good for kidney function, among others it seems to be bad. There is an interaction. | Irish. Paper only examines ethnic minority groups ('BAME') so Irish included as an ethnic minority. |
| Mansour, Tsamakis, Rizos, Perera, Das-Munshi, Stewart & Mueller | 2020 | Comparing how depression presents and is managed among older people from different ethnic groups | Medical records study of a 'large mental health care provider in South London'. Data from 1/1/2006 - 30/6/2017. Chi-square. Kruskal-Wallis. ANOVA. Logistic regression. | Symptoms: White Irish were the most common to record substance use. Non-accidental self-injury was highest among Black Africans. Highest rate of recurrent depressive disorder diagnoses were among the White Irish. Lowest rate of physical health problems, activities of daily living (ADL) problems, and psychotic symptoms reported among the White Irish. Highest rate of guilt feelings, poor motivation, concentration problems, suicidal thoughts among the White Irish. Black patients more likely to have psychotic symptoms. In regression, White Irish had significantly higher odds of disturbed sleep compared with WB. Treatment: Being prescribed antidepressants and cognitive behavioural therapy was highest for the White Irish, and significantly less likely for the Black African patients (compared with White British). | White Irish. Compares "ethnic minorities" with "White British". |
| Morris, Sellwood, Edge, Colling, Stewart, Cupitt & Das-Munshi | 2020 | To examine likelihood of receiving cognitive behavioural therapy (CBT) for psychosis or bipolar disorder between different ethnic groups | Medical records study from one catchment area in London. Data from Jan 2007 to July 2017. Logistic regression. | Findings show that Black Africans are significantly less likely to receive CBT than White British. Most of the analyses also showed Black Caribbean being significantly less likely than White British. The White Irish group was the oldest in the sample. They also had the highest percentage with a lifetime comorbid substance use diagnosis. In the multivariate analyses predicting different numbers of CBT sessions, the White Irish was not significantly different from the White British. | Irish (not White Irish) is included, as is White British. |
| Cook | 2021 | To offer potential reasons for higher COVID death rates among Black and Minority Ethnic (BME) people | Letter to the editor | They discuss the figures from the Institute for Fiscal Studies on number of deaths in hospital from COVID-19 by ethnicity. The White Irish had "half the number of fatalities" compared to White British. The author argues that haemochromatosis might be responsible. Haemochromatosis is higher among the White Irish than the White British. This may make them less likely to become anaemic, and therefore less likely to suffer from hypoxaemia (which is a complication of COVID-19). Therefore, the Irish with haemochromatosis may be protected from the risk of death from COVID-19. People with sickle cell and thalassaemia are at higher risk. | White Irish |
| Polling, Bakolis, Hotopf & Hatch | 2021 | To examine ethnic group differences in rates of hospital admission for self-harm | Hospital Episode Statistics, 1/4/2008-31/3/2018. Rate ratios. Log-binomial regression. | White Irish, Mixed Other, Other all had higher rates of admission for self-harm compared with White British, for White Irish this was still significantly higher even after standardisation for age and SES. White Irish also significantly more likely to be readmitted a year later compared with White British. Most of the other ethnic groups had lower rates of admission for self-harm. | White Irish. |
| Roman-Urrestarazu, van kessel, Allison, Matthews, Brayne & Baron-Cohen | 2021 | To look for patterns related to autism with ethnicity and social deprivation among children | Secondary analysis of the Spring School Census 2017. | 1.76% of the sample had autism spectrum disorder (ASD). More than four times as many boys than girls had ASD. ASD was highest among the Black group and lowest among Roma/Irish Traveller groups. Chinese and Black pupils were significantly more likely to have ASD when compared to White pupils in a regression analysis controlling for other variables. | Roma/Irish Travellers. |
| Shafiq, Parveen & Oyebode | 2021 | To review the literature in order to find out how African Caribbean or Irish people cope with health conditions, aiming to find "culturally preferred coping strategies". | Systematic review. CASP for quality assessment. | They found 26 relevant articles; 24 on African Caribbeans and 5 on Irish people; some of these included both groups so that is why they are counted twice. Coping strategies included denial, avoidance/scepticism, self-management, spirituality/religion. Barriers to managing the chronic condition included stigma, fear, mistrust. The Irish findings about stigma and fear were in relation to gay men in Northern Ireland with HIV being worried about what religious people would think of them. The mistrust findings were only found among the African Caribbean group. Religious coping was found among the Irish, even those who considered themselves no longer Catholic. Very limited information about Irish people in the literature reviewed. | Irish |
| Stopforth, Kapadia, Nazroo & Becares | 2021 | To assess health inequalities among older people across different ethnic groups and over time. | Harmonised six datasets that include data from 1993-2017 | Irish not significantly different from White/White British in terms of LLTI in any of the surveys in the logistic regression analyses. Irish do have significantly higher rates of fair/poor self-rated health compared with the White/White British but only in the Health Survey for England 1999. Analyses are not done separately for men and women, but does control for sex. In the vast majority of the analyses the Irish are almost indistinguishable from the pattern of the White/White British. The worst health outcomes are among the Pakistani and Bangladeshi groups, and these persist over time. | Irish (not White Irish) |
| Tsamakis, Gadelrab, Wilson, Bonnici-Mallia, Hussain, Perera, Rizos, Das-Munshi, Stewart & Mueller | 2021 | To examine disability and prescriptions of people with dementia from minority ethnic backgrounds | Analysis of medical records (Clinical Record Interactive Search system). Regression. | All of the sample had a diagnosis of dementia. Descriptive analyses: The White British group was the oldest. White Irish patients were the least likely to be currently married. Substance use (incl. alcohol) was most likely in the White Irish. ADL difficulties most likely among Irish and Black groups. Recreational/occupational activity difficulties least likely among Irish group. Antipsychotic medication prescribing was highest among the White Irish. White Irish also most likely to be prescribed antidepressants. Multivariate analyses: White Irish significantly more likely than White British to have substance/alcohol use and vascular dementia, and polypharmacy. This was even controlling for demographics, cognition, and social deprivation. The Black African, Black Caribbean, and White Irish groups having significantly higher odds of vascular dementia indicates these groups have higher vascular risk factors, and this could be an area for intervention. Alcohol misuse is a vascular risk factor. | White Irish |
| Watkinson, Sutton & Turner | 2021 | To examine ethnic group differences in health-related quality of life among people aged 55 and over in England | Secondary analysis of the English General Practice Patient Survey (2014-2017) | Compared to the White British/Northern Irish reference group, Irish men (not women), Gypsy or Irish Traveller men (and women) had worse Health Related Quality of Life (HRQoL) (as did lots of other ethnic groups), but Chinese men and Black African men had significantly better HRQoL. Irish participants had lower odds of reporting poor experiences in primary care compared with White British/NI. The finding of worse quality of life (QoL) for Irish men than women is contrary to the prevailing finding that women have worse health than men in this study. White Irish men and women had significantly more social deprivation than White British; significantly higher odds of reporting low self-confidence in managing own health; no difference in support from local services in managing health conditions. Irish men had significantly more long-term health conditions; Irish women no different from WB women. White Irish men had significantly higher odds of reporting poor quality of life in the domains of self-care and anxiety/depression compared with White British/NI men. White Gypsy or Irish Traveller men had significantly higher odds of reporting poor QoL in all five QoL domains. White Irish women had significantly lower odds of reporting poor QoL in the domains of mobility and usual activities, but significantly higher odds of poor QoL in the self-care domain (compared with WB/NI women). White Gypsy or Irish Traveller women had significantly higher odds of reporting poor QoL in all five QoL domains. White Irish men had significantly higher odds of reporting more long-term conditions compared with WB men; women not significantly different. White Gypsy or Irish Traveller both men and women significantly higher odds. White Irish men significantly less likely than WB to have 'any other long-term condition'. White Gypsy or Irish Traveller men significantly more likely than WB to have diabetes or long-term back problems. White Irish women significantly more likely than WB to have high blood pressure or long-term back problems, but significantly less likely than WB to have diabetes or 'any other long-term condition'. White Gypsy or Irish Traveller women significantly more likely to have 'any other long term condition' but not significant on any of the other conditions. White Irish men significantly more likely than WB/NI to have asthma, cancer, long-term MH problems, and Alzheimer's or other dementia. White Gypsy or Irish Traveller men only significantly more likely to have asthma. White Irish women significantly more likely than WB to have asthma, angina, cancer, long-term mental health problems, and Alzheimer's, but sig less likely to have deafness. White Gypsy or Irish Traveller women significantly more likely to have asthma or mental health problems. | White Irish, White Gypsy or Irish Traveller |
| Alobaidi, Bernabe & Delgado-Angulo | 2022 | To see if ethnic inequalities in oral health can be explained by deprivation | Secondary analysis of the Health Survey for England 2010/11 | In multivariate analysis the Irish had significantly higher odds of "non-functional dentition" compared with the White British, even after controlling for age, sex, residence area, socio-economic position, and area deprivation. The Irish were not significantly different from the WB in terms of poor self-rated oral health or oral impacts. "Our findings showed that Irish adults are the ethnic minority at the greatest disadvantage" (p.163). | Irish described as an ethnic minority group (not White Irish) |
| Chum, Teo & Azra | 2022 | To examine the relationship between mental health and neighbourhood cohesion by ethnicity | Secondary analysis of the UK Household Longitudinal Survey (Understanding Society) 2009-2018 (Waves 1, 3, 6, 9). Longitudinal fixed effect analysis. | The hypothesis that improved neighbourhood cohesion would be related to improved General Health Questionnaire (GHQ) score was upheld for many (but not all) ethnic groups. Irish was not significantly different from White British in this relationship. At bivariate level the Irish had a higher (worse) GHQ score than the White British, and of the 17 ethnic groups only six had the same or higher GHQ score than the Irish. The worst GHQ was among the Arab and Other groups. | Uses a category called 'Irish (Republic)', but later calls them 'Irish Whites' so assumes only white people are included in this category. |
| Moore | 2022 | To examine whether there are gender differences in how Irish people in London use the general practitioner (GP) | This sample was used for previous papers by this author. Data collected in 2011. | Older people were more likely than younger people to seek help regarding health issues from community-based organisations. There was an interaction between gender and helpseeking: for women as self-rated health got worse the likelihood of accessing the GP increased; for men as self-rated health got worse the likelihood of accessing the GP reduced. However, increased social support increased the likelihood of men accessing the GP; it made no difference for women. | Irish |
| Pickett, K. E., Ajebon, M., Hou, B., Kelly, B., Bird, P. K., Dickerson, J., Shire, K., McIvor, C., Mon-Williams, M., Small, N., McEachan, R., Wright, J., & Lawlor, D. | 2022 | To examine child well-being among different ethnic groups in Bradford | The Born in Bradford study | Gypsy/Traveller children significantly more likely than White British children to not have three meals a day, and more likely to worry all the time about money. Gypsy/Traveller children were significantly less likely to be bullied, and significantly more likely to be mean to others (compared with White British). Gypsy/Traveller children significantly more likely than White British to be unable to know what to do when things are hard. | Gypsy/Irish Traveller. Other Irish people are lost within the categories. |
| Thompson, Stone & Tyson | 2022 | To understand the mental health needs of Gypsy, Roma, and Travellers in the British Isles | Qualitative interviews. Thematic analysis. | The participants had experienced racism from health care services, social services, schools, and wider society. One Irish Traveller was gay and spoke of being ostracised from his own group as well as wider society. Many participants had witnessed violence or experienced violence within their own site or family. The practicalities of Gypsy, Roma, Traveller life made attending mental health appointments difficult, e.g. men worked long hours, women were expected to focus on childcare and cleanliness; telephone or home visits were inappropriate because of the lack of privacy; women might need permission to attend an appointment or be chaperoned. Having mental illness in the family was seen as detrimental to marriage prospects, so people refused to talk about it. Participants were afraid to seek help for mental illness because they thought their children might be removed. | Irish Traveller |
| Amininia, Bernabe & Delgado-Angulo | 2023 | To understand the relationship between social support and oral health among people in England from different ethnic groups | Secondary analysis of the Health Survey for England 1999-2002/2005. Chi-squared test. Regression with mediation analysis. | Irish scores on social support were very similar to the White British at bivariate level. More of the Irish were in the wealthiest quintile than any other ethnic group, including White British, although the Irish also had more people in the poorest quintile than White British. The Irish were the most likely to be edentulous. Edentulousness was more likely among the formerly married than among married or single people. At multivariate level Irish people were significantly more likely to be single or never married than white British people. Irish people were not different from the White British in social support, but every other ethnic group reported significantly less social support than the White British. At multivariate level the Irish were significantly more likely to have toothache compared with White British, but not significantly more likely to be edentulous. Overall conclusion is that social support did not explain ethnic group differences in oral health, so it was not a mediator, but the main effects hypothesis was upheld: "perceived lack of social support is negatively associated with adult oral health over and above the effects of sociodemographic factors" (p.65) | Irish included as one of the "main six ethnic minority groups in the UK" (p.61). Note, not 'white Irish'. |
| Co, Mueller, Mayston, Das-Munshi & Prina | 2023 | To look for ethnic differences in survival time after receiving a diagnosis of dementia | Secondary analysis of patient records (London) linked to ONS death data (2008-2017). Standardised mortality ratios, Kaplan-Meier survival curves, and cox regression models. | The whole cohort had higher standardised mortality rate compared with the general population. The SMRs differed for each ethnic group, with White Irish and Black African having the highest SMRs. However, comparing between the ethnic groups in the sample, all ethnic groups had a lower unadjusted hazard of death compared to White British. Adding in covariates adjusted the scores slightly, but the White Irish remained significantly lower than White British. When looking at dementia subtypes, White Irish and White British with mixed and Alzheimer's subtypes were not significantly different in hazard of death, but White Irish had significantly lower hazard with vascular and 'other' types of dementia. At ages 65-75 and 75-85, no difference in hazard of death between White British and White Irish. | White Irish |
| Irish in Britain | 2023 | To examine the profile of Irish people in the 2021 Census of England and Wales. | Secondary analysis of the 2021 Census | In 2021 there were 523,014 "Irish-born" people in England and Wales, which includes people born in the Republic of Ireland or Northern Ireland. The ethnic group of White Irish was 507,465, while the ethnic group of Gypsy or Irish Traveller was 67,800. Absolute number of Irish-born and White Irish has reduced in number from the 2011 Census, while Gypsy or Irish Traveller has increased in number. Percentage of White Irish in the population of England and Wales has not changed from 2011 (at 0.9%). The White Irish ethnic group still has an older age structure than the White British ethnic group. The White Gypsy or Irish Traveller group has a younger age structure than White British. The White Irish (43.4%) ethnic group has a larger proportion of people with higher-level qualifications compared to the White British (30.8%) (national average 33.8%), but also a slightly larger percentage with no formal qualifications (20.2%) compared to WB (18.3%) (national average 18.2%). The pattern for Gypsy or Irish Traveller shows extreme inequality (56.9% no formal qualifications, 11.1% higher level qualifications). White Irish is similar to WB in percentage employed (61.9% compared with 61.6%) but has fewer economically inactive (21% compared with 23.5% for WB). Gypsy or Irish Traveller has a large percentage of economically inactive (53.3%). Reasons for economic inactivity are being student, homemaker or caregiver, long-term sick or disabled, retired, or other. Largest percentages for Gypsy or Irish Traveller are homemaker or caregiver (17.7%) and long-term sick and disabled (15.0%). Figures for White British and White Irish are much lower in all categories, but White Irish are less likely to be students or homemaker/caregiver than White British, and more likely to be retired. White Irish (49.9% for men, 50.0% for women) who were employed had higher percentages of being in manager/professional occupations than either White British (33.8% for men, 31.1% women) or the national average (33.9% for men, 32% for women). Gypsy or Irish Traveller much lower (17% men, 15.1% women). Figures for health and disability do not control for age. White Irish and Gypsy or Irish Traveller had higher rates of bad or very bad health (7.7% White Irish, 12.6% Gypsy or Irish Traveller) compared with national average (5.2%) or White British (5.9%). White Irish (41.7% men, 41.6% women) actually had the lowest percentage reporting 'very good' health, lower even than Gypsy or Irish Traveller (45.9% men, 43.8% women), (White British 47.0% men, 45.0% women). White Irish had higher percentages of limiting disability (20.2% men, 24% women) than national average. Gypsy or Irish Traveller even higher. White Irish provide similar levels of unpaid care to the White British population, but more than the national average. Gypsy or Irish Traveller much higher, especially for women carers. White Irish people aged 65+ are more likely to live in social rented housing (19.1%) than the national average (13.1%), although looking at all ages White Irish is less likely than general population to live in social rented housing. Suggest important vulnerability among older Irish population in England & Wales. Overcrowded accommodation is much worse among Gypsy or Irish Traveller (26.3%) than White Irish (3.5%), White British (4.4%) or national average (8.4%). | Country of birth (RoI or NI). Ethnicity (White Irish, Gypsy or Irish Traveller). Passport held (Irish passport). National identity (Northern Irish only, Northern Irish and British, Irish only, Irish and at least one UK identity) |
| Finney, Nazroo, Becares, Kapadia & Shlomo | 2023 | To report on the findings of the Evidence for Equality National Survey (EVENS). Aimed to examine experiences during COVID-19 pandemic and make comparisons between ethnic and religious minority groups. | Primary data collection. Survey collected Feb – Nov 2021. Great Britain (not Northern Ireland). | Chapter 5 reports on health and mental wellbeing data. Self-rated health, limiting long-term illness, depression, anxiety, chronic conditions, COVID-19 infection and symptoms, access to services, caregiving and receipt, vaccination, bereavement. White Irish had higher risks of anxiety (GAD) and of increased loneliness (due to the pandemic) compared with White British, but Gypsy/Traveller less likely to experience loneliness than White British. Gypsy/Traveller men had higher risk of physical multimorbidity compared with White British men. White Irish had greater access to health services than White British during the pandemic, Gypsy/Traveller no different from White British (but Roma less likely to access). White Irish and Gypsy/Traveller groups both had higher rates of COVID-19 infection than White British, and also higher rates of being bereaved due to COVID. | White Irish, Gypsy/Traveller (Roma has a separate category). White Irish and Gypsy/Traveller among the most likely to say their ethnic background is important to their sense of identity. |
