# Supplementary material for: Where Are the Irish in Research on Ethnic Health Inequalities in Britain? A State‐Of‐The‐Art Literature Review
Source: Sociol Health Illn. 2025 Jan 2;47(1):e13874. doi: 10.1111/1467-9566.13874 (PMC11694090; doi:10.1111/1467-9566.13874)
Supplement: Supplementary file 3 — Supporting Information S3 [file SHIL-47-0-s003.docx]

**Online Appendix 3: Search Strategy Tables**

Table A1: Search history in MEDLINE

| **Search type** | **MEDLINE (EBSCO) search within title and abstract** | **Hits** | **2001-2023** |
| --- | --- | --- | --- |
| Search 1: Irish health status  *(italics = subject headings within MEDLINE)* | (Irish OR Ireland OR “Irish in Britain”) OR *Ireland/ethology*  AND  (“Great Britain” OR Britain OR England OR Scotland OR Wales OR British OR Scottish OR Welsh) OR *United Kingdom*  AND  (emigrat* OR immigrat* OR emigrant* OR immigrant* OR diaspor* OR ethnic* OR BME OR BAME OR minorit* OR racial*) OR *ethnicity* OR *ethnic and racial minorities* OR *Emigration or immigration*  AND  (“self-rated health” OR “self-reported health” OR “quality of life” OR QoL OR wellbeing OR “well-being” OR “physical health” OR “mental health” OR “mental illness*” OR “mental disorder*” OR depress* or anxi* OR schizophren* OR psychosis OR psychotic OR addict* OR alcohol* OR misuse* OR substance* OR “self-harm*” OR suicide* OR mortality OR morbidity OR disab* OR functional OR ADL OR IADL OR “long-standing illness” OR “long-term illness” OR dement* or cognit* OR Alzheimer* OR haemochromatosis OR covid* OR corona*) OR health* N1 (status OR survey* OR inequit* OR inequal*) OR *Health* OR *Health status* OR *health surveys* OR *healthcare disparities* OR *health status disparities* OR *health inequities* OR *Mental Health* OR *Depression* OR *Anxiety* OR *Schizophrenia* OR *Psychotic Disorders* OR *Substance-Related Disorders* OR *Self-Injurious Behavior* OR *Suicide* OR *Dementia* OR *COVID-19* | 247 | 189 |
| Search 2: Irish access to services *(italics = subject headings within MEDLINE)* | (Irish OR Ireland OR “Irish in Britain”) OR *Ireland/ethology*  AND  (“Great Britain” OR Britain OR England OR Scotland OR Wales OR British OR Scottish OR Welsh) OR *United Kingdom*  AND  (emigrat* OR immigrat* OR emigrant* OR immigrant* OR diaspor* OR ethnic* OR BME OR BAME OR minorit* OR racial*) OR *ethnicity* OR *ethnic and racial minorities* OR *Emigration or immigration*  AND  Health* N1 (attitude* OR satisf* OR accept* OR access*) OR *health services accessibility* OR *health knowledge, attitudes, practice* OR *patient acceptance of health care* OR *health behavior* OR *attitude to health* | 50 | 41 |

Table A2: Search history in PsycINFO

| **Search type** | **PsycINFO (EBSCO) search within title and abstract** | **Hits** | **2001-2023** |
| --- | --- | --- | --- |
| Search 1: Irish health status  *(italics = subject headings within PsycINFO)* | (Irish OR Ireland OR “Irish in Britain”)  AND  (“Great Britain” OR Britain OR “United Kingdom” OR UK OR England OR Scotland OR Wales OR British OR Scottish OR Welsh)  AND  (emigrat* OR immigrat* OR emigrant* OR immigrant* OR diaspor* OR ethnic* OR BME OR BAME OR minorit* OR racial*) OR *Human Migration* OR *Immigration* OR *Minority Groups* OR *Social Identity* OR *Cross Cultural Differences* OR *Racial Disparities* OR *Racial and Ethnic Groups* OR *Racial and Ethnic Differences* OR *Racial and Ethnic Attitudes* OR *Race and Ethnic Discrimination* OR *Ethnic Identity* OR *Ethnic Diversity*  AND  (“self-rated health” OR “self-reported health” OR “quality of life” OR QoL OR wellbeing OR “well-being” OR “physical health” OR “mental health” OR “mental illness*” OR depress* OR anxi* OR schizophren* OR psychosis OR psychotic OR alcohol* OR misuse* OR addict* OR substance* OR “self-harm*” OR suicid* OR mortality OR morbidity OR diagnos* OR disab* OR functional OR ADL OR IADL OR “long-standing illness” OR “long-term illness” OR dement* OR cognit* OR Alzheimer* OR haemochromatosis OR covid* OR corona*) OR health* N1 (status OR survey* OR inequit* OR inequal*) OR *Health* OR *Health status* OR *Health Disparities* OR *Mental Health Disparities* OR *Well Being* OR *Mental Health* OR *Mental Disorders* OR *Major Depression* OR *Anxiety* OR *Schizophrenia* OR *Psychosis* OR *Addiction* OR *Nonsuicidal Self-Injury* OR *Suicide* OR *Dementia* OR *COVID-19* | 207 | 148 |
| Search 2: Irish access to services *(italics = subject headings within PsycINFO)* | (Irish OR Ireland OR “Irish in Britain”)  AND  (“Great Britain” OR Britain OR “United Kingdom” OR UK OR England OR Scotland OR Wales OR British OR Scottish OR Welsh)  AND  (emigrat* OR immigrat* OR emigrant* OR immigrant* OR diaspor* OR ethnic* OR BME OR BAME OR minorit* OR racial*) OR *Human Migration* OR *Immigration* OR *Minority Groups* OR *Social Identity* OR *Cross Cultural Differences* OR *Racial Disparities* OR *Racial and Ethnic Groups* OR *Racial and Ethnic Differences* OR *Racial and Ethnic Attitudes* OR *Race and Ethnic Discrimination* OR *Ethnic Identity* OR *Ethnic Diversity*  AND  Health* N1 (attitude* OR satisf* OR accept* OR access*) OR *Health Attitudes* OR *Health Service Needs* OR *Health Care Services* OR *Health Care Access* OR *Health Service Needs* | 29 | 19 |

Table A3: Search history in Web of Science

| **Search type** | **Web of Science search within topic** | **Hits** | **2001-2023** |
| --- | --- | --- | --- |
| Search 1: Irish health status | Irish OR Ireland OR “Irish in Britain”  AND  “Great Britain” OR Britain OR “United Kingdom” OR UK OR England OR Scotland OR Wales OR British OR Scottish OR Welsh  AND  migrat* OR emigrat* OR immigrat* OR emigrant* OR immigrant* OR diaspor* OR ethnic* OR BME OR BAME OR minorit* OR racial*  AND  Health OR “self-rated health” OR “self-reported health” OR “quality of life” OR QoL OR well-being OR “physical health” OR “mental health” OR “mental illness*” OR “mental disorder*” OR depress* OR anxi* OR schizophren* Or psychosis OR psychotic OR addict* OR alcohol* OR misuse* OR substance* OR “self-harm*” OR suicid* OR mortality OR morbidity OR disab* OR functional OR ADL OR IADL OR “long-standing illness” OR “long-term illness” OR dement* or cognit* OR Alzheimer* OR haemochromatosis OR covid* OR corona* OR health* NEAR (status OR survey* OR inequit* OR inequal* OR disparit*) | 691 | 633 |
| Search 2: Irish access to services | Irish OR Ireland OR “Irish in Britain”  AND  “Great Britain” OR Britain OR “United Kingdom” OR UK OR England OR Scotland OR Wales OR British OR Scottish OR Welsh  AND  migrat* OR emigrat* OR immigrat* OR emigrant* OR immigrant* OR diaspor* OR ethnic* OR BME OR BAME OR minorit* OR racial*  AND  Health* NEAR (attitude* OR satisf* OR accept* OR access*) | 45 | 43 |
